# Novel insights on saccharin- and acesulfame-based carbonic anhydrase inhibitors: design, synthesis, modelling investigations and biological activity evaluation

**DOI:** 10.1080/14756366.2020.1828401

**Published:** 2020-10-02

**Authors:** Paolo Guglielmi, Giulia Rotondi, Daniela Secci, Andrea Angeli, Paola Chimenti, Alessio Nocentini, Alessandro Bonardi, Paola Gratteri, Simone Carradori, Claudiu T. Supuran

**Affiliations:** a Dipartimento di Chimica e Tecnologie del Farmaco, Sapienza University of Rome, Rome, Italy; b Neurofarba Department, Section of Pharmaceutical and Nutraceutical Sciences, University of Florence, Sesto Fiorentino, Italy; c Centre of Advanced Research in Bionanoconjugates and Biopolymers Department, “Petru Poni” Institute of Macromolecular Chemistry, Iasi, Romania; d Neurofarba Department, Section of Pharmaceutical and Nutraceutical Sciences, Laboratory of Molecular Modeling Cheminformatics & QSAR University of Florence, Sesto Fiorentino Italy; e Department of Pharmacy, “G. d’Annunzio” University of Chieti-Pescara, Chieti, Italy

**Keywords:** Saccharin, acesulfame, triazole, docking, carbonic anhydrase inhibitors

## Abstract

A large library of saccharin and acesulfame derivatives has been synthesised and evaluated against four isoforms of human carbonic anhydrase, the two off-targets hCA I/II and the tumour related isoforms hCA IX/XII. Different strategies of scaffold modification have been attempted on both saccharin as well as acesulfame core leading to the obtainment of 60 compounds. Some of them exhibited inhibitory activity in the nanomolar range, albeit some of the performed changes led to either micromolar activity or to its absence, against hCA IX/XII. Molecular modelling studies focused the attention on the binding mode of these compounds to the enzyme. The proposed inhibition mechanism is the anchoring to zinc-bound water molecule. Docking studies along with molecular dynamics also underlined the importance of the compounds flexibility (e.g. achieved through the insertion of methylene group) which favoured potent and selective hCA inhibition.

## 1. Introduction

**Figure 1.**
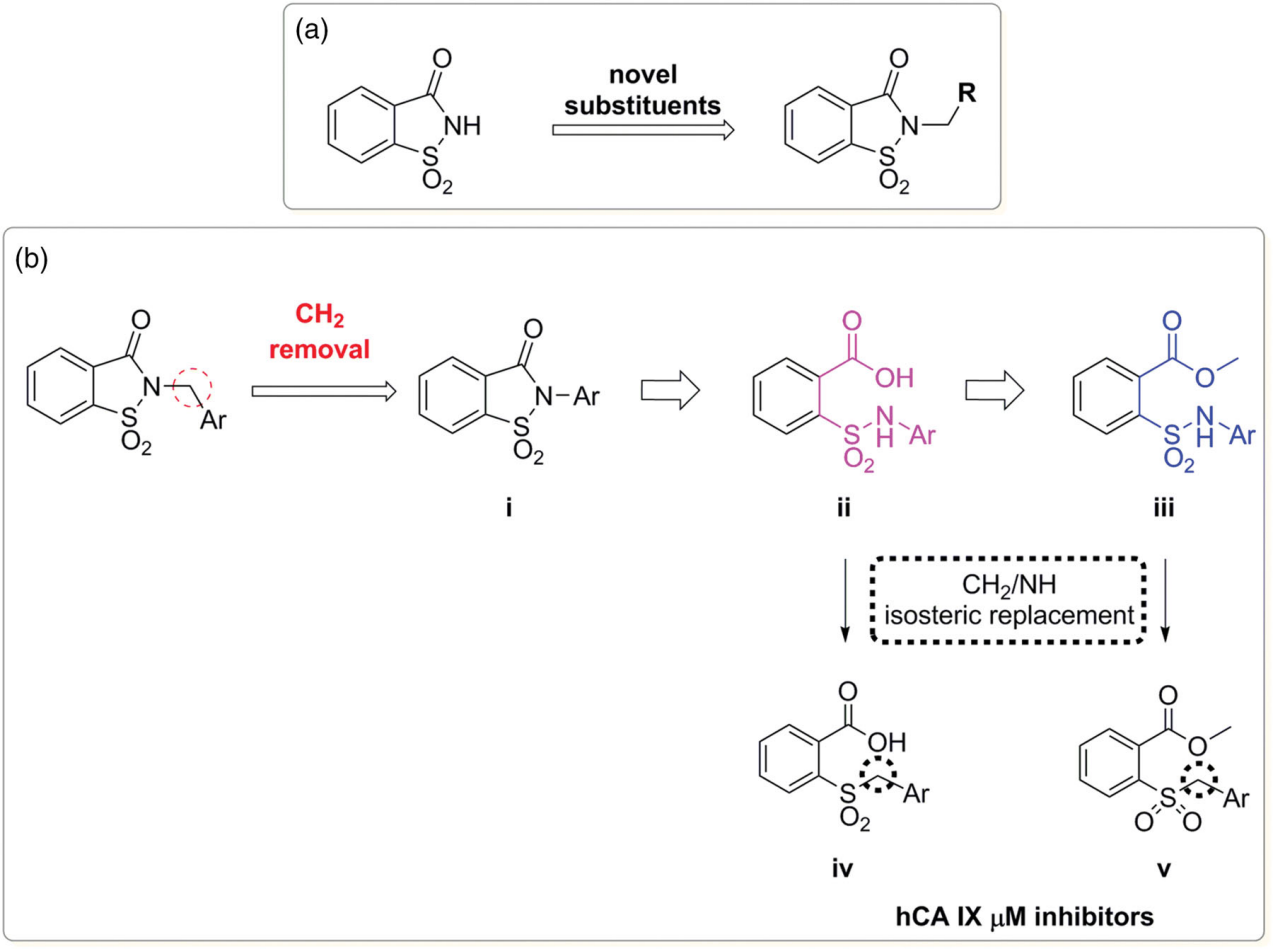
(a) The first design strategy proposed in this paper; (b) design and retrosynthetic approach for novel hCAs potential inhibitors.

Carbonic anhydrases (CAs, EC 4.2.1.1) are ubiquitous metalloenzymes which catalyse the reversible hydration of CO_2_ to bicarbonate and proton^1–4^. This quite simple reaction, that is very slow at physiological conditions, is involved in many fundamental processes such as respiration and transport of CO_2_/bicarbonate, pH and CO_2_ homeostasis, gluconeogenesis, lipogenesis and ureagenesis, bone resorption and many others^5–8^. Among the eight CA families discovered so far (α, β, γ, δ, ζ, η, θ, ι), α-CA are further grouped in 15 diverse human (h) isoforms, involved in a variety of physiological functions and pathological conditions^9–12^. In particular, the two transmembrane carbonic anhydrases IX and XII are also known as “tumor-related” isoforms, being overexpressed (mainly the isoform IX) in hypoxic tumors^13–18^. Unceasing efforts have been done and a lot of studies are ongoing aimed at discovering novel chemical libraries able to effectively inhibit hCA IX and XII^19–22^. Albeit classical CAs inhibitors take advantage of the presence of primary sulphonamide group^10^^,^^23–26^, in the last years a large number of compounds devoid of this chemical functionality have been discovered and evaluated as effective hCAs inhibitors^27–30^. Among the scaffolds frequently used for the development of “non classical” inhibitors of human carbonic anhydrases, saccharin and acesulfame have a great importance. Since the discovery of its inhibitory activity against carbonic anhydrase^31^, saccharin caught the attention as a valuable hit compound for the design of novel molecules acting against hCAs. In the past years several research groups, including ours, developed numerous hCAs inhibitors based on this promising scaffold^32–36^. The approaches used to modify this nucleus were based whether on the derivatization of the benzene moiety to retain the cyclic sulphonamide secondary group, or on its derivatization to obtain *N*-substituted saccharins. Our research group focused mostly on the second one, synthesising a large library of *N*-alkyl, *N*-benzyl or *N*-benzoylmethylene derivatives^37–39^. Pursuing our efforts in developing novel inhibitors of carbonic anhydrase, in this paper we report a large library of derivatives aimed to further explore the saccharin scaffold, taking advantage of a variety of design strategies. The first strategy used (Figure 1(a)) relies on the replacement of the saccharin nitrogen with unsaturated and branched alkyl chain, benzyl or benzoyl methylene moieties, as already proposed by our research group in the past years. Indeed, these substituents could represent a corollary for the robust structure-activity relationships already published.

The second one (Figure 1(b), **i**), is based on the removal of the methylene bridge included in the benzyl substituted derivatives. In this way we tried to evaluate, on one hand, the effects resulting from the loss of conformational freedom and, on the other, the consequences of the direct binding of the sulphonamide nitrogen atom to the phenyl ring. In fact, the removal of the methylene linker affects the electronic distribution, due to the onset of conjugation between the electron pair of nitrogen with the π-electrons of the phenyl ring, previously prevented by the presence of CH_2_ group.

Besides, the synthesis of these saccharin derivatives, consisting of a multistep approach, has been studied *ad hoc* to obtain in each step potential inhibitors for hCAs (Figure 1(b), **ii** and **iii**). Indeed, the acid intermediates (**ii**) possess two functional groups suitable for anchoring the zinc ion or the relative anchored water in the catalytic active site (-SO_2_NH- and COOH, respectively); moreover, they reflect the structure of the opened saccharins reported by Ivanova et al., that exhibited improved selectivity than their parent closed analogues against the tumour related isoforms of hCA (hCA IX and XII)^36^. These acid derivatives were obtained through the hydrolysis of the ester precursors that could be themselves potential inhibitors of the hCAs since they retain the secondary sulphonamide group. Moreover, considering the esterase activity of hCAs^40^, these molecules, if not active as esters, could undergo the enzyme-mediated hydrolysis in the active site acting as putative prodrugs of the acid compounds. The derivatives belonging to the sub-groups **ii** and **iii** of Figure 1(b) can also be considered the isosteres of a series of compounds recently reported by our group (Figure 1(b), **iv** and **v**)^41^. These inhibitors, even if devoid of any zinc binding group (ZBG), resulted active selectively against hCA IX in the µM range, prompting us to consider that the replacement of -CH_2_ by -NH could improve the activity against hCAs.

**Figure 2.**
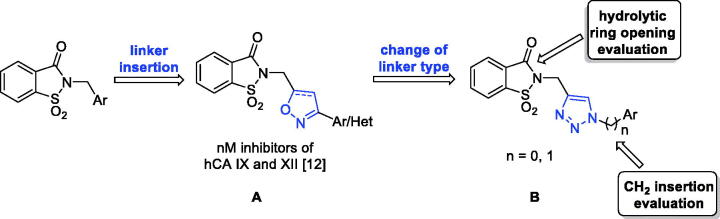
The third approach for the saccharin scaffold development.

The third design strategy was inspired from the findings achieved in our recent paper about a series of saccharin/isoxazole and saccharin/isoxazoline derivatives (Figure 2)^42^. These compounds were gifted with an isoxazole or isoxazoline linker which disconnected the methylene moiety from the phenyl ring, resulting in the strong affinity for hCA IX and hCA XII along with selectivity over hCA I and hCA II (Figure 2(A)). Thus, based on these good results, we proposed the replacement of the isoxazole “linker” with the triazole one, that resulted advantageous for some hCAs inhibitors (Figure 2(B))^43–46^. The triazole ring is, similar to the isoxazole one, an aromatic five-membered heterocyclic ring, even if it contains only the nitrogen heteroatom in spite of the oxygen/nitrogen ones of the isoxazole core. Moreover, it is able to establish stacking interactions in the lipophilic side of the active site as the isoxazole, and it is easy to insert through the well-known “click” azide-alkyne cycloaddition. For some of these derivatives the hydrolytic ring opening and/or the introduction of an additional methylene group between the N1 of the triazole and the phenyl ring bound to it, have also been evaluated. Indeed, the ring opening can lead to the same improvements discussed above as well as the insertion of the methylene group would increase the “flexibility” of the tail interrupting the electronic conjugation, too.

**Figure 3.**
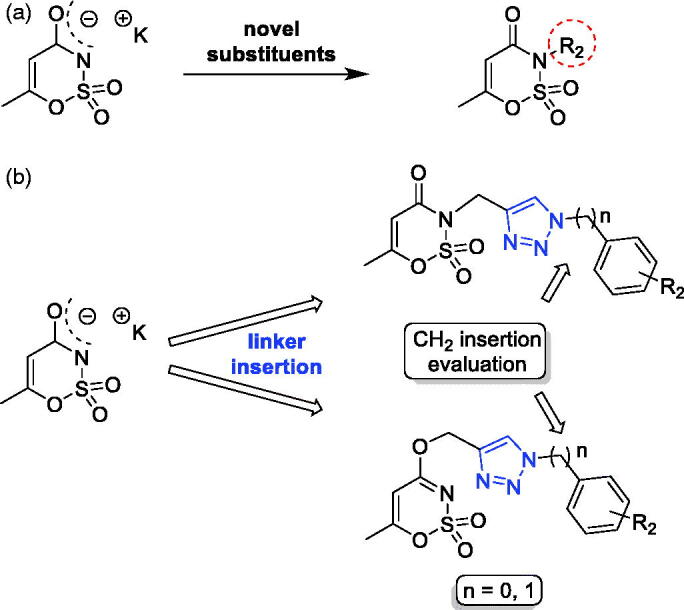
The first and third approaches applied on the acesulfame scaffold.

Similar to saccharin, the other artificial sweetener potassium acesulfame (Ace K) is a valid scaffold used for the development of hCA inhibitors (Figure 3). It has been largely explored both for its capability to inhibit carbonic anhydrase^47^, or after oxygen/nitrogen derivatization with different substituents (i.e. (un)saturated alkyl chains, (un)substituted benzyl or benzoylmethylene moieties)^37^^,^^48^. These latter exhibited good inhibitory activity against hCA IX and XII, although some of them retained residual activity against the off-targets hCA I/II. Taking advantage of the substitution approaches proposed for the saccharin-based compounds, we tried to translate the first and the third design strategies on the acesulfame scaffold (Figure 3(a,b)). By adjusting the synthesis conditions (see below), we were able to preferentially address the propargylation and then the triazole assembling, either at the oxygen or nitrogen of the acesulfame core to achieve *N-* and *O*-substituted analogues, respectively (Figure 3(b)). Even in this case, the insertion of an additional methylene group, disconnecting the phenyl group from the N1 of the triazole ring, was attempted. Seeing as how some compounds differing only for the nucleus (saccharin or acesulfame), it is also possible to evaluate the effects on the activity and selectivity of the molecules retaining the same tail but not the main core.

## 2. Experimental protocols

### 2.1. Chemistry

Unless otherwise specified, the reactions reported in this paper were performed under nitrogen atmosphere in washed and oven-dried glassware at room temperature. The solvents (anhydrous or not) and the reagents involved in the chemical and biological experimental protocols were used as supplied. If solvent mixtures are described, their ratio was expressed as *volume*:*volume*. The melting points of each compound were measured on a Stuart^®^ melting point apparatus SMP1 and the related temperature values reported in (uncorrected) °C. Proton (^1^H) and Carbon (^13 ^C) nuclear magnetic resonance spectra were recorded at 400.13 (or 300) and 101.03 (or 76) MHz respectively, on a Bruker spectrometer using the solvents (CDCl_3_, DMSO-*d*_6_, CD_3_OD and CD_3_CN) at room temperature. The final concentration of the samples was of ∼5 mg/mL for ^1^H-NMR acquisition and ∼25 mg/mL for the recording of the ^13^C-NMR ones. Chemical shifts are presented as *δ* units (parts per millions) using the solvent signal as the internal standard. ^1^H spectra are described as reported below: *δ*_H_ (spectrometer frequency, solvent): chemical shift/ppm (multiplicity, *J*-coupling constant(s), number of protons, assignment). ^13^C spectra are described as reported below: *δ*_C_ (spectrometer frequency, solvent): chemical shift/ppm (assignment). Coupling constants J are valued in Hertz (Hz) using these abbreviations to indicate the splitting: s – singlet; d – doublet; t – triplet; q – quadruplet; m – multiplet. If needed, it is also reported the abbreviation br – to indicate the broad shape of the specific peak.

Silica for column chromatography was purchased from Sigma-Aldrich (Milan, Italy) (high purity grade, pore size 60 Å, 230–400 mesh particle size). Reactions and purifications were checked by TLC performed on 0.2 mm thick silica gel-aluminium backed plates (60 F254, Merck) and their visualisation performed under ultra-violet irradiation (254 and 365 nm). If given, systematic compound names were generated by ChemBioDraw Ultra 12.0 in accordance to the IUPAC nomenclature. The HPLC analyses performed for purity evaluation were accomplished employing the Shimadzu *Prominence-i* LC-2030C 3D system endowed with autosampler, binary pump, column oven and a photodiode array detector (PDA). The separation was obtained through the use of the column Kinetex Biphenyl, 5 µm, 100 Å (Phenomenex, Bologna, Italy) at a constant flow of 1.0 ml min^−1^, employing an isocratic elution. For the acesulfame derivatives (**50**–**60**), closed saccharins (**1**–**4**, **29**–**46**) as well as the opened ones containing the ester functional group (**5**–**16**), were employed the two eluents water (solvent A) and acetonitrile (solvent B) mixed in the constant ratio of 50:50 (*v*:*v*). For the opened saccharins containing the carboxylic acid group (**17**–**28**, **47**–**49**) the two solvents were added with 0.1% of trifluoroacetic acid (TFA) maintaining the ratio 50:50 (*v*:*v*); all the runs were completed in 10 min. All the analyte solutions were prepared in acetonitrile (acetonitrile plus 0.1% of TFA for acidic derivatives) at the concentration between 0.5–2 mg mL^−1^, and 5 µL were directly injected for the HPLC analysis. All compounds reported were ≥96% HPLC pure. The solvents used in the HPLC analysis were acetonitrile, purchased from Carlo Erba Reagents and mQ water 18 MΩ cm, obtained from Millipore’s Direct-Q3 system. All the synthetic procedures and characterisation data for each compound were reported as Supplementary data.

### 2.2. *CA* inhibition screening assay

An Applied Photophysics stopped-flow instrument has been used for assaying the CA-catalyzed CO_2_ hydration activity^49^. Phenol red, at a concentration of 0.2 mM, has been used as an indicator, working at the maximum absorbance of 557 nm with 20 mM Hepes, 4–(2-hydroxyethyl)-1-piperazineethanesulfonic acid, (pH 7.5 for α-CAs) as buffer and 20 mM Na_2_SO_4_ (for maintaining constant the ionic strength, but without inhibiting the enzyme). The initial rates of the CA-catalysed CO_2_ hydration reaction were followed for a period of 10−100 s. The CO_2_ concentrations ranged from 1.7 to 17 mM for the determination of the kinetic parameters and inhibition constants. For each inhibitor, at least six traces of the initial 5−10% of the reaction have been used for determining the initial velocity. The uncatalyzed rates were determined in the same manner and subtracted from the total observed ones. Stock solutions of each inhibitor (0.1 mM) were prepared in distilled − deionized water and dilutions up to 0.01 nM were done thereafter with distilled − deionized water. Inhibitor and enzyme solutions were preincubated together for 15 min at room temperature prior to assay to allow for the formation of the E−I complex. The inhibition constants were obtained by nonlinear least-squares methods using the Cheng-Prusoff equation and represent the mean from at least three different determinations. Errors were in the range of ± 5−10% of the reported *K*_I_ values. Human CA isoforms were recombinant enzymes obtained in-house as reported earlier^50–52^. The enzyme concentrations in the assay system were as follows: hCA I, 13.2 nM; hCA II, 8.4 nM; hCA IX, 7.9 nM; hCA XII, 15.2 nM.

### 2.3. Molecular modelling

The crystal structure of CA IX (pdb 5DVX)^53^ and CA XII (pdb 1JCZ)^54^ were prepared using the Protein Preparation Wizard tool implemented in Maestro-Schrödinger suite, assigning bond orders, adding hydrogens, deleting water molecules, and optimising H-bonding networks^44^. Energy minimisation protocol with a root mean square deviation (RMSD) value of 0.30 was applied using an Optimised Potentials for Liquid Simulation (OPLS3e) force field. 3 D ligand structures were prepared by Maestro^55^and evaluated for their ionisation states at pH 7.4 ± 0.5 with Epik^55^. OPLS3e force field in Macromodel^55^was used for energy minimisation for a maximum number of 2500 conjugate gradient iteration and setting a convergence criterion of 0.05 kcal mol^−1 ^Å^−1^. The centroid of the zinc-bound water molecule was selected as grid centre and Glide used with default settings. Ligands were docked with the standard precision mode (SP) of Glide^55^and the best 5 poses of each molecule retained as output. The best pose for each compound to CA IX and CA XII, evaluated in terms of anchorage, hydrogen bond interactions and hydrophobic contacts, was submitted to a MD simulation using Desmond^48^ and the OPL3e force field. Specifically, the system was solvated in an orthorhombic box using TIP4PEW water molecules, extended 15 Å away from any protein atom. It was neutralised adding chlorine and sodium ions. The simulation protocol included a starting relaxation step followed by a final production phase of 100 ns. In particular, the relaxation step comprised the following: (a) a stage of 100 ps at 10 K retaining the harmonic restraints on the solute heavy atoms (force constant of 50.0 kcal mol^−1 ^Å^−2^) using the NPT ensemble with Brownian dynamics; (b) a stage of 12 ps at 10 K with harmonic restraints on the solute heavy atoms (force constant of 50.0 kcal mol^−1 ^Å^−2^), using the NVT ensemble and Berendsen thermostat; (c) a stage of 12 ps at 10 K and 1 atm, retaining the harmonic restraints and using the NPT ensemble and Berendsen thermostat and barostat; (f) a stage of 12 ps at 300 K and 1 atm, retaining the harmonic restraints and using the NPT ensemble and Berendsen thermostat and barostat; (g) a final 24 ps stage at 300 K and 1 atm without harmonic restraints, using the NPT Berendsen thermostat and barostat. The final production phase of MD was run using a canonical NPT Berendsen ensemble at temperature 300 K. During the MD simulation, a time step of 2 fs was used while constraining the bond lengths of hydrogen atoms with the M-SHAKE algorithm. The atomic coordinates of the system were saved every 100 ps along the MD trajectory. Protein and ligand RMSD values, ligand torsions evolution and occupancy of intermolecular hydrogen bonds and hydrophobic contacts were computed along the production phase of the MD simulation with the Simulation Interaction Diagram tools implemented in Maestro.

## 3. Results and discussion

### 3.1. Chemistry

**Scheme 1.**
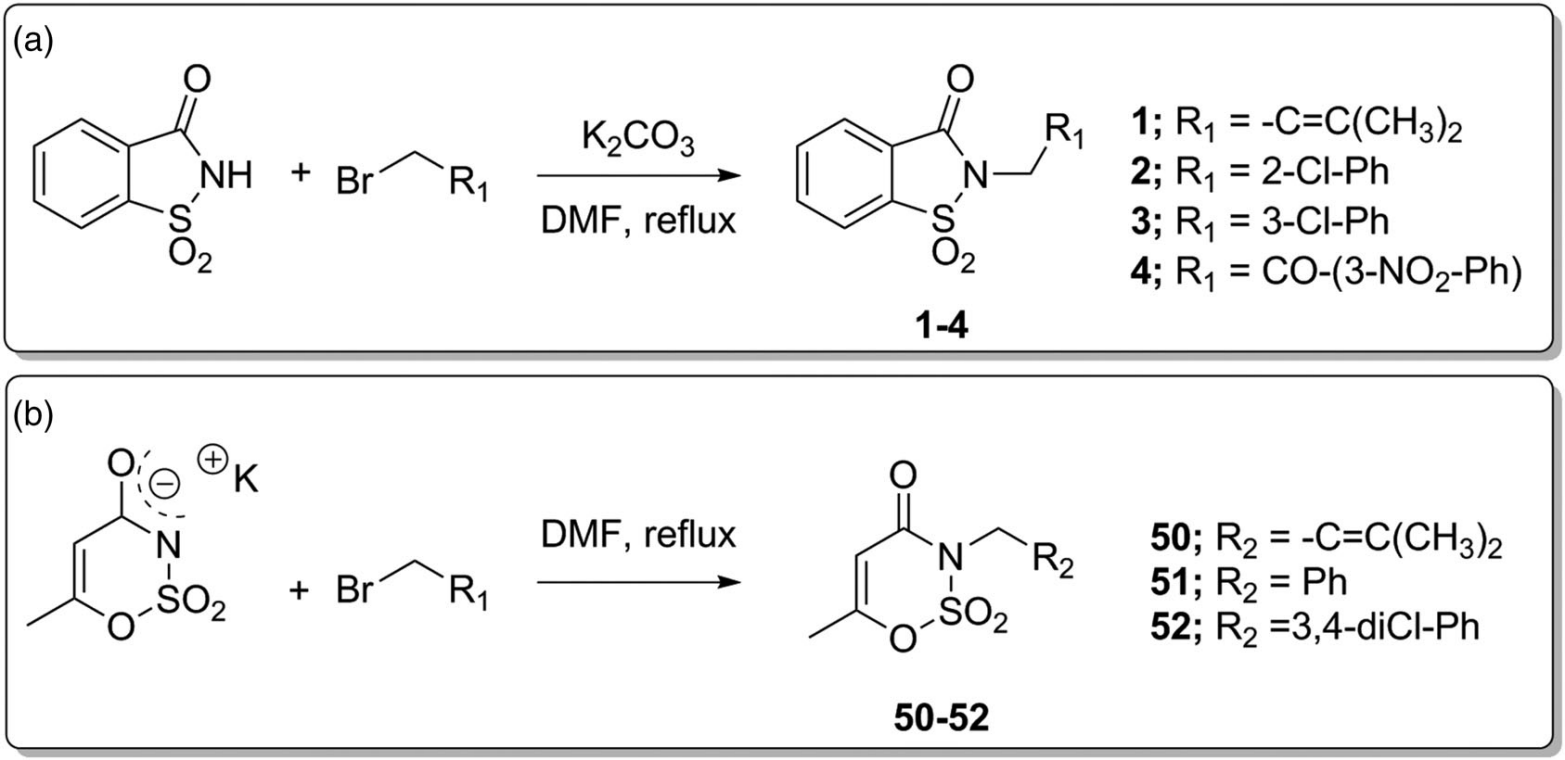
Synthetic procedure for derivatives **1–4** and **50–52**.

Derivatives **1–4** and **50–52** were synthesised according to our previously published protocols^37^, consisting in the nucleophilic substitution reaction between saccharin or potassium acesulfame with the proper electrophile at 80 °C for 24–48 h as appropriate (Scheme 1(a,b)). These reactions were performed in DMF, in the presence of freshly ground anhydrous potassium carbonate (K_2_CO_3_) for saccharin derivatives (Scheme 1(a)); Ace K did not require the adding of further base to activate the nucleophile site, being employed as potassium salt (Scheme 1(b)).

**Scheme 2.**
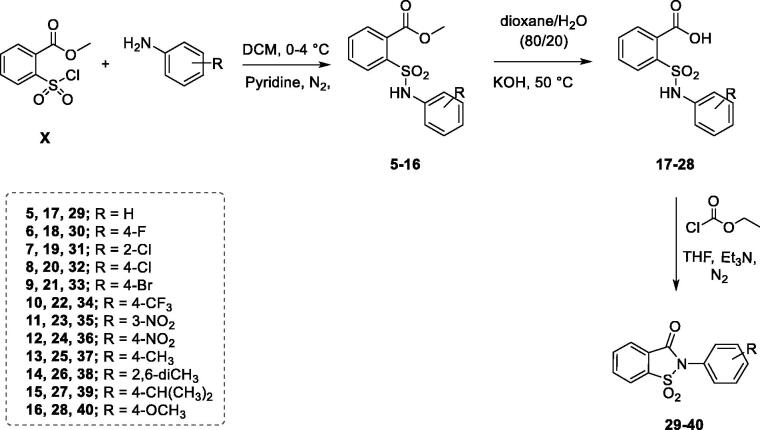
Synthetic procedure for derivatives **5–16**, **17–28** and **29–40**.

Derivatives **29–40** were synthesised using the multistep procedure reported in Scheme 2; by selecting this synthetic pathway we were able to obtain also the derivatives **5–28**, that are synthetic precursors and opened analogues of the compounds **29**–**40**.

The first step, leading to the ester intermediates **5–16**, involved the reaction between the methyl 2-(chlorosulfonyl)benzoate (Scheme 2, **X**) and the proper aniline in DCM at 0 °C, in the presence of pyridine which worked both as base and catalyst. In this regard, the first attempted synthetic strategy provided for one single addition of the aniline to the solution of methyl 2-(chlorosulfonyl)benzoate in pyridine. Since the yield had not been satisfying, different methods were attempted and the best one resulted in the portioned addition of the methyl 2-(chlorosulfonyl)benzoate to the solution of aniline and pyridine over a period of 45 min.

The ester derivatives (**5–16**) were hydrolysed to the corresponding acids (**17–28**) employing potassium hydroxide dissolved in a mixture of water/1,4-dioxane in the ratio 80/20 (*v*/*v*). The isothiazolone ring closure was achieved taking advantage of the mixed anhydride method, used to activate carboxylic acid group^56^. The synthesis was performed in tetrahydrofuran using ethyl chloroformate as activating agent in the presence of triethylamine, affording the saccharin-based compounds **29–40**.

**Scheme 3.**
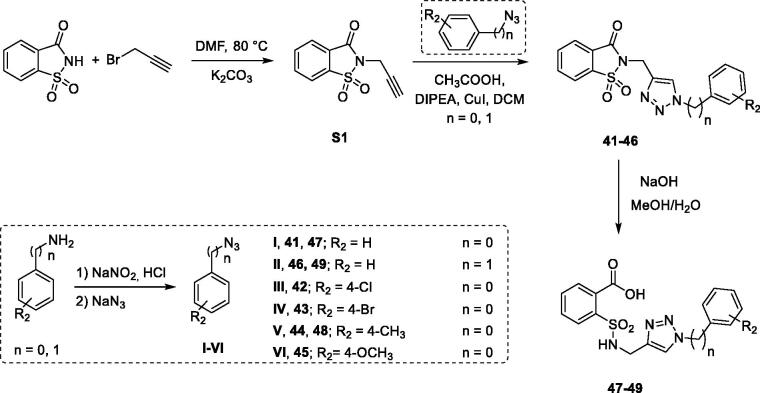
Synthetic procedure for derivatives **41–49**.

For the synthesis of the molecules **41**–**46** (and the opened analogues **47–49**) we exploited a multistep approach, too (Scheme 3). Indeed, we performed at first the preparation of the intermediates consisting of propargylated saccharin and the proper phenyl or benzyl azide, which then reacted in the final “click” reaction giving the final compounds in good yield.

The propargylated saccharin (Scheme 3, **S1**) was synthesised accordingly to our published procedure that involves the nucleophilic substitution between saccharin and propargyl bromide in DMF, at 80 °C for 48 h in the presence of potassium carbonate^39^. The azide intermediates were synthesised through the nucleophilic aromatic substitution between the diazonium salt of the suitable aniline or benzylamine prepared *in situ* and the sodium azide. Then, the so obtained intermediates were “clicked” in the final reaction in the presence of a catalytic amount of copper iodide, DIPEA and acetic acid following the procedure reported by Shao and colleagues^57^. Compounds **47–49** were attained through the hydrolysis of their closed analogues using NaOH dissolved in a mixture of MeOH/H_2_O in the ratio 50/50 (*v*/*v*).

**Scheme 4.**
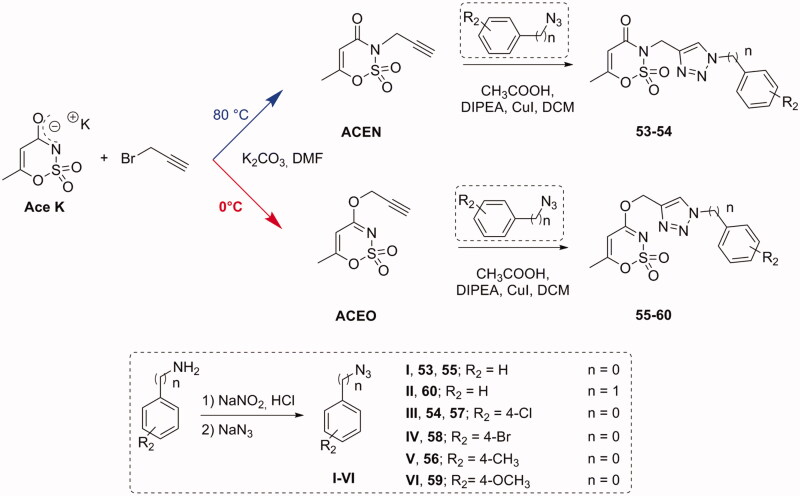
Synthetic procedure for derivatives **53**–**60**.

The synthesis of the acesulfame derivatives endowed with the triazole “linker” moiety was performed in a similar manner (Scheme 4). However, by finely regulating the synthetic conditions of the Ace K propargylation step, we specifically addressed the derivatization on the nitrogen or oxygen to obtain *N-* (**53–54**) or *O-*substituted (**55–60**) acesulfame analogues, respectively. In particular, we observed as the *N*-propargylated derivative was the main product by conducting the synthesis at 80 °C (Scheme 4, **ACEN**); on the contrary, performing the reaction at lower temperature (0 °C), we attained the *O*-propargylated one as primary product (Scheme 4, **ACEO**). These intermediates were reacted along with the phenyl/benzyl azides, seen before in the final “click” reaction, with the same conditions adopted for saccharin derivatives. All the compounds have been characterised by melting point, TLC parameters, ^1^H and ^13 ^C NMR. Their purity was evaluated through HPLC analysis, employing two different methods: the first used for acesulfame derivatives (**50**–**60**), closed saccharins (**1–4**, **29–46**) as well as opened saccharins containing methyl ester group (**5**–**16**); the second one used for opened saccharin derivatives with carboxylic acid group (**17–28**, **47–49**). In particular, the compounds were analysed using isocratic elution with a binary mobile phase composed by water and acetonitrile (added of 0.1% with trifluoroacetic acid for the acidic derivatives) and were ≥96% HPLC pure (See Supporting information).

### 3.2. Inhibition of hCA I, II, IX, and XII

The data reported in Tables 1–2 show how the inhibitory activity of the compounds **1–60** is strictly influenced by the substituent group/linker bound to the saccharin and acesulfame cores. In general, these derivatives were less effective than the previously reported ones by our research group^37–39^^,^^48^; however, some of them retained nanomolar activity and selectivity against hCA IX and XII.

**Table 1.** Inhibition data of selected human CA isoforms (hCA I, II, IX and XII) with saccharin-based derivatives **1–49** reported here and the standard sulphonamide inhibitor acetazolamide (**AAZ**) by a stopped flow CO_2_ hydrase assay^49^.

	Structure	*K*_i_ (µM)			
		hCA I	hCA II	hCA IX	hCA XII
1		>1000	>1000	0.39	0.23
2		>1000	>1000	0.30	0.22
3		>1000	>1000	0.10	1.3
4		>1000	>1000	>1000	0.41
5		>1000	>1000	59.6	>1000
6		>1000	>1000	261.8	>1000
7		>1000	>1000	427.5	>1000
8		>1000	>1000	955.3	>1000
9		>1000	688.7	6.7	>1000
10		>1000	>1000	562.6	>1000
11		>1000	>1000	>1000	>1000
12		>1000	>1000	62.8	>1000
13		>1000	>1000	68.2	>1000
14		>1000	>1000	39.7	>1000
15		>1000	>1000	57.6	>1000
16		>1000	>1000	93.3	>1000
17		>1000	>1000	46.5	>1000
18		>1000	>1000	45.8	>1000
19		>1000	>1000	>1000	>1000
20		>1000	>1000	>1000	>1000
21		>1000	>1000	72.8	>1000
22		>1000	>1000	84.8	>1000
23		>1000	>1000	>1000	>1000
24		>1000	66.9	0.24	>1000
25		>1000	>1000	>1000	>1000
26		>1000	>1000	>1000	>1000
27		>1000	>1000	>1000	>1000
28		>1000	>1000	>1000	>1000
29		>1000	>1000	>1000	>1000
30		>1000	>1000	>1000	>1000
31		>1000	477.8	640.0	>1000
32		>1000	>1000	>1000	>1000
33		>1000	>1000	869.1	>1000
34		>1000	>1000	>1000	>1000
35		>1000	>1000	>1000	>1000
36		>1000	>1000	443.5	>1000
37		>1000	>1000	>1000	>1000
38		>1000	>1000	>1000	>1000
39		>1000	>1000	>1000	>1000
40		>1000	>1000	>1000	>1000
41		>1000	>1000	>1000	>1000
42		>1000	80.8	758.6	>1000
43		>1000	76.4	537.6	>1000
44		>1000	>1000	669.6	>1000
45		>1000	>1000	666.1	>1000
46		>100	>100	20.9	7.4
47		>1000	>1000	926.9	>1000
48		>1000	>1000	>1000	>1000
49		>100	>100	1.9	4.5
AAZ		250	12	25	5.7

**Table 2.** Inhibition data of selected human CA isoforms (hCA I, II, IX and XII) with acesulfame-based derivatives **50–60** reported here and the standard sulphonamide inhibitor acetazolamide (**AAZ**) by a stopped flow CO_2_ hydrase assay^49^.

	Structure	*K*_i_ (µM)^a^			
		hCA I	hCA II	hCA IX	hCA XII
50		>1000	>1000	0.33	0.24
51		>1000	>1000	2.7	0.27
52		>1000	>1000	0.47	2.0
53		>1000	>1000	>1000	>1000
54		>1000	>1000	>1000	>1000
55		>1000	>1000	>1000	>1000
56		>1000	>1000	>1000	>1000
57		>1000	>1000	>1000	>1000
58		>1000	>1000	>1000	>1000
59		>1000	>1000	>1000	>1000
60		>100	>100	1.1	>100
AAZ		250	12	25	5.7

^a^Mean from 3 different assay, by a stopped flow technique (errors were in the range of ± 5–10% of the reported values).

### 3.3. Saccharin-based derivatives

The inhibitory activity data of saccharin derivatives are reported in Table 1. Compound **1**, endowed with the unsaturated/branched prenyl group (3-methylbut-2-en-1-yl) bound at the nitrogen atom of saccharin core, exhibited exclusive inhibitory activity against the tumour related isoforms hCA IX and XII (*K*_I_ hCA I/II > 1000 µM). The two isoforms were inhibited in the nanomolar range, with a little preference for the isoform XII (*K*_I_ hCA IX = 390 nM; *K*_I_ hCA XII = 230 nM). The derivatives **2** and **3**, endowed with 2- and 3-chlorobenzyl group respectively, as well as **4**, bearing the 3-nitrobenzoyl methylene moiety, were ineffective against the off-targets hCA I and II. Compound **4** did not exhibited activity against hCA IX, too (*K*_I_ hCA IX > 1000 µM), while it inhibited hCA XII in the high nanomolar range (*K*_I_ hCA XII = 410 nM). Interestingly, the removal of the carbonyl group, turning from benzoyl methylene moiety to the benzyl one (**2** and **3**), improved the activity against hCA IX. In particular, compound **2** bearing the chloro atom in the *meta* position of the phenyl ring was three times more potent than the *ortho*-chloro analogue **3** (**2**, *K*_I_ hCA IX = 100 nM; **3**, *K*_I_ hCA IX = 300 nM). Instead, an opposite inhibitory profile was observed against hCA XII, since **3** displayed better activity than **2** (**2**, *K*_I_ hCA XII = 220 nM; **3**, *K*_I_ hCA XII = 1300 nM).

With the aim to investigate the importance of the methylene “linker” included in the benzyl group, we attempted the removal of this moiety leading to a series of compounds in the opened (**5–28**) as well as in the closed (**29–40**) form. In this regard, three different “sub-series” were evaluated. The first (**5–16**) is based on the opened saccharin core containing the methyl ester group in spite of the carboxylic acid one that is present in the second sub-group (**17–28**). All these derivatives have on the carbon atom neighbouring to the one bearing the ester/carboxylic acid functional groups, a secondary sulphonamide whose the nitrogen atom binds to a (un)substituted phenyl rings. The third sub-group includes closed saccharin derivatives substituted at the nitrogen with the same (un)substituted phenyl groups of the opened analogues (**29–40**).

All these compounds were less effective than their “counterpart” containing the methylene bridge. With regard to the derivatives in the opened form, only compound **9**, bearing the 4-bromophenyl ring, exhibited a weak affinity for hCA IX although in the micromolar range (*K*_I_ hCA XII = 6.7 µM). All the other methyl ester opened saccharins were almost or completely ineffective against all the tested isoforms (see Table 1). Compounds **17–28**, bearing the carboxylic acidic group in spite of the methyl ester one, exhibited an inhibitory profile similar to that observed for compounds **5–16**. However, compound **24**, containing the *p-*nitrophenyl group bound at the nitrogen of sulphonamides, displayed an effective inhibition towards hCA IX (*K*_I_ hCA IX = 240 nM), retaining a residual activity against hCA II, although in the high micromolar range (*K*_I_ hCA II = 66.9 µM). The ring closure, leading to the derivatives **29–40**, was unproductive because of the absence of inhibitory activity against all the hCA isoforms evaluated. Indeed, regardless of the chemical properties of the nitrogen substituents, no activity better than 443 µM (**36**) was observed against hCA IX; furthermore, all these compounds were inactive against hCA I and XII, and only compound **31** exhibited an extremely weak activity against hCA II (**31**, *K*_I_ hCA II = 477.8 µM). Thus, in the light of these results it is undisputable that the methylene linker, removed to obtain compounds **5–40**, is fundamental for the activity. The reason of this structural requirement could be related to the degrees of freedom that this linker confers to the phenyl moiety, giving it the opportunity to “search” for points of interactions inside the active site. Moreover, it cannot be excluded that in compounds **5–40** the presence of a direct bond between the nitrogen and the phenyl ring, may change the electronic distribution, affecting negatively the placement inside the active site, making also the secondary sulphonamide of the opened forms (**5–28**) ineffective in rising the activity.

Inspired by these observations and considering the good results gained by the insertion of the isoxazole/isoxazoline heterocyclic linker in our previous work^42^, we tried to evaluate the effects caused by the insertion of triazole core between the methylene group and the phenyl ring, substituting the isoxazole one (**41–49**). This replacement did not elicit the desired results, because most of these analogues were devoid, or almost, of inhibitory activity against the evaluated isoforms. Compound **41**, the simplest one of these group of derivatives, did not exhibit inhibition towards all the tested isoforms (*K*_I_ hCA I/II/IX/XII > 1000 µM). The introduction of substituents, placed at the *para* position of the phenyl ring bound to the triazole N1, affected only slightly the activity against hCA II and IX, whereas did not ameliorate the affinity towards hCA XII (see Table 1). The only change that dramatically improved the inhibitory activity was the insertion of a second methylene group that “disconnected” the triazole N1 from the phenyl ring bound it. As one can see for compound **46**, that is the analogue of **41** containing that methylene moiety, the increment of inhibitory activity was remarkable, turning from an absence of affinity against the tumour related isoforms (*K*_I_ hCA IX/XII > 1000 µM) to a micromolar inhibitory activity against the two target isoforms (*K*_I_ hCA IX = 20.9 µM; *K*_I_ hCA XII = 7.4 µM). A comparable effect, coming from the addition of the methylene group, was observed for **47** and **49**, which are the hydrolytically obtained opened analogues of **41** and **46**, respectively. In fact, compound **47**, devoid of the “extra” methylene group, was ineffective against all the tested isoforms (as well as the compound **48** differing from the former for *p-*methyl substitution on the phenyl ring); on the contrary, derivative **49**, endowed with the additional methylene group, exhibited low micromolar inhibitory activity exclusively against the two cancer related isoforms, even better than the closed analogue **46** (**46**, *K*_I_ hCA IX = 20.9 µM; *K*_I_ hCA IX = 7.4 µM; **49**, *K*_I_ hCA IX = 1.9 µM; *K*_I_ hCA IX = 4.5 µM). Therefore, though the presence of the triazole ring in spite of the isoxazole/isoxazoline ones did not positively affect the inhibitory activity, some information can be examined. In particular, we observed that similarly to what detected for saccharins **5–40**, the presence of the methylene group between the N1 of triazole ring and the phenyl group improves the activity (see the couples **41/46** and **47/49**) as well as the hydrolytic ring opening seems to increase the inhibitory activity against the tumour related isoforms hCA IX and XII.

### 3.4. Acesulfame-based derivatives

The inhibitory activity data of acesulfame derivatives are reported in Table 2. Compounds **50–52** are acesulfame derivatives substituted at the nitrogen atom with (un)substituted benzyl groups (**51** and **52**) or with the prenyl one (**50**). These molecules exhibited selectivity against hCA IX and XII, missing affinity towards the *off-target* isoforms (**50–52**, *K*_I_ hCA I/II > 1000 µM). Among the three derivatives, **50** exhibited the better inhibition profile against hCA IX and XII (*K*_I_ hCA IX = 330 nM; *K*_I_ hCA XII = 240 nM). Compound **51**, bearing the unsubstituted benzyl group, inhibited efficiently the isoform XII (*K*_I_ hCA XII = 270 nM), and in a lesser extent the isoform IX (*K*_I_ hCA IX = 2.7 µM). On the contrary, **52**, containing the 3,4-dichloro benzyl group, exhibited preference for hCA IX, which was inhibited in the nanomolar range (*K*_I_ hCA IX = 2.7 µM), instead of hCA XII, that was affected only at the micromolar range (*K*_I_ hCA XII = 2.0 µM). In order to evaluate the effects provoked by the insertion of the triazole linker on the acesulfame scaffolds, compounds **53–54** (*N*-substituted) and **55–60** (*O-*substituted) were synthetised and assessed against the hCA isoforms seen before. These molecules missing appreciable activity against these enzymes, as previously observed with saccharin derivatives. In fact, satisfactory inhibitory activity was not found regardless of the position of the substituents on the acesulfame core (*O-* or *N-*substituted) and of the groups bound at the phenyl ring (see Table 1). However, as already observed for saccharin derivatives, the insertion of an additional methylene group between the phenyl ring and the triazole N1 led to the onset of a low micromolar activity against hCA IX for compound **60**, the only active of this series (*K*_I_ hCA IX = 1.1 µM). In the light of the above, the data obtained from the acesulfame derivatives confirm those observed for saccharins, namely that the insertion of additional methylene group, between the triazole N1 and the phenyl ring, positively affected their inhibitory activity.

## 4. Molecular modelling studies

A subset of CAIs, namely compounds **2**, **46**, **49** and **51**, endowed with the best CA IX and XII inhibitory profiles, was selected to study the interaction mechanism driving the inhibition profiles reported in Tables 1 and 2. A computational protocol, consisting of joint docking procedure and MD simulations, was used to investigate the compounds binding mode within CAs IX and XII (see Experimental Section), considering the derivatives as CAIs anchoring to zinc-bound water molecule basing on a number of considerations: (i) zinc-binder CAI chemotypes, such as primary sulphonamides, sulfamates and sulfamides, mono- and dithiocarbamates, or hydroxamates, mainly act in the deprotonated form, as anions, straightly coordinating the Zn(II) ion from the enzyme active site^20^; (ii) saccharin **1–4**, **29–46** and acesulfame **50**–**60** derivatives bearing a tertiary sulphonamide moiety cannot interact with the targets in the deprotonated form; (iii) although the deprotonation of the secondary sulphonamide group of hydrolysed saccharin derivatives **5–28** and **47–49** is possible, its internal position in the molecules does not allow it to coordinate the Zn ion due to the steric hindrance of the groups surrounding the negatively charged sulphonamide; (iv) crystallographic evidence showed that deprotonated sulphonate moieties, which possess similar features as the SO_2_ group of the here reported saccharin and acesulfame compounds, drive CA inhibition by anchoring to the zinc-bound water molecule/hydroxide ion (not by coordination to the zinc ion)^20^. This led us to consider in our *in silico* studies the nucleophile mediated anchoring mechanism for derivatives **2**, **46** and **51**. Likewise, crystallographic evidence showed that carboxylic acids as **49**, except for few cases, act as CAIs anchoring to the zinc-bound nucleophile^20^.

**Figure 4.**
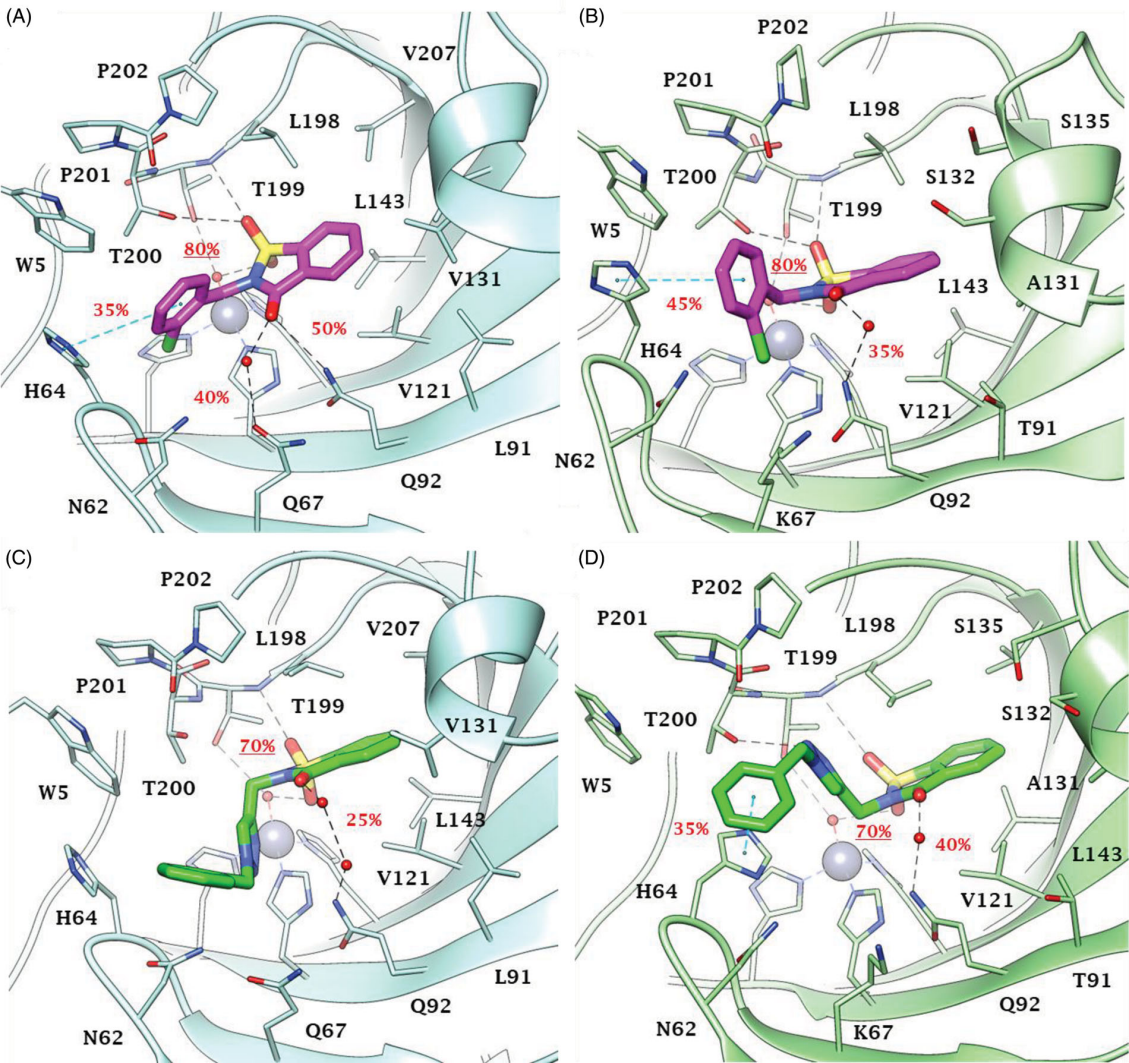
Predicted binding mode of compounds **2** and **46** into (A, C) CA IX and (B, D) CA XII active site. H-bonds and π-π stackings are represented as black and blue dashed lines, respectively. Dashed bonds occupancy over the MD simulation is indicated as percentage, among which underlined is the occupancy of the anchorage to the zinc-bound water. Water molecules are shown as red spheres. Amino acids are labelled with one letter symbols: A, Ala; H, His; K, Lys; L, Leu; N, Asn; P, Pro; Q, Gln; S, Ser; T, Thr; V, Val; W, Trp.

**Figure 5.**
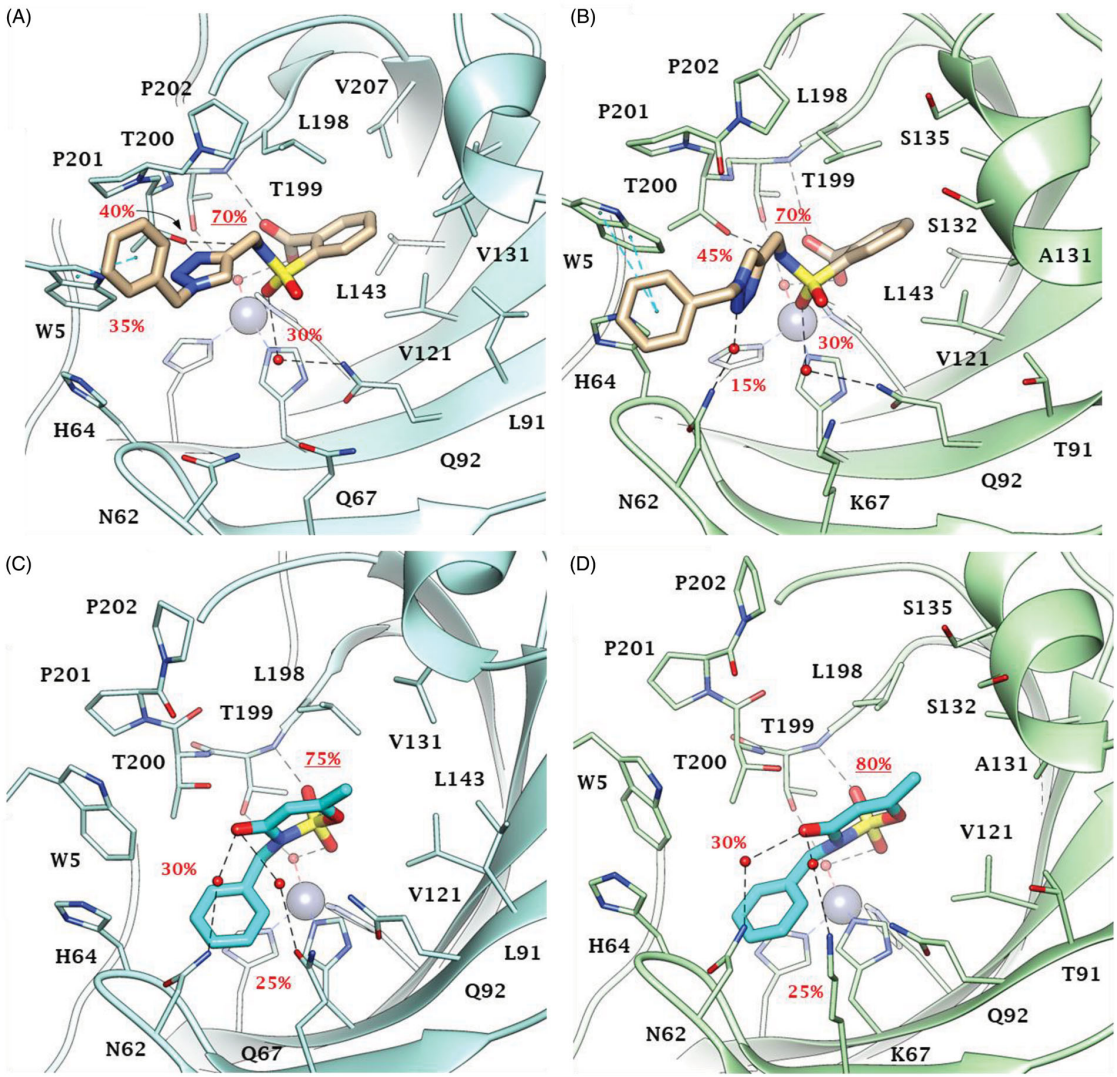
Predicted binding mode of compounds **49** and **51** into (A, C) CA IX and (B, D) CA XII active site. H-bonds and π-π stackings are represented as black and blue dashed lines, respectively. Dashed bonds occupancy over the MD simulation is indicated as percentage, among which underlined is the occupancy of the anchorage to the zinc-bound water. Water molecules are shown as red spheres. Amino acids are labelled with one letter symbols: A, Ala; H, His; K, Lys; L, Leu; N, Asn; P, Pro; Q, Gln; S, Ser; T, Thr; V, Val; W, Trp.

Thus, dockings were performed including the Zn-bound water molecule in the target and the reliability of poses for derivatives **2**, **46**, **49** and **51** within CA IX and XII active sites (Figures 4 and 5) assessed with MD simulations. The MD trajectories showed the stability of the anchorage to the zinc-bound water molecule that persists for >70–80% of the simulation time; moreover, the ligands maintain constant hydrophobic contacts with different portions of the active sites. In both CA IX and XII saccharin derivatives **2** and **46** are H-bond anchored by one S=O group to the metal-coordinated water that is, in turn, H-bonded to side chain hydroxyl group of Thr199 (Figures 4 and 5). The other S=O group of both ligands accepts one H-bond by Thr199 backbone NH, but just **2** also engages in hydrogen bonding with the side chain hydroxyl group of Thr200 (O⋯H-O Tyr200). Of note, this H-bonds network persists for most MD simulations mainly contributing to the pose stability. Moreover, the C=O group of the ligands engages direct or water bridged H-bonds with Gln67 and Gln92 which fluctuate over the MD computations. The N-benzyl group of derivative **2** accommodates in the pocket lined by Trp5, His64 and Asn62 forming VdW contacts and π-π interactions with the aromatic residues. This cleft is only partially occupied by the 1-benzyl-1,2,3-triazol-4-yl *N*-pendant of derivative **46** over the MD course making the docking poses less stable, and thereby providing a plausible explanation of the worst CA IX and XII inhibitory profile of **46** compared to **2** (Figures 4 and 5). The interaction mode of **2** and **46** within both CA IX and XII can explain the lower up to absent CAs inhibition observed for the *N*-phenyl saccharin derivatives **29**–**40** and 1-phenyl-1,2,3-triazole **41–45**.

In fact, the lack of a methylene spacer between the heterocycle and the aromatic pendant (as in **29**–**40**) or between the triazole and the outer aromatic ring (as in **41–45**) hampers a suitable ligand/target complementarity interaction and stable accommodation of the pendant in the pocket formed by Trp5, His64 and Asn62 (Supplementary Figure S1).

Both in CA IX and XII active sites, the hydrolysed saccharin **49** anchors to the zinc-bound water molecule by the carboxylate group, that also engages one H-bond with Thr199 backbone NH (Figure 5(A,B)). Its negatively charged sulphonamide group SO_2_NH^–^ takes part to a network of H-bonds involving multiple residues of the binding cavity: Gln92 by a fluctuating water-bridged H-bond (N-H⋯(H)OH⋯Gln92) and Tyr200 (N-H⋯O-Thr200). In CA IX, the hydroxyl side chain of Thr200 is also in H-bond contact with the ligand triazole N3 atom, and π-π contacts are formed by the indole moiety of Trp5 and the outer phenyl ring of **49**. In contrast, the placing of the 1-benzyl-1,2,3-triazol-4-yl *N*-pendant within the pocket formed by Trp5, His64 and Asn62 in CA XII is less stable over the MD course with respect to CA IX, as well as the water bridged H-bond formed by the triazole NH and Asn62 and the π-π interactions (Figure 5(B)). Again, the absence of a methylene spacer between the sulphonamide and the aromatic *N*-pendant, as in the methyl esters **5–16** and carboxylic acids **17–28**, translates into a lower CA IX and XII inhibition activity of these derivatives when compared to **49**. However, the drop of efficacy was lower than that observed with saccharin analogues most likely because of the greater conformational flexibility and adaptation capability within the active site of the hydrolysed ligands than cyclized compounds. Surprisingly, while a total loss of CA XII inhibition activity was observed for **5**–**28**, their medium micromolar profile against CA IX is likely to be related to the hydrophobic features of the CA IX active site and to the larger size of its cleft able to host the benzoate group when the triazole ring brings both a phenyl and benzyl moiety. As already observed for **47** and **48** the absence of the methylene spacer between the triazole and the outer aromatic ring, produced a whole loss of action against CAs.

Likewise, in both CA IX and XII, acesulfame **51** anchors to the zinc-bound water molecule through the sulfimide S=O (Figures 5(C,D)) and the anchorage strengthened by another H-bond occurring between the other S=O group and Thr199 backbone NH. The carbonyl group of **51** is involved in a network of water-bridged H-bond with Asn62, Asn/Lys67 (CA IX/XII) and Gln92. Interestingly, the *N*-benzyl moiety stably accommodates (>65% MD) within the pocket formed by Trp5, Tyr7, His64, Asn62 and His96 engaging VdW contacts. On the basis of the depicted binding mode, a 1-phenyl-1,2,3-triazol-4-yl *N*- or *O*-pendant drops the inhibitory action of acesulfame derivatives **53–59** because of steric hindrance reasons in CA IX and XII active sites. Consistently to the inhibition profile in Tables 1 and 2, the binding mode predicted for **2**, **46**, **49** and **51** in CA IX and XII is likely not allowed in the narrower and hindered active site of CA I and II (Supplementary Figure S2), in which the used computational protocol was not able to find reliable solutions^58^.

## 5. Conclusions

The design, synthesis and biological activity of novel saccharin- and acesulfame-based compounds have been described. The different approaches attempted for the development of the new inhibitors underlined the importance of the flexibility for the correct distribution of the molecular fragments inside the hCA IX/XII active site. Indeed, the removal of the methylene group in the *N*-benzyl saccharins to obtain the *N*-phenyl analogues elicited detrimental effects, with the only exception of the opened-saccharin derivative **24**. Similarly, for the triazole bearing derivatives, the insertion of a second methylene group separating the triazole moiety from the outer phenyl ring improved the activity. Docking and MD studies described the most probable binding mode of the four most active compounds (**2**, **46**, **49**, **51**) based on the anchoring to zinc-bound water, also confirming and explaining the correlation between inhibitory activity and the ability to occupy sites in order to establish interactions that influenced the binding affinity.

## Supplementary Material

Supplemental Material

## Supplementary data

Additional figures illustrating molecular modelling data, synthesis description, NMR spectroscopic data and the chromatographic profile for purity assessment of each compound are available as supplementary data.

## References

[1] Supuran CT. Exploring the multiple binding modes of inhibitors to carbonic anhydrases for novel drug discovery. Expert Opin Drug Discov 2020;15:671–86. 32208982 10.1080/17460441.2020.1743676

[2] Supuran CT. Structure and function of carbonic anhydrases. Biochem J 2016;473:2023–32. 27407171 10.1042/BCJ20160115

[3] Supuran CT. Carbonic anhydrases: novel therapeutic applications for inhibitors and activators. Nat Rev Drug Discov 2008;7:168–81. 18167490 10.1038/nrd2467

[4] Supuran CT. Carbonic anhydrases an overview. Curr Pharm Des 2008;14:603–14. 18336305 10.2174/138161208783877884

[5] Supuran CT, Scozzafava A. Carbonic anhydrase inhibitors and their therapeutic potential. Expert Opin Ther Pat 2000;10:575–600.

[6] Supuran CT, Scozzafava A. Carbonic anhydrases as targets for medicinal chemistry. Bioorganic Med Chem 2007;15:4336–50. 10.1016/j.bmc.2007.04.020 17475500

[7] Nocentini A, Supuran CT. Chapter 1 – carbonic anhydrases: an overview. In: Supuran C.T., Nocentini A.B.T.-C.A. eds. Carbonic anhydrases. Washington, DC: Academic Press; 2019:3–16.

[8] Nocentini A, Donald WA, Supuran CT, Chapter 8 – human carbonic anhydrases: tissue distribution, physiological role, and druggability, In: Supuran C.T., Nocentini A.B.T.-C.A. eds., Carbonic anhydrases. Washington, DC: Academic Press; 2019: 151–185.

[9] Provensi G, Carta F, Nocentini A, et al. A new kid on the block? Carbonic anhydrases as possible new targets in Alzheimer’s disease. Int J Mol Sci 2019;20:4724. 10.3390/ijms20194724 PMC6801497 31554165

[10] Gul HI, Yamali C, Sakagami H, et al. New anticancer drug candidates sulfonamides as selective hCA IX or hCA XII inhibitors. Bioorg Chem 2018;77:411–9. 29427856 10.1016/j.bioorg.2018.01.021

[11] Supuran CT. Carbonic anhydrase activators. Future Med Chem 2018;10:561–73. 29478330 10.4155/fmc-2017-0223

[12] Solesio ME, Peixoto PM, Debure L, et al. Carbonic anhydrase inhibition selectively prevents amyloid β neurovascular mitochondrial toxicity. Aging Cell 2018;17:e12787. 29873184 10.1111/acel.12787 PMC6052473

[13] Swietach P, Wigfield S, Cobden P, et al. Tumor-associated carbonic anhydrase 9 spatially coordinates intracellular pH in three-dimensional multicellular growths. J Biol Chem 2008;283:20473–83. 18482982 10.1074/jbc.M801330200

[14] De Simone G, Supuran CT. Carbonic anhydrase IX: biochemical and crystallographic characterization of a novel antitumor target. Biochim Biophys Acta 2010;1804:404–9. 19679200 10.1016/j.bbapap.2009.07.027

[15] Ameis HM, Drenckhan A, Freytag M, et al. Carbonic anhydrase IX correlates with survival and is a potential therapeutic target for neuroblastoma. J Enzyme Inhib Med Chem 2015;0:1–6. 10.3109/14756366.2015.1029471 25884234

[16] Pastorekova S, Ratcliffe PJ, Pastorek J. Molecular mechanisms of carbonic anhydrase IX-mediated pH regulation under hypoxia. BJU Int 2008;101:8–15. 18430116 10.1111/j.1464-410X.2008.07642.x

[17] Thiry A, Dogné JM, Masereel B, Supuran CT. Targeting tumor-associated carbonic anhydrase IX in cancer therapy. Trends Pharmacol Sci 2006;27:566–73. 16996620 10.1016/j.tips.2006.09.002

[18] Lounnas N, Rosilio C, Nebout M, et al. Pharmacological inhibition of carbonic anhydrase XII interferes with cell proliferation and induces cell apoptosis in T-cell lymphomas. Cancer Lett 2013;333:76–88. 23348702 10.1016/j.canlet.2013.01.020

[19] Supuran CT. Advances in structure-based drug discovery of carbonic anhydrase inhibitors. Expert Opin Drug Discov 2017;12:61–88. 27783541 10.1080/17460441.2017.1253677

[20] Nocentini A, Supuran CT. Advances in the structural annotation of human carbonic anhydrases and impact on future drug discovery. Expert Opin Drug Discov 2019;14:1175–97. 31436118 10.1080/17460441.2019.1651289

[21] Berrino E, Supuran CT. Novel approaches for designing drugs that interfere with pH regulation. Expert Opin Drug Discov 2019;14:231–48. 30681011 10.1080/17460441.2019.1567488

[22] Supuran CT. Structure-based drug discovery of carbonic anhydrase inhibitors. J Enzyme Inhib Med Chem 2012;27:759–72. 22468747 10.3109/14756366.2012.672983

[23] Güzel-Akdemir Ö, Akdemir A, Karalı N, Supuran CT. Discovery of novel isatin-based sulfonamides with potent and selective inhibition of the tumor-associated carbonic anhydrase isoforms IX and XII. Org Biomol Chem 2015;13:6493–9. 25967275 10.1039/c5ob00688k

[24] Di Fiore A, Monti SM, Hilvo M, et al. Crystal structure of human carbonic anhydrase XIII and its complex with the inhibitor acetazolamide. Proteins Struct Funct Bioinforma 2009;74:164–75. 10.1002/prot.22144 18618712

[25] Angeli A, Pinteala M, Maier SS, et al. Evaluation of thio-and seleno-acetamides bearing benzenesulfonamide as inhibitor of carbonic anhydrases from different pathogenic bacteria. Int J Mol Sci 2020;21:1–8. 10.3390/ijms21020598 PMC7014678 31963423

[26] Angeli A, Peat TS, Selleri S, et al. X-ray crystallography of Epacadostat in adduct with Carbonic Anhydrase IX. Bioorg Chem 2020;97:103669. 32088421 10.1016/j.bioorg.2020.103669

[27] Carradori S, Guglielmi P, Chapter 12, Mechanisms of action of carbonic anhydrase inhibitors: compounds that bind “out of the binding site” and compounds with an unknown mechanism of action. In: Supuran C.T., Nocentini A.B.T.-C.A. eds., Carbonic Anhydrases. Washington, DC: Academic Press; 2019: 257–268.

[28] Supuran CT. How many carbonic anhydrase inhibition mechanisms exist? J Enzyme Inhib Med Chem 2016;31:345–60. 26619898 10.3109/14756366.2015.1122001

[29] Scozzafava A, Carta F, Supuran CT. Secondary and tertiary sulfonamides: a patent review (2008–2012). Expert Opin Ther Pat 2013;23:203–13. 23148584 10.1517/13543776.2013.742065

[30] Alterio V, Di Fiore A, D’Ambrosio K, et al. Multiple binding modes of inhibitors to carbonic anhydrases: how to design specific drugs targeting 15 different isoforms? Chem Rev 2012;112:4421–68. 22607219 10.1021/cr200176r

[31] Köhler K, Hillebrecht A, Schulze Wischeler J, et al. Saccharin inhibits carbonic anhydrases: possible explanation for its unpleasant metallic aftertaste. Angew Chem Int Ed Engl 2007;46:7697–9. 17705204 10.1002/anie.200701189

[32] Coviello V, Marchi B, Sartini S, et al. 1,2-benzisothiazole derivatives bearing 4-, 5-, or 6-alkyl/arylcarboxamide moieties inhibit carbonic anhydrase isoform IX (CAIX) and cell proliferation under hypoxic conditions. J Med Chem 2016;59:6547–52. 27305384 10.1021/acs.jmedchem.6b00616

[33] Zubriene A, Čapkauskaite E, Gylyte J, et al. Benzenesulfonamides with benzimidazole moieties as inhibitors of carbonic anhydrases I, II, VII, XII and XIII. J Enzyme Inhib Med Chem 2014;29:124–31. 23356363 10.3109/14756366.2012.757223

[34] Ivanova J, Leitans J, Tanc M, et al. X-ray crystallography-promoted drug design of carbonic anhydrase inhibitors. Chem Commun 2015;51:7108–11. 10.1039/c5cc01854d 25813715

[35] Alterio V, Tanc M, Ivanova J, et al. X-ray crystallographic and kinetic investigations of 6-sulfamoyl-saccharin as a carbonic anhydrase inhibitor. Org Biomol Chem 2015;13:4064–9. 25733161 10.1039/c4ob02648a

[36] Ivanova J, Carta F, Vullo D, et al. N-Substituted and ring opened saccharin derivatives selectively inhibit transmembrane, tumor-associated carbonic anhydrases IX and XII. Bioorganic Med Chem 2017;25:3583–9. 10.1016/j.bmc.2017.04.007 28416101

[37] Carradori S, Secci D, De Monte C, et al. A novel library of saccharin and acesulfame derivatives as potent and selective inhibitors of carbonic anhydrase IX and XII isoforms. Bioorg Med Chem 2016;24:1095–105. 26810710 10.1016/j.bmc.2016.01.038

[38] D’Ascenzio M, Guglielmi P, Carradori S, et al. Open saccharin-based secondary sulfonamides as potent and selective inhibitors of cancer-related carbonic anhydrase IX and XII isoforms. J Enzyme Inhib Med Chem 2017;32:51–9. 27784170 10.1080/14756366.2016.1235040 PMC6009879

[39] D’Ascenzio M, Carradori S, De Monte C, et al. Design, synthesis and evaluation of N-substituted saccharin derivatives as selective inhibitors of tumor-associated carbonic anhydrase XII. Bioorg Med Chem 2014;22:1821–31. 24560739 10.1016/j.bmc.2014.01.056

[40] Uda NR, Seibert V, Stenner-Liewen F, et al. Esterase activity of carbonic anhydrases serves as surrogate for selecting antibodies blocking hydratase activity. J Enzyme Inhib Med Chem 2015;30:955–60. 25775095 10.3109/14756366.2014.1001754

[41] Rotondi G, Guglielmi P, Carradori S, et al. Design, synthesis and biological activity of selective hCAs inhibitors based on 2-(benzylsulfinyl)benzoic acid scaffold. J Enzyme Inhib Med Chem 2019;34:1400–13. 31401897 10.1080/14756366.2019.1651315 PMC6713143

[42] D’Ascenzio M, Secci D, Carradori S, et al. 1,3-dipolar cycloaddition, HPLC enantioseparation, and docking studies of saccharin/isoxazole and saccharin/isoxazoline derivatives as selective carbonic anhydrase IX and XII inhibitors. J Med Chem 2020;63:2470–88. 31972093 10.1021/acs.jmedchem.9b01434

[43] El-Gazzar MG, Nafie NH, Nocentini A, et al. Carbonic anhydrase inhibition with a series of novel benzenesulfonamide-triazole conjugates. J Enzyme Inhib Med Chem 2018;33:1565–74. 30274535 10.1080/14756366.2018.1513927 PMC6171417

[44] Nocentini A, Ferraroni M, Carta F, et al. Benzenesulfonamides incorporating flexible triazole moieties are highly effective carbonic anhydrase inhibitors: synthesis and kinetic, crystallographic, computational, and intraocular pressure lowering investigations. J Med Chem 2016;59:10692–704. 27933963 10.1021/acs.jmedchem.6b01389

[45] Kumar R, Vats L, Bua S, et al. Design and synthesis of novel benzenesulfonamide containing 1,2,3-triazoles as potent human carbonic anhydrase isoforms I, II, IV and IX inhibitors. Eur J Med Chem 2018;155:545–51. 29909339 10.1016/j.ejmech.2018.06.021

[46] Vats L, Sharma V, Angeli A, et al. Synthesis of novel 4-functionalized 1,5-diaryl-1,2,3-triazoles containing benzenesulfonamide moiety as carbonic anhydrase I, II, IV and IX inhibitors. Eur J Med Chem 2018;150:678–86. 29571155 10.1016/j.ejmech.2018.03.030

[47] Murray AB, Lomelino CL, Supuran CT, McKenna R. “Seriously Sweet”: acesulfame K exhibits selective inhibition using alternative binding modes in carbonic anhydrase isoforms. J Med Chem 2018;61:1176–81. 29266943 10.1021/acs.jmedchem.7b01470

[48] De Monte C, Carradori S, Secci D, et al. Cyclic tertiary sulfamates: selective inhibition of the tumor-associated carbonic anhydrases IX and XII by N- and O-substituted acesulfame derivatives. Eur J Med Chem 2014;84:240–6. 25019479 10.1016/j.ejmech.2014.07.014

[49] Khalifah RG. The carbon dioxide hydration activity of carbonic anhydrase. I. Stop-flow kinetic studies on the native human isoenzymes B and C. J Biol Chem 1971;246:2561–73. 4994926

[50] Bruno E, Buemi MR, Di Fiore A, et al. Probing molecular interactions between human carbonic anhydrases (hCAs) and a novel class of benzenesulfonamides. J Med Chem 2017;60:4316–26. 28453941 10.1021/acs.jmedchem.7b00264

[51] Angeli A, Vaiano F, Mari F, et al. Psychoactive substances belonging to the amphetamine class potently activate brain carbonic anhydrase isoforms VA, VB, VII, and XII. J Enzyme Inhib Med Chem 2017;32:1253–9. 28936885 10.1080/14756366.2017.1375485 PMC6009978

[52] Angeli A, Tanini D, Capperucci A, et al. Synthesis of different thio-scaffolds bearing sulfonamide with subnanomolar carbonic anhydrase II and IX inhibitory properties and X-ray investigations for their inhibitory mechanism. Bioorg Chem 2018;81:642–8. 30253337 10.1016/j.bioorg.2018.09.028

[53] Mahon BP, Bhatt A, Socorro L, et al. The structure of carbonic anhydrase IX is adapted for low-pH catalysis. Biochemistry 2016;55:4642–53. 27439028 10.1021/acs.biochem.6b00243 PMC5322481

[54] Whittington DA, Waheed A, Ulmasov B, et al. Crystal structure of the dimeric extracellular domain of human carbonic anhydrase XII, a bitopic membrane protein overexpressed in certain cancer tumor cells. Proc Natl Acad Sci USA 2001;98:9545–50. 11493685 10.1073/pnas.161301298 PMC55489

[55] Schrödinger Suite Release 2019-1, Schrödinger, LLC, New York, NY, 2019:(a) Prime, v.5.5; Maestro v.11.9;(b) Epik, v.4.7; (c) Impact, v.8.2; (d) Macromodel v.12.3. (e) Glide, v.8.2.

[56] Chen FMF, Benoiton NL. The preparation and reactions of mixed anhydrides of N -alkoxycarbonylamino acids. Can J Chem 1987;65:619–25.

[57] Shao C, Wang X, Zhang Q, et al. Acid-base jointly promoted copper(I)-catalyzed azide-alkyne cycloaddition. J Org Chem 2011;76:6832–6. 21793533 10.1021/jo200869a

[58] Ghirga F, Quaglio D, Ghirga P, et al. Occurrence of enantioselectivity in nature: The case of (S)-norcoclaurine. Chirality 2016;28:169–80. 10.1002/chir.22566 26729048

