# Supplementary material for: Novel insights on saccharin- and acesulfame-based carbonic anhydrase inhibitors: design, synthesis, modelling investigations and biological activity evaluation
Source: J Enzyme Inhib Med Chem. 2020 Oct 2;35(1):1891–905. doi: 10.1080/14756366.2020.1828401 (PMC7580763; doi:10.1080/14756366.2020.1828401)
Supplement: Supplemental Material [file IENZ_A_1828401_SM6687.pdf]

Supplementary material

Novel insights on saccharin- and acesulfame-based carbonic anhydrase inhibitors: design, synthesis, modelling investigations and biological activity evaluation

Paolo Guglielmi[a,§], Giulia Rotondi[a,§], Daniela Secci[a], Andrea Angeli[b,c], Paola Chimenti[a], Alessio Nocentini[b,d], Alessandro Bonardi[b,d], Paola Gratteri[b,d], Simone Carradori[e,*], Claudiu T. Supuran[b,*]

[a]*Dipartimento di Chimica e Tecnologie del Farmaco, Sapienza University of Rome, P.le A. Moro 5, 00185 Rome, Italy.*

[b]*Neurofarba Department, Section of Pharmaceutical and Nutraceutical Sciences, University of Florence, Via U. Schiff 6, 50019 Sesto Fiorentino (Florence), Italy.*

[c]*Centre of Advanced Research in Bionanoconjugates and Biopolymers Department, "Petru Poni" Institute of Macromolecular Chemistry, 700487, Iasi, Romania.*

[d]*Neurofarba Department, Section of Pharmaceutical and Nutraceutical Sciences, Laboratory of Molecular Modeling Cheminformatics & QSAR University of Florence, Via U. Schiff 6, 50019 Sesto Fiorentino (Florence), Italy.*

[e]*Department of Pharmacy, "G. d'Annunzio" University of Chieti-Pescara, Via dei Vestini 31, 66100 Chieti, Italy.*

[§]The two authors contributed equally to this work.

[*]Corresponding authors: simone.carradori@unich.it (S. Carradori), claudiu.supuran@unifi.it (C.T. Supuran).

Table of Contents

Supplemental molecular modelling figures	S2
Synthesis procedures and characterization data of the proposed derivatives	S3-S20
Examples of representative ^{1}H-NMR and ^{13}C-NMR spectra	S21-S65
HPLC chromatograms of compounds **1-60**	S66-S70
References	S72

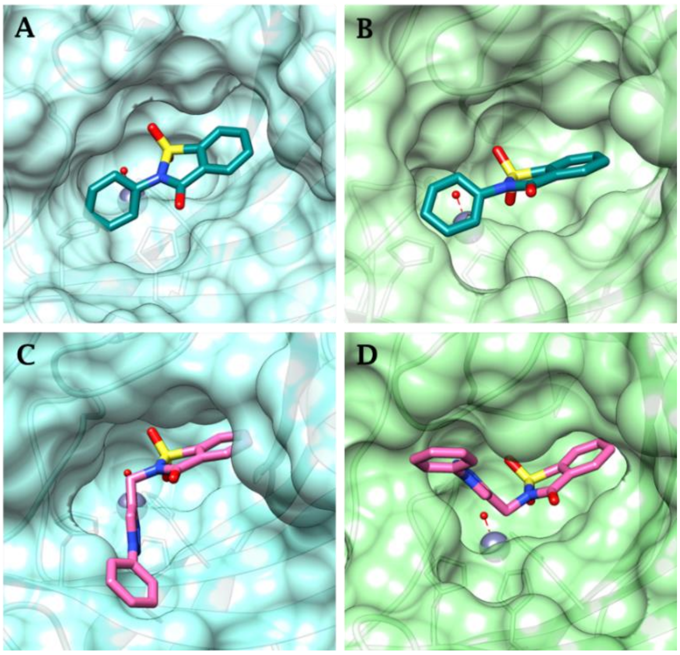

Figure S1. Replacement of the benzyl moiety of **2** and **46** with a phenyl ring (to give **29** and **41**, respectively) within the ligand/target complexes found by docking. Compound **29** in the active site of A) CA IX and B) CA XII; compound **41** in the active site of C) CA IX and D) CA XII. All graphical representations point out significant protein-ligand clashes.

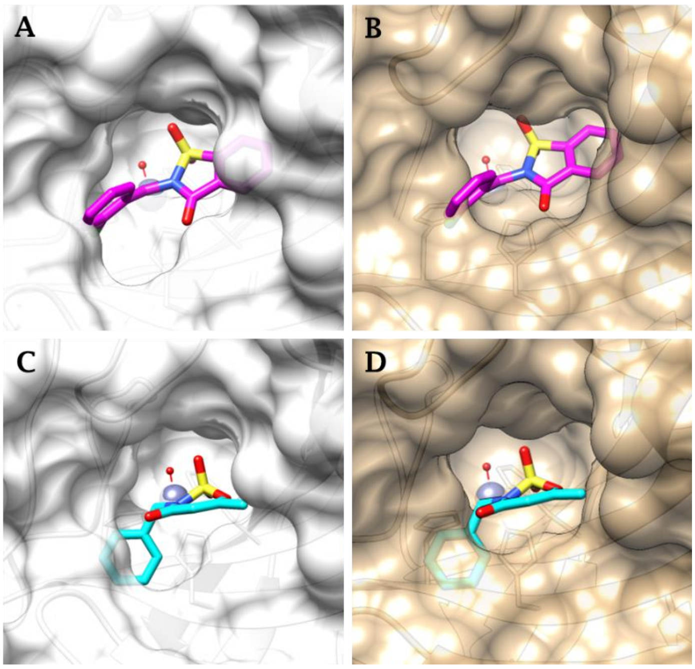

Figure S2. Representation of the CA IX predicted binding mode of **2** and **51** in CA I and CA II after isoforms overlay. (A) **2-**CA I, (B) **2-**CA II, (C) **51-**CA I and (D) **51-**CA II. All graphical representations point out significant protein-ligand clashes.

Synthesis procedures and characterization data of the proposed derivatives

2-(prop-2-yn-1-yl)benzo[*d*]isothiazol-3(2*H*)-one 1,1-dioxide (S1): for the synthesis and characterization data of the compound **S1**, see ref. [1].

6-methyl-3-(prop-2-yn-1-yl)-1,2,3-oxathiazin-4(3*H*)-one 2,2-dioxide (ACEN): propargyl bromide (1.1 equiv.) was added to a stirring solution of potassium acesulfame (1.0 equiv.) in *N*,*N*-dimethylformamide and stirred under nitrogen atmosphere for 24 h at 80 °C. Upon completion the mixture was poured on ice and extracted with dichloromethane (3 × 20 mL). The organic layers were reunited, dried over sodium sulfate and concentrated *in vacuo*. Purification through column chromatography on silica gel (petroleum ether:ethyl acetate, 5:1) afforded the title compound as a yellow oil (51% yield). ^{1}H NMR (400 MHz, $CDCl_3$): δ 2.18 (d, J = 1.0 Hz, 3H, CH_3), 2.31 (t, J = 2.5 Hz, 1H, CH=), 4.53 (d, J = 2.5 Hz, 2H, CH_2), 5.79 (d, J = 1.0 Hz, 1H, CH≡).^{13}C NMR (101, MHz, $CDCl_3$): δ 19.8 (CH_3), 31.6 (CH_2), 73.6 (CH≡), 75.9 (C≡), 104.3 (CH=C), 159.3 (CO), 162.2 ($COSO_2$).

6-methyl-4-(prop-2-yn-1-yloxy)-1,2,3-oxathiazine 2,2-dioxide (ACEO): propargyl bromide (1.1 equiv.) was added to a stirring solution of potassium acesulfame (1.0 equiv.) in *N*,*N*-dimethylformamide and stirred under nitrogen atmosphere for 24 h at room temperature. Once the completion the mixture was poured on ice and extracted with dichloromethane (3 × 20 mL). The organic layers were reunited, dried over sodium sulfate and concentrated *in vacuo*. Purification through column chromatography on silica gel (petroleum ether:ethyl acetate, 4:1) gave the title compound as a yellow oil (40% yield). For the characterization data see reference [2].

Azide derivatives

$NaNO_2$ (1.2 equiv.) was slowly added to a solution of the proper aniline (1.0 equiv.) in a 4M HCl aqueous solution at 0 °C. After 30 min NaN_3 (1.5 equiv.) was added portion-wise and the resulting mixture stirred at room temperature for 1 h. The mixture was then extracted with ethyl acetate (3 × 20 mL). The organic layers were reunited, dried over sodium sulfate and concentrated *in vacuo*. The residual crude product was used directly without further purification. For the characterization data of azides **I-VI**, see references [3–5].

2-(3-methylbut-2-en-1-yl)benzo[*d*]isothiazol-3(2*H*)-one 1,1-dioxide (1): anhydrous potassium carbonate (1 equiv.) was added to a stirring solution of saccharin (1 equiv.) in *N*,*N*-dimethylformamide. After 15 min, 3,3-dimethylallyl bromide (1 equiv.) was added and the suspension stirred under nitrogen atmosphere for 24 h at 0 °C. Once the reaction completion the mixture was poured on ice. The resulting suspension was filtered and the collected solid washed with chloroform giving the title compound as an oil (57% yield); ^{1}H NMR (300 MHz, $CDCl_3$): δ 1.71 (s, 3H, CH_3), 1.80 (s, 3H, CH_3), 4.33 (d, J = 7.2 Hz, 2H, CH_2), 5.32-5.37 (m, 1H, CH=), 7.74-7.89 (m, 3H, Ar), 7.97-8.00 (m, 1H, Ar). ^{13}C NMR (76 MHz, $CDCl_3$): δ 17.9 (CH_3), 25.8 (CH_3), 37.1 (CH_2), 116.9 (Ar), 120.9 (CH=), 125.1 (Ar), 127.5 (Ar), 134.2 (Ar), 134.6 (C=), 137.8 (Ar), 138.9 (Ar), 158.7 (C=O).

2-(2-chlorobenzyl)benzo[*d*]isothiazol-3(2*H*)-one 1,1-dioxide (2): anhydrous potassium carbonate (1 equiv.) was added to a stirring solution of saccharin (1 equiv.) in *N*,*N*-dimethylformamide. After 15 min, 1-(bromomethyl)-2-chlorobenzene (1 equiv.) was added and the suspension stirred under nitrogen atmosphere for 8 h at 80 °C. Once the reaction completion the mixture was poured on ice. The resulting suspension was filtered and the collected solid washed with *n*-hexane, to afford the title compound as a yellow solid (65% yield); mp 143-145°C; ^{1}H NMR (300 MHz, $CDCl_3$): δ 5.11 (s, 2H, CH_2), 7.28 (s, 2H, Ar), 7.44 (d, J = 2.4 Hz, 2H, Ar), 7.87-7.96 (m, 3H, Ar), 8.12 (d, J = 4.8 Hz, 1H, Ar). ^{13}C NMR (76 MHz, $CDCl_3$): δ 40.2 (CH_2), 121.1 (Ar), 125.4 (Ar), 127.2 (2 × Ar), 129.2 (Ar), 129.4 (Ar), 129.7 (Ar), 131.9 (Ar), 133.3 (Ar), 134.4 (Ar), 135.0 (Ar), 137.9 (Ar), 158.9 (C=O).

2-(3-chlorobenzyl)benzo[*d*]isothiazol-3(2*H*)-one 1,1-dioxide (3): anhydrous potassium carbonate (1 equiv.) was added to a stirring solution of saccharin (1 equiv.) in *N*,*N*-dimethylformamide. After 15 min, 1-(bromomethyl)-3-chlorobenzene (1 equiv.) was added and the suspension stirred under nitrogen atmosphere for 12 h at 80 °C. Once the reaction completion the mixture was poured on ice. The resulting suspension was filtered and the collected solid washed with *n*-hexane, to afford the title compound as a yellow solid (57% yield); mp 78-80 °C; ^{1}H NMR (300 MHz, $CDCl_3$): δ 4.85 (s, 2H, CH_2), 7.24-7.26 (m, 2H, Ar), 7.35-7.38 (m, 1H, Ar), 7.47 (s, 1H, Ar), 7.75-7.84 (m, 2H, Ar), 7.88-7.91 (m, 1H, Ar), 7.99-8.02 (m, 1H, Ar). ^{13}C NMR (76 MHz, $CDCl_3$): δ 41.9 (CH_2), 121.1 (Ar), 125.3 (Ar), 126.8 (Ar), 127.0 (Ar), 128.5 (Ar), 128.7 (Ar), 130.0 (Ar), 134.5 (Ar), 134.5 (Ar), 135.0 (Ar), 136.6 (Ar), 137.5 (Ar), 158.9 (C=O).

2-(2-(3-nitrophenyl)-2-oxoethyl)benzo[*d*]isothiazol-3(2*H*)-one 1,1-dioxide (4): anhydrous potassium carbonate (1 equiv.) was added to a stirring solution of saccharin (1 equiv.) in *N*,*N*-dimethylformamide. After 15 min, 2-bromo-3'-nitroacetophenone (1 equiv.) was added and the suspension stirred under nitrogen atmosphere for 24 h at 80 °C. Once the reaction completion the mixture was poured on ice. The resulting suspension was filtered and the collected solid washed with *n*-hexane to afford the title compound as a yellow solid (65% yield); mp 190-191 °C; ^{1}H NMR (300 MHz, $CDCl_3$): δ 5.19 (s, 2H, CH_2), 7.77 (t, J = 7.8 Hz, 1H, Ar), 7.89-8.01 (m, 3H, Ar), 8.12-8.14 (m, 1H, Ar), 8.33-8.36 (m, 1H, Ar), 8.50-8.53 (m, 1H, Ar), 8.85 (t, J = 1.5 Hz, 1H, Ar). ^{13}C NMR (76 MHz, $CDCl_3$): δ 44.5 (CH_2), 121.3 (Ar), 123.1 (Ar), 123.7 (Ar), 125.6 (Ar), 127.5 (Ar), 128.5 (Ar), 130.4 (Ar), 133.7 (Ar), 134.6 (Ar), 135.2 (Ar), 135.2 (Ar), 137.6 (Ar), 148.5 (C=O), 187.3 (C=O).

Synthesis and characterization of derivatives 5-16

To a stirring solution of the proper aniline (1.0 equiv.) in dichloromethane at 0 °C was added pyridine (1.2 equiv.). After 20 min, methyl 2-(chlorosulfonyl)benzoate (1 equiv.) was added portionwise and the reaction stirred under nitrogen atmosphere for 2 h. Once the synthesis completion the mixture was extracted with dichloromethane (3 × 20 mL) and 2N HCl (1 × 10 mL). The organic layers were

reunited, dried over sodium sulfate and concentrated *in vacuo*. Purification through column chromatography on silica gel, using the proper solvents, gave compounds **5-16** as white or yellow solids.

Methyl 2-(*N*-phenylsulfamoyl)benzoate (5): white solid, mp 70-72 °C; ^{1}H NMR (400 MHz, $CDCl_3$): δ 4.07 (s, 3H, OCH_3), 7.11-7.15 (m, 1H, Ar), 7.17-7.19 (m, 2H, Ar), 7.22-7.26 (m, 2H, Ar), 7.45-7.51 (m, 1H, Ar), 7.57-7.61 (m, 1H, Ar), 7.82-7.83 (m, 1H, Ar), 7.84-7.85 (m, 1H, Ar), 8.04 (br s, 1H, SO_2NH, D_2O exch.). ^{13}C NMR (101 MHz, $CDCl_3$): δ 53.6 (OCH_3), 122.9 (2 × Ar), 125.8 (Ar), 129.2 (2 × Ar), 130.4 (Ar), 130.5 (Ar), 130.6 (Ar), 131.5 (Ar), 132.6 (Ar), 136.6 (Ar), 138.0 (Ar), 168.3 (C=O).

Methyl 2-(*N*-(4-fluorophenyl)sulfamoyl)benzoate (6): white solid, mp 102-104 °C; ^{1}H NMR (400 MHz, $CDCl_3$): δ 4.07 (s, 3H, OCH_3), 6.90-6.94 (m, 2H, Ar), 7.12-7.15 (m, 2H, Ar), 7.48-7.52 (m, 1H, Ar), 7.59-7.63 (m, 1H, Ar), 7.77-7.79 (m, 1H, Ar), 7.84-7.86 (m, 1H, Ar), 8.02 (br s, 1H, SO_2NH, D_2O exch.). ^{13}C NMR (101 MHz, $CDCl_3$): δ 53.6 (OCH_3), 116.0 (d, $^{2}J_{C\text{-}F}$ = 22.2 Hz, 2 × Ar), 125.4 (d, $^{3}J_{C\text{-}F}$ = 8.4 Hz, 2 × Ar), 130.5 (Ar), 130.6 (Ar), 130.6 (Ar), 131.5 (Ar), 132.5 (d, $^{4}J_{C\text{-}F}$ = 3.03 Hz, Ar), 132.7 (Ar), 137.7 (Ar), 160.9 (d, $^{1}J_{C\text{-}F}$ = 245.5 Hz, C-F), 168.4 (C=O).

Methyl 2-(*N*-(2-chlorophenyl)sulfamoyl)benzoate (7): white solid, mp 90-92 °C; ^{1}H NMR (400 MHz, $CDCl_3$): δ 4.07 (s, 3H, OCH_3), 7.06-7.10 (m, 1H, Ar), 7.25-7.29 (m, 2H, Ar), 7.53-7.58 (m, 1H, Ar), 7.62-7.66 (m, 1H, Ar), 7.74-7.76 (m, 1H, Ar), 7.87-7.91 (m, 2H, Ar), 8.51 (br s, 1H, SO_2NH, D_2O exch.). ^{13}C NMR (101 MHz, $CDCl_3$): δ 53.5 (OCH_3), 124.3 (Ar), 126.1 (Ar), 126.2 (Ar), 127.7 (Ar), 129.5 (2 x Ar), 130.6 (Ar), 131.0 (Ar), 131.6 (Ar), 132.9 (Ar), 133.8 (Ar), 138.9 (Ar), 167.5 (C=O).

Methyl 2-(*N*-(4-chlorophenyl)sulfamoyl)benzoate (8): white solid, mp 108-112 °C; ^{1}H NMR (400 MHz, $CDCl_3$): δ 4.07 (s, 3H, OCH_3), 7.11-7.14 (m, 2H, Ar), 7.19-7.22 (m, 2H, Ar), 7.50-7.54 (m, 1H, Ar), 7.59-7.63 (m, 1H, Ar), 7.82-7.86 (m, 2H, Ar), 8.09 (br s, 1H, SO_2NH, D_2O exch.). ^{13}C NMR (101 MHz, $CDCl_3$): δ 53.6 (OCH_3), 124.2 (2 × Ar), 129.3 (2 × Ar), 130.5 (Ar), 130.5 (Ar), 130.7 (Ar), 131.5 (Ar), 131.6 (Ar), 132.8 (Ar), 135.2 (Ar), 137.8 (Ar), 168.4 (C=O).

Methyl 2-(*N*-(4-bromophenyl)sulfamoyl)benzoate (9): white solid, mp 116-118 °C; ^{1}H NMR (400 MHz, $CDCl_3$): δ 4.08 (s, 3H, OCH_3), 7.36-7.39 (m, 2H, Ar), 7.57-7.67 (m, 2H, Ar), 7.87-7.90 (m, 1H, Ar), 7.97-7.99 (m, 1H, Ar), 8.12-8.15 (m, 2H, Ar), 8.59 (br s, 1H, SO_2NH, D_2O exch.). ^{13}C NMR (101 MHz, $CDCl_3$): δ 53.6 (OCH_3), 119.2 (Ar), 124.4 (2 × Ar), 130.4 (Ar), 130.5 (Ar), 130.7 (Ar), 131.7 (Ar), 132.3 (2 × Ar), 132.8 (Ar), 135.8 (Ar), 137.7 (Ar), 168.4 (C=O).

Methyl 2-(*N*-(4-(trifluoromethyl)phenyl)sulfamoyl)benzoate (10): white solid, mp 92-96 °C; ^{1}H NMR (400 MHz, $CDCl_3$): δ 4.08 (s, 3H, OCH_3), 7.32 (d, J = 8.4 Hz, 2H, Ar), 7.50 (d, J = 8.5 Hz, 2H, Ar), 7.53-7.57 (m, 1H, Ar), 7.60-7.65 (m, 1H, Ar), 7.85-7.88 (m, 1H, Ar), 7.90-7.93 (m, 1H, Ar), 8.35 (br s, 1H, SO_2NH, D_2O exch.). ^{13}C NMR (101 MHz, DMSO-d_6): δ 53.7 (OCH_3), 121.6 (2 x Ar), 123.9 (d, $^1J_{C\text{-}F}$ = 271.9 Hz, CF_3), 126.5 (q, $^3J_{C\text{-}F}$ = 3.8 Hz, 2 x Ar), 127.4 (d, $^2J_{C\text{-}F}$ = 32.8 Hz, Ar), 130.4 (Ar), 130.4 (Ar), 130.9 (Ar),131.8 (Ar), 133.0 (Ar), 137.8 (Ar), 140.0 (Ar), 168.3 (C=O).

Methyl 2-(*N*-(3-nitrophenyl)sulfamoyl)benzoate (11): yellow solid, mp 168-170 °C; ^{1}H NMR (400 MHz, $CDCl_3$): δ 4.09 (s, 3H, OCH_3), 7.45 (t, J = 8.1 Hz, 1H, Ar), 7.53-7.67 (m, 3H, Ar), 7.63-7.67 (m, 1H, Ar), 7.88-7.91 (m, 1H, Ar), 7.96-7.99 (m, 1H, Ar), 8.01-8.02 (m, 1H, Ar), 8.50 (br s, 1H, SO_2NH, D_2O exch.). ^{13}C NMR (101 MHz, $CDCl_3$): δ 53.8 (OCH_3), 116.8 (Ar), 120.3 (Ar), 128.1 (Ar), 130.1 (Ar), 130.3 (Ar), 130.4 (Ar), 131.2 (Ar), 131.9 (Ar), 133.2 (Ar), 137.6 (Ar), 138.2 (Ar), 148.7 (Ar), 168.3 (C=O).

Methyl 2-(*N*-(4-nitrophenyl)sulfamoyl)benzoate (12): yellow solid, mp 170-174 °C; ^{1}H NMR (400 MHz, $CDCl_3$): δ 4.08 (s, 3H, CH_3), 7.35-7.39 (m, 2H, Ar), 7.56-7.61 (m, 1H, Ar), 7.63-7.67 (m, 1H, Ar), 7.87-7.89 (m, 1H, Ar), 7.97-7.99 (m, 1H, Ar), 8.11-8.15 (m, 2H, Ar), 8.60 (br s, 1H, SO_2NH, D_2O exch.). ^{13}C NMR (101 MHz, $CDCl_3$): δ 53.8 (OCH_3), 120.7 (2 × Ar), 125.1 (2 × Ar), 130.3 (Ar), 130.3 (Ar), 131.2 (Ar), 131.9 (Ar), 133.3 (Ar), 137.7 (Ar), 142.9 (Ar), 144.7 (Ar), 168.3 (C=O).

Methyl 2-(*N*-(*p*-tolyl)sulfamoyl)benzoate (13): white solid, mp 78-82 °C; ^{1}H NMR (400 MHz, $CDCl_3$): δ 2.27 (s, 3H, CH_3), 4.07 (s, 3H, OCH_3), 7.01-7.07 (m, 4H, Ar), 7.47-7.51 (m, 1H, Ar), 7.57-7.61 (m, 1H, Ar), 7.80-7.85 (m, 2H, Ar), 7.94 (br s, 1H, SO_2NH, D_2O exch.). ^{13}C NMR (101 MHz,

$CDCl_3$): δ 20.9 (CH_3), 53.5 (OCH_3), 123.3 (2 x Ar), 129.7 (2 x Ar), 130.5 (Ar), 130.5 (Ar), 130.5 (Ar), 131.4 (Ar), 132.5 (Ar), 133.9 (Ar), 135.8 (Ar), 138.1 (Ar), 168.4 (C=O).

Methyl 2-(*N*-(2,6-dimethylphenyl)sulfamoyl)benzoate (14): white solid, mp 170-174 °C; ^{1}H NMR (400 MHz, $CDCl_3$): δ 2.14 (s, 6H, 2 x CH_3), 4.08 (s, 3H, OCH_3), 7.03-7.12 (m, 3H, Ar), 7.57-7.61 (m, 1H, Ar), 7.64-7.68 (m, 1H, Ar), 7.77 (br s, 1H, SO_2NH, D_2O exch.), 7.87-7.91 (m, 2H, Ar). ^{13}C NMR (101 MHz, $CDCl_3$): δ 18.8 (2 x CH_3), 53.5 (OCH_3), 127.6 (Ar), 128.6 (2 x Ar), 128.8 (Ar), 130.2 (Ar), 130.5 (Ar), 131.9 (Ar), 132.2 (Ar), 133.4 (Ar), 137.7 (2 x Ar), 141.6 (Ar), 168.6 (C=O).

Methyl 2-(*N*-(4-isopropylphenyl)sulfamoyl)benzoate (15): white solid, mp 58-62 °C; ^{1}H NMR (400 MHz, $CDCl_3$): δ 1.19 (d, J = 6.9 Hz, 6H, 2 × CH_3), 2.80-2.87 (m, 1H, CH), 4.07 (s, 3H, OCH_3), 7.06-7.10 (m, 4H, Ar), 7.48-7.52 (m, 1H, Ar), 7.57-7.61 (m, 1H, Ar), 7.82-7.85 (m, 2H, Ar), 7.96 (br s, 1H, SO_2NH, D_2O exch.). ^{13}C NMR (101 MHz, $CDCl_3$): δ 23.9 (2 × CH_3), 35.5 (CH), 53.5 (OCH_3), 123.2 (2 × Ar), 127.1 (2 × Ar), 130.4 (Ar), 130.5 (Ar), 130.6 (Ar), 131.4 (Ar), 132.5 (Ar), 134.1 (Ar), 138.3 (Ar), 146.7 (Ar), 168.4 (C=O).

Methyl 2-(*N*-(4-methoxyphenyl)sulfamoyl)benzoate (16): white solid, mp 108-112 °C; ^{1}H NMR (400 MHz, $CDCl_3$): δ 3.76 (s, 3H, OCH_3), 4.07 (s, 3H, OCH_3), 6.73-6.77 (m, 2H, Ar), 7.04-7.08 (m, 2H, Ar), 7.48-7.50 (m, 1H, Ar), 7.57-7.61 (m, 1H, Ar), 7.75-7.77 (m, 1H, Ar), 7.83-7.86 (m, 2H, 1H Ar + SO_2NH, D_2O exch.). ^{13}C NMR (101 MHz, $CDCl_3$): δ 53.6 (OCH_3), 55.4 (OCH_3), 114.3 (2 x Ar), 125.7 (2 x Ar), 129.2 (Ar), 130.5 (Ar), 130.5 (Ar), 130.6 (Ar), 131.4 (Ar), 132.5 (Ar), 138.1 (Ar), 158.1 (Ar), 168.4 (C=O).

Synthesis and characterization of derivatives 17-28

To a stirring solution of the proper ester derivative (1 equiv.) in dioxane (8 mL) were added dropwise 2 mL of aqueous KOH (2.2 equiv.). The reaction was stirred at 50 °C for 48-72 h until completion of the synthesis; then the organic solvent was evaporated *in vacuo.* The basic solution, containing the potassium salt of the acidic derivative, was extracted at first with dichloromethane (3 × 20 mL) and

then acidified with 4N HCl. The resulting suspension was filtered and the collected solid washed with *n*-hexane to afford the title compounds as white or yellow solids.

2-(*N*-phenylsulfamoyl)benzoic acid (17): white solid, mp 160-162 °C; ^{1}H NMR (400 MHz, DMSO-d_6): δ 7.03 (t, J = 7.3 Hz, 1H, Ar), 7.10 (d, J = 7.6 Hz, 2H, Ar), 7.23 (t, J = 7.9 Hz, 2H, Ar), 7.58-7.64 (m, 1H, Ar), 7.67 (d, J = 3.9 Hz, 2H, Ar), 7.79 (d, J = 7.7 Hz, 1H, Ar), 9.94 (br s, 1H, SO_2NH, D_2O exch.), 13.65 (br s, 1H, COOH, D_2O exch.). ^{13}C NMR (101 MHz, DMSO-d_6): δ 120.7 (2 × Ar), 124.7 (Ar), 128.9 (Ar), 129.6 (2 × Ar), 129.6 (Ar), 131.0 (Ar), 133.4 (Ar), 134.2 (Ar), 137.0 (Ar), 137.8 (Ar), 169.0 (COOH).

2-(*N*-(4-fluorophenyl)sulfamoyl)benzoic acid (18): white solid, mp 127-129 °C; ^{1}H NMR (400 MHz, DMSO-d_6): δ 7.06-7.13 (m, 4H, Ar), 7.55-7.62 (m, 1H, Ar), 7.64-7.69 (m, 2H, Ar), 7.73-7.75 (m, 1H, Ar), 10.04 (br s, 1H, SO_2NH, D_2O exch.), 13.72 (br s, 1H, COOH, D_2O exch.). ^{13}C NMR (101 MHz, DMSO-d_6): δ 116.3 (d, $^2J_{C\text{-}F}$ = 22.7 Hz, 2 × Ar), 123.6 (d, $^3J_{C\text{-}F}$ = 8.28 Hz, 2 × Ar), 128.9 (Ar), 129.7 (Ar), 130.8 (Ar), 133.4 (Ar), 134.1 (d, $^4J_{C\text{-}F}$ = 2.2 Hz, Ar), 134.6 (Ar), 136.7 (Ar), 159.6 (d, $^1J_{C\text{-}F}$ = 241.1, C-F), 169.0 (COOH).

2-(*N*-(2-chlorophenyl)sulfamoyl)benzoic acid (19): white solid, mp 158-160 °C; ^{1}H NMR (400 MHz, DMSO-d_6): δ 7.13-7.17 (m, 1H, Ar), 7.25-7.29 (m, 1H, Ar), 7.36-7.42 (m, 2H, Ar), 7.59-7.63 (m, 1H, Ar), 7.69-7.73 (m, 1H, Ar), 7.78-7.82 (m, 2H, Ar), 10.16 (br s, 1H, SO_2NH, D_2O exch.). ^{13}C NMR (101 MHz, DMSO-d_6): δ 125.4 (Ar), 127.1 (Ar), 127.5 (Ar), 128.3 (Ar), 128.6 (Ar), 130.3 (Ar), 130.8 (Ar), 130.9 (Ar), 130.9 (Ar), 133.6 (Ar), 134.6 (Ar), 138.0 (Ar), 169.1 (COOH).

2-(*N*-(4-chlorophenyl)sulfamoyl)benzoic acid (20): white solid, mp 156-158 °C; ^{1}H NMR (400 MHz, DMSO-d_6): δ 7.10-7.13 (m, 2H, Ar), 7.28-7.32 (m, 2H, Ar), 7.60-7.64 (m, 1H, Ar), 7.65-7.69 (m, 2H, Ar), 7.80 (d, J = 7.9 Hz, 1H, Ar), 10.13 (br s, 1H, SO_2NH, D_2O exch.), 13.64 (br s, 1H, COOH, D_2O exch.). ^{13}C NMR (101 MHz, DMSO-d_6): δ 122.3 (2 × Ar), 128.8 (Ar), 128.9 (Ar), 129.5 (2 × Ar), 129.6 (Ar), 131.0 (Ar), 133.6 (Ar), 134.3 (Ar), 136.7 (Ar), 136.8 (Ar), 168.9 (COOH).

2-(*N*-(4-bromophenyl)sulfamoyl)benzoic acid (21): white solid, mp 134-136 °C; ^{1}H NMR (400 MHz, DMSO-d_6): δ 7.03-7.06 (m, 2H, Ar), 7.39-7.42 (m, 2H, Ar), 7.45-7.48 (m, 1H, Ar), 7.57-7.61

(m, 1H, Ar), 7.65-7.69 (m, 2H, Ar), 11.61 (br s, 1H, SO_2NH, D_2O exch.). ^{13}C NMR (101 MHz, DMSO-d_6): δ 116.5 (Ar), 123.0 (2 × Ar), 127.7 (Ar), 129.2 (Ar), 130.0 (Ar), 131.9 (2 × Ar), 132.8 (Ar), 136.0 (Ar), 137.3 (Ar), 137.6 (Ar), 168.7 (COOH).

2-(*N*-(4-(trifluoromethyl)phenyl)sulfamoyl)benzoic acid (22): white solid, mp 232-236 °C; ^1H NMR (400 MHz, DMSO-d_6): δ 7.29 (d, J = 8.4 Hz, 2H, Ar), 7.34-7.38 (m, 1H, Ar), 7.51-7.57 (m, 3H, Ar), 7.61-7.63 (m, 1H, Ar), 7.72-7.74 (m, 1H, Ar). ^{13}C NMR (101 MHz, DMSO-d_6): δ 121.5 (2 x Ar), 124.4 (Ar), 124.7 (d, $^1J_{C-F}$ = 271.4 Hz, CF_3), 126.7 (2 x Ar), 127.4 (Ar), 128.7 (Ar), 131.4 (Ar), 133.0 (Ar), 136.5 (Ar), 140.3 (Ar), 143.2 (Ar), 169.7 (COOH).

2-(*N*-(3-nitrophenyl)sulfamoyl)benzoic acid (23): yellow solid, mp 170-172 °C; ^1H NMR (400 MHz, DMSO-d_6): δ 7.51-7.57 (m, 2H, Ar), 7.63-7.72 (m, 3H, Ar), 7.86-7.90 (m, 2H, Ar), 7.97-7.98 (m, 1H, Ar), 10.69 (br s, 1H, SO_2NH, D_2O exch.), 13.63 (br s, 1H, COOH, D_2O exch.). ^{13}C NMR (101 MHz, DMSO-d_6): δ 114.4 (Ar), 119.0 (Ar), 126.0 (Ar), 129.0 (Ar), 129.6 (Ar), 131.1 (Ar), 131.2 (Ar), 133.9 (Ar), 134.4 (Ar), 136.4 (Ar), 139.2 (Ar), 148.6 (Ar), 168.7 (COOH).

2-(*N*-(4-nitrophenyl)sulfamoyl)benzoic acid (24): yellow solid, mp 102-106 °C; ^1H NMR (400 MHz, DMSO-d_6): δ 7.30 (d, J = 9.2 Hz, 2H, Ar), 7.61-7.70 (m, 3H, Ar), 7.91 (d, J = 7.7 Hz, 1H, Ar), 8.13 (d, J = 9.2 Hz, 2H, Ar). ^{13}C NMR (101 MHz, DMSO-d_6): δ 118.9 (2 x Ar), 125.7 (2 x Ar), 128.7 (Ar), 130.1 (Ar), 130.9 (Ar), 133.8 (Ar), 135.1 (Ar), 136.6 (Ar), 143.0 (Ar), 144.9 (Ar), 168.6 (COOH).

2-(*N*-(*p*-tolyl)sulfamoyl)benzoic acid (25): white solid, mp 160-162 °C; ^1H NMR (400 MHz, DMSO-d_6): δ 2.18 (s, 3H, CH_3), 6.98-7.04 (m, 4H, Ar), 7.57-7.62 (m, 1H, Ar), 7.63-7.68 (m, 2H, Ar), 7.75 (d, J = 7.6 Hz, 1H, Ar), 9.70 (s, 1H, SO_2NH, D_2O exch.), 13.64 (br s, 1H, COOH, D_2O exch.). ^{13}C NMR (101 MHz, DMSO-d_6): δ 20.8 (CH_3), 121.3 (2 x Ar), 128.95 (Ar), 129.6 (Ar), 130.01 (2 x Ar), 130.9 (Ar), 133.3 (Ar), 134.1 (Ar), 134.1 (Ar), 135.1 (Ar), 137.1 (Ar), 169.1 (COOH).

2-(*N*-(2,6-dimethylphenyl)sulfamoyl)benzoic acid (26): white solid, mp 176-178 °C; ^1H NMR (400 MHz, DMSO-d_6): δ 2.00 (s, 6H, 2 x CH_3), 7.01-7.03 (m, 2H, Ar), 7.06-7.09 (m, 1H, Ar), 7.63-7.67 (m, 2H, Ar), 7.71-7.79 (m, 2H, Ar), 8.66 (s, 1H, SO_2NH, D_2O exch.), 13.79 (br s, 1H, COOH, D_2O

exch.). ^{13}C NMR (101, MHz, $CDCl_3$): δ 18.9 (2 x CH_3), 127.8 (Ar), 128.5 (Ar), 128.7 (2 x Ar), 130.01 (Ar), 131.5 (Ar), 132.8 (Ar), 133.2 (Ar), 134.1 (Ar). 138.2 (2 x Ar), 140.1 (Ar), 169.5 (COOH).

2-(*N*-(4-isopropylphenyl)sulfamoyl)benzoic acid (27): white solid, mp 80-84 °C; ^{1}H NMR (400 MHz, DMSO-d_6): δ 1.12 (d, J = 6.9 Hz, 6H, 2 × CH_3), 2.74-2.81 (m, 1H, CH), 7.01 (d, J = 8.5 Hz, 2H, Ar), 7.10 (d, J = 8.5 Hz, 2H, Ar), 7.54-7.58 (m, 1H, Ar), 7.62-7.69 (m, 2H, Ar), 7.76 (d, J = 7.8 Hz, 1H, Ar), 10.13 (br s, 1H, SO_2NH, D_2O exch.). ^{13}C NMR (101 MHz, DMSO-d_6): δ 24.2 (2 × CH_3), 33.2 (CH), 121.2 (2 × Ar), 127.3 (2 × Ar), 128.7 (Ar), 129.8 (Ar), 130.6 (Ar), 133.2 (Ar), 135.0 (Ar), 135.6 (Ar), 137.2 (Ar), 144.9 (Ar), 169.2 (COOH).

2-(*N*-(4-methoxyphenyl)sulfamoyl)benzoic acid (28): white solid, mp 152-156 °C; ^{1}H NMR (400 MHz, DMSO-d_6): δ 3.67 (s, 3H, CH_3), 6.80 (d, J = 9.0 Hz, 2H, Ar), 7.01 (d, J = 9.0 Hz, 2H, Ar), 7.56-7.62 (m, 1H, Ar), 7.64-7.72 (m, 3H, Ar), 9.46 (br s, 1H, SO_2NH, D_2O exch.), 13.68 (br s, 1H, COOH, D_2O exch.). ^{13}C NMR (101 MHz, DMSO-d_6): δ 55.6 (CH_3), 114.7 (2 x Ar), 124.1 (2 x Ar), 129.1 (Ar), 129.7 (Ar), 130.2 (Ar), 130.9 (Ar), 133.3 (Ar), 133.9 (Ar), 137.1 (Ar), 157.2 (Ar), 169.2 (COOH).

Synthesis and characterization of derivatives 29-40

Triethylamine (3 equiv.) and ethyl chloroformate (1.2 equiv.) were added to a stirring solution of the proper acid derivative (1.0 equiv.) in tetrahydrofuran and stirred under nitrogen atmosphere for 1 h at room temperature. Once the reaction completion, the mixture was extracted with ethyl acetate (3 × 20 mL) and 2N HCl (1 × 10 mL). The organics were reunited, dried over sodium sulfate and concentrated *in vacuo*. Purification through column chromatography on silica gel, using the proper mixtures of solvents, gave compounds **29-40** as white or yellow solids.

2-phenylbenzo[*d*]isothiazol-3(2*H*)-one 1,1-dioxide (29): white solid, mp 188-190 °C; ^{1}H NMR (400 MHz, $CDCl_3$): δ 7.54-7.61 (m, 5H, Ar), 7.89-7.98 (m, 2H, Ar), 8.02-8.04 (m, 1H, Ar), 8.18-8.20 (m, 1H, Ar). ^{13}C NMR (101 MHz, $CDCl_3$): δ 121.3 (Ar), 125.7 (Ar), 127.2 (Ar), 128.7 (Ar), 128.8 (2 × Ar), 129.9 (2 × Ar), 130.1 (Ar), 134.5 (Ar), 135.1 (Ar), 137.6 (Ar), 158.4 (C=O).

2-(4-fluorophenyl)benzo[*d*]isothiazol-3(2*H*)-one 1,1-dioxide (30): white solid, mp 163-165 °C; ^{1}H NMR (400 MHz, $CDCl_3$): δ 7.25-7.31 (m, 2H, Ar), 7.53-7.58 (m, 2H, Ar), 7.91-7.95 (m, 1H, Ar), 7.96-8.00 (m, 1H, Ar), 8.03-8.05 (m, 1H, Ar), 8.19-8.21 (m, 1H, Ar). ^{13}C NMR (101 MHz, $CDCl_3$): δ 117.1 (d, $^{2}J_{C\text{-}F}$ = 23.1 Hz, 2 × Ar), 121.3 (Ar), 124.4 (d, $^{4}J_{C\text{-}F}$ = 23.1 Hz, Ar), 125.7 (Ar), 127.1 (Ar), 131.0 (d, $^{3}J_{C\text{-}F}$ = 9.1 Hz, 2 × Ar), 134.6 (Ar), 135.2 (Ar), 137.6 (Ar), 131.5 (Ar), 132.5 (d, J = 3.03 Hz, 1 × Ar), 132.7 (Ar), 137.7 (Ar), 158.4 (C=O), 163.5 (d, $^{1}J_{C\text{-}F}$ = 250.9, C-F).

2-(2-chlorophenyl)benzo[*d*]isothiazol-3(2*H*)-one 1,1-dioxide (31): white solid, mp 208-210 °C; ^{1}H NMR (400 MHz, $CDCl_3$): δ 7.46-7.50 (m, 1H, Ar), 7.52-7.57 (m, 1H, Ar), 7.62-7.67 (m, 2H, Ar), 7.91-7.99 (m, 2H, Ar), 8.03-8.05 (m, 1H, Ar), 8.20-8.22 (m, 1H, Ar). ^{13}C NMR (101 MHz, $CDCl_3$): δ 121.4 (Ar), 125.9 (Ar), 126.2 (Ar), 126.9 (Ar), 128.1 (Ar), 131.1 (Ar), 132.0 (Ar), 132.0 (Ar), 134.5 (Ar), 135.2 (Ar), 135.3 (Ar), 138.1 (Ar), 157.5 (C=O).

2-(4-chlorophenyl)benzo[*d*]isothiazol-3(2*H*)-one 1,1-dioxide (32): white solid, mp 156-158 °C; ^{1}H NMR (400 MHz, $CDCl_3$): δ 7.50-7.57 (m, 4H, Ar), 7.91-7.95 (m, 1H, Ar), 7.95-7.99 (m, 1H, Ar), 8.0.3 (d, J = 7.3 Hz, 1H, Ar), 8.19 (d, J = 7.8 Hz, 1H, Ar). ^{13}C NMR (101 MHz, $CDCl_3$): δ 121.3 (Ar), 125.7 (Ar), 127.0 (Ar), 127.3 (Ar), 129.9 (2 × Ar), 130.2 (2 × Ar), 134.6 (Ar), 135.3 (Ar), 136.3 (Ar), 137.5 (Ar), 158.2 (C=O).

2-(4-bromophenyl)benzo[*d*]isothiazol-3(2*H*)-one 1,1-dioxide (33): white solid, mp 141-143 °C; ^{1}H NMR (400 MHz, $CDCl_3$): δ 7.43-7.47 (m, 2H, Ar), 7.69-7.72 (m, 2H, Ar), 7.90-7.98 (m, 2H, Ar), 8.01-8.03 (m, 1H, Ar), 8.18 (d, J = 7.4 Hz, 1H, Ar). ^{13}C NMR (101 MHz, $CDCl_3$): δ 121.3 (2 x Ar), 124.4 (Ar), 125.8 (Ar), 127.0 (Ar), 127.8 (Ar), 130.1 (2 × Ar), 133.2 (2 × Ar), 134.6 (Ar), 135.3, (Ar), 137.5 (Ar), 158.2 (C=O).

2-(4-(trifluoromethyl)phenyl)benzo[*d*]isothiazol-3(2*H*)-one 1,1-dioxide (34): white solid, mp 158-160 °C; ^{1}H NMR (400 MHz, $CDCl_3$): δ 7.75 (d, J = 8.4 Hz, 2H, Ar), 7.85 (d, J = 8.4 Hz, 2H, Ar), 7.92-8.01 (m, 2H, Ar), 8.03-8.06 (m, 1H, Ar), 8.20-8.22 (m, 1H, Ar). ^{13}C NMR (101 MHz, DMSO-d_6): δ 121.3 (Ar), 123.6 (d, $^{1}J_{C\text{-}F}$ = 272.6 Hz, CF_3), 125.9 (Ar), 126.9 (Ar), 127.0 (q, $^{3}J_{C\text{-}F}$ =

3.7 Hz, 2 x Ar), 128.4 (2 × Ar), 131.9 (m, Ar), 132.5 (Ar), 134.7 (Ar), 135.4 (Ar), 137.5 (Ar), 158.1 (C=O).

2-(3-nitrophenyl)benzo[*d*]isothiazol-3(2*H*)-one 1,1-dioxide (35): yellow solid, mp 136-138 °C; ^{1}H NMR (400 MHz, $CDCl_3$): δ 7.78 (t, *J* = 8.2 Hz, 1H, Ar), 7.94-7.98 (m, 2H, Ar), 7.99-8.03 (m, 1H, Ar), 8.04-8.06 (m, 1H, Ar), 8.22 (d, *J* = 7.2 Hz, 1H, Ar) 8.40-8.43 (m, 1H, Ar), 8.49 (t, *J* = 2.0 Hz, 1H, Ar). ^{13}C NMR (101 MHz, $CDCl_3$): δ 121.4 (Ar), 123.6 (Ar), 124.6 (Ar), 126.0 (Ar), 126.7 (Ar), 130.4 (Ar), 130.8 (Ar), 134.0 (Ar), 134.9 (Ar), 135.6 (Ar), 137.4 (Ar), 149.0 (Ar), 158.1 (C=O).

2-(4-nitrophenyl)benzo[*d*]isothiazol-3(2*H*)-one 1,1-dioxide (36): yellow solid, mp 232-234 °C; ^{1}H NMR (400 MHz, $CDCl_3$): δ 7.92 (d, *J* = 9.0 Hz, 2H, Ar), 8.09 (t, *J* = 7.2 Hz, 1H, Ar), 8.15 (t, *J* = 7.1 Hz, 1H, Ar), 8.24 (d, *J* = 7.5 Hz, 1H, Ar), 8.44-8.50 (m, 3H, Ar). ^{13}C NMR (101 MHz, $CDCl_3$): δ 121.4 (Ar), 125.1 (2 × Ar), 126.0 (Ar), 126.7 (Ar), 128.0 (2 × Ar), 134.9 (Ar), 135.3 (Ar), 135.7 (Ar), 137.3 (Ar), 147.9 (Ar), 157.9 (C=O).

2-(*p*-tolyl)benzo[*d*]isothiazol-3(2*H*)-one 1,1-dioxide (37): white solid, mp 172-174 °C; ^{1}H NMR (400 MHz, $CDCl_3$): δ 2.46 (s, 3H, CH_3), 7.38 (d, *J* = 8.3 Hz, 2H, Ar), 7.43-7.45 (m, 2H, Ar), 7.88-7.96 (m, 2H, Ar), 8.01-8.03 (m, 1H, Ar), 8.16-8.18 (m, *J* = 8.0 Hz, 1H, Ar). ^{13}C NMR (101 MHz, $CDCl_3$): δ 21.4 (CH_3), 121.3 (Ar), 125.6 (Ar), 125.8 (Ar), 127.3 (Ar), 128.7 (2 x Ar), 130.6 (2 x Ar), 134.4 (Ar), 135.03 (Ar), 137.7 (Ar), 140.5 (Ar), 158.5 (C=O).

2-(2,6-dimethylphenyl)benzo[*d*]isothiazol-3(2*H*)-one 1,1-dioxide (38): white solid, mp 224-228 °C; ^{1}H NMR (400 MHz, $CDCl_3$): δ 2.36 (s, 6H, 2 x CH_3), 7.24 (d, *J* = 7.6 Hz, 2H, Ar), 7.32-7.36 (m, 1H, Ar), 7.90-7.98 (m, 2H, Ar), 8.02-8.04 (m, 1H, Ar), 8.19-8.21 (m, 1H, Ar). ^{13}C NMR (101 MHz, $CDCl_3$): δ 18.5 (2 x CH_3), 121.2 (Ar), 125.7 (Ar), 126.3 (Ar), 127.0 (Ar), 129.2 (2 x Ar), 130.6 (Ar), 134.4 (Ar), 135.1 (Ar), 138.4 (Ar), 139.9 (Ar), 158.2 (C=O).

2-(4-isopropylphenyl)benzo[*d*]isothiazol-3(2*H*)-one 1,1-dioxide (39): white solid, mp 138-140 °C; ^{1}H NMR (400 MHz, $CDCl_3$): δ 1.32 (d, *J* = 6.9 Hz, 6H, 2 x CH_3), 2.96-3.07 (m, 1H, CH), 7.42-7.48 (m, 4H, Ar), 7.88-7.96 (m, 2H, Ar), 8.02 (d, *J* = 7.2 Hz, 1H, Ar), 8.17-8.19 (m, 1H, Ar). ^{13}C NMR

(101 MHz, $CDCl_3$): δ 23.8 (2 x CH_3), 34.0 (CH), 121.3 (Ar), 125.6 (Ar), 126.0 (Ar), 127.3 (Ar), 128.1 (2 x Ar), 128.7 (2 x Ar), 134.4 (Ar), 135.03 (Ar), 137.7 (Ar), 151.2 (Ar), 158.5 (C=O).

2-(4-methoxyphenyl)benzo[*d*]isothiazol-3(2*H*)-one 1,1-dioxide (40): white solid, mp 192-196 °C; ^{1}H NMR (400 MHz, $CDCl_3$): δ 3.89 (s, 3H, OCH_3), 7.06-7.10 (m, 2H, Ar), 7.44-7.48 (m, 2H, Ar), 7.88-7.97 (m, 2H, Ar), 8.01-8.03 (m, 1H, Ar), 8.17-8.19 (m, 1H, Ar). ^{13}C NMR (101 MHz, $CDCl_3$): δ 55.6 (CH_3), 115.3 (2 x Ar), 120.5 (Ar), 121.3 (Ar), 125.6 (Ar), 127.3 (Ar), 130.5 (2 x Ar), 134.4 (Ar), 135.01 (Ar), 137.7 (Ar), 158.7 (Ar), 161.01 (C=O).

Synthesis and characterization of derivatives 41-46

Propargylated saccharin (1.0 equiv.) and the proper benzyl azide (1.05 equiv.) were added to a stirring solution of CuI (0.02 equiv.), DIPEA (0.04 equiv.) and CH_3COOH (0.04 equiv.) in dichloromethane and stirred at room temperature until the alkyne disappeared. The mixture was then extracted with dichloromethane (3 × 20 mL) and H_2O. The organics were reunited, dried over sodium sulfate and concentrated *in vacuo*. Purification through column chromatography on silica gel, using the proper solvents mixtures, gave compounds **STR1-STR6** as white solids.

2-((1-phenyl-1*H*-1,2,3-triazol-4-yl)methyl)benzo[*d*]isothiazol-3(2*H*)-one 1,1-dioxide (41): white solid, 134-136 °C; ^{1}H NMR (400 MHz, $CDCl_3$): δ 5.18 (s, 2H, CH_2), 7.41-7.45 (m, 1H, Ar), 7.49-7.52 (m, 2H, Ar), 7.71-7.73 (m, 2H, Ar), 7.84-7.93 (m, 2H, Ar), 7.96 (d, J = 7.2 Hz, 1H, Ar), 8.08-8.10 (m, 1H, Ar), 8.13 (s, 1H, Ar-triazole). ^{13}C NMR (101 MHz, $CDCl_3$): δ 34.0 (CH_2), 120.7 (2 x Ar), 121.1 (Ar), 121.7 (Ar-triazole), 125.4 (Ar), 127.1 (Ar), 128.9 (Ar), 129.7 (2 x Ar), 134.5 (Ar), 135.1 (Ar), 136.9 (Ar-triazole), 137.7 (Ar), 142.5 (Ar) 158.6 (C=O).

2-((1-(4-chlorophenyl)-1*H*-1,2,3-triazol-4-yl)methyl)benzo[*d*]isothiazol-3(2*H*)-one 1,1-dioxide (42): white solid, 196-198 °C; ^{1}H NMR (400 MHz, $CDCl_3$): δ 5.19 (s, 2H, CH_2), 7.50 (d, J = 8.8 Hz, 2H, Ar), 7.69 (d, J = 8.8 Hz, 2H, Ar), 7.86-7.94 (m, 2H, Ar), 7.98 (d, J = 7.3 Hz, 1H, Ar), 8.11-8.12 (m, 2H, Ar-triazole + Ar). ^{13}C NMR (101 MHz, $CDCl_3$): δ 34.9 (CH_2), 121.1 (Ar), 121.6 (Ar-triazole),

121.8 (2 x Ar), 125.4 (Ar), 127.1 (Ar), 129.9 (2 x Ar), 134.4 (Ar), 134.7 (Ar), 135.1 (Ar), 135.4 (Ar-triazole), 137.7 (Ar), 142.8 (Ar), 158.6 (C=O).

2-((1-(4-bromophenyl)-1*H*-1,2,3-triazol-4-yl)methyl)benzo[d]isothiazol-3(2*H*)-one 1,1-dioxide (43): white solid, 184-188 °C; ^{1}H NMR (400 MHz, DMSO-d_6): δ 5.10 (s, 2H, CH_2), 7.77-7.81 (m, 2H, Ar), 7.86-7.90 (m, 2H, Ar), 8.01-8.05 (m, 1H, Ar), 8.06-8.10 (m, 2H, Ar), 8.15-8.17 (m, 1H, Ar), 8.88 (s, 1H, Ar-triazole). ^{13}C NMR (101 MHz, DMSO-d_6): δ 33.9 (CH_2), 121.9 (Ar-triazole), 122.1 (Ar), 122.4 (2 x Ar), 122.7 (Ar), 125.7 (Ar), 126.8 (Ar), 133.3 (2 x Ar), 135.8 (Ar), 136.1 (Ar-triazole), 136.4 (Ar), 137.3 (Ar), 143.2 (Ar), 158.8 (C=O).

2-((1-(*p*-tolyl)-1*H*-1,2,3-triazol-4-yl)methyl)benzo[*d*]isothiazol-3(2*H*)-one 1,1-dioxide (44): white solid, 172-174 °C; ^{1}H NMR (400 MHz, $CDCl_3$): δ 2.32 (s, 3H, CH_3), 5.08 (s, 2H, CH_2), 7.20 (d, J = 8.6 Hz, 2H, Ar), 7.50 (d, J = 8.5 Hz, 2H, Ar), 7.74-7.88 (m, 3H, Ar), 7.99-8.01 (m, 2H, Ar-triazole + Ar). ^{13}C NMR (101 MHz, $CDCl_3$): δ 21.1 (CH_3), 34.1 (CH_2), 120.6 (2 x Ar), 121.1 (Ar), 121.6 (Ar-triazole), 125.4 (Ar), 127.2 (Ar), 130.2 (2 x Ar), 134.5 (Ar), 134.6 (Ar-triazole), 135.1 (Ar), 137.7 (Ar), 139.0 (Ar), 142.4 (Ar), 158.6 (C=O).

2-((1-(4-methoxyphenyl)-1*H*-1,2,3-triazol-4-yl)methyl)benzo[*d*]isothiazol-3(2*H*)-one 1,1-dioxide (45): white solid, 174-176 °C; ^{1}H NMR (400 MHz, $CDCl_3$): δ 3.86 (s, 3H, OCH_3), 5.17 (s, 2H, CH_2), 6.98-7.02 (m, 2H, Ar), 7.59-7.63 (m, 2H, Ar), 7.84-7.88 (m, 1H, Ar), 7.88-7.92 (m, 2H, Ar), 7.96 (d, J = 7.2 Hz, 1H, Ar), 8.04 (s, 1H, Ar-triazole). ^{13}C NMR (101 MHz, $CDCl_3$): δ 34.1 (OCH_3), 55.6 (CH_2), 114.7 (2 x Ar), 121.1 (Ar), 121.8 (Ar-triazole), 122.3 (2 x Ar), 125.4 (Ar), 127.2 (Ar), 130.3 (Ar-triazole), 134.5 (Ar), 135.1 (Ar), 137.7 (Ar), 142.4 (Ar), 158.6 (Ar), 159.9 (C=O).

2-((1-benzyl-1*H*-1,2,3-triazol-4-yl)methyl)benzo[*d*]isothiazol-3(2*H*)-one 1,1-dioxide (46): white solid, 130-132 °C; ^{1}H NMR (400 MHz, DMSO-d_6): δ 4.99 (s, 2H, CH_2), 5.16 (s, 2H, CH_2), 7.28-7.38 (m, 5H, Ar), 7.99-8.03 (m, 1H, Ar), 8.04-8.08 (m, 1H, Ar), 8.13 (m, 1H, Ar), 8.20 (s, 1H, Ar-triazole), 8.32 (d , J = 7.5 Hz, 1H, Ar). ^{13}C NMR (101 MHz, DMSO-d_6): δ 34.0 (CH_2), 53.3 (CH_2), 122.1 (Ar), 124.7 (Ar), 125.7 (Ar), 126.7 (Ar-triazole), 128.3 (2 x Ar), 128.6 (Ar), 129.2 (2 x Ar), 135.8 (Ar), 136.4 (Ar), 136.4 (Ar-triazole), 137.3 (Ar), 141.9 (Ar), 158.7 (C=O).

Synthesis and characterization of derivatives 47-49

To a stirring solution of the proper saccharin derivative (1 equiv.) in tetrahydrofuran (10 mL) at room temperature were added dropwise 10 mL of aqueous 2N NaOH (5.0 equiv.). After 2 h the organic solvent was evaporated and the basic solution, containing the acid derivative as sodium salt, acidified with 4N HCl. The resulting suspension was filtered and the collected solid washed with *n*-hexane to afford the title compounds as a white solid.

2-(*N*-((1-phenyl-1*H*-1,2,3-triazol-4-yl)methyl)sulfamoyl)benzoic acid (47): white solid, 162-166 °C; ^{1}H NMR (400 MHz, DMSO-d_6): δ 4.29 (d, J = 3.7 Hz, 2H, CH_2), 7.49 (t, J = 7.4 Hz, 1H, Ar), 7.57-7.71 (m, 5H, Ar), 7.76-7.78 (m, 3H, 2 x Ar + SO_2NH, D_2O exch.), 7.88-7.91 (m, 1H, Ar), 8.48 (s, 1H, Ar-triazole), 13.71 (br s, 1H, COOH, D_2O exch.). ^{13}C NMR (101 MHz, DMSO-d_6): δ 38.6 (CH_2), 120.5 (2 x Ar), 122.0 (Ar-triazole), 128.9 (Ar), 129.1 (Ar), 129.5 (Ar), 130.4 (2 x Ar), 131.1 (Ar), 133.0 (Ar), 133.4 (Ar-triazole), 136.9 (Ar-), 138.1 (Ar), 144.8 (Ar), 169.4 (COOH).

2-(*N*-((1-(*p*-tolyl)-1*H*-1,2,3-triazol-4-yl)methyl)sulfamoyl)benzoic acid (48): white solid, 182-186 °C; ^{1}H NMR (400 MHz, DMSO-d_6): δ 2.37 (s, 3H, CH_3), 4.27 (d, J = 5.5 Hz, 2H, CH_2), 7.38 (t, J = 8.3 Hz, 2H, Ar), 7.60-7.69 (m, 5H, Ar), 7.73-7.77 (br s, 1H, SO_2NH, D_2O exch.), 7.83-7.90 (m, 1H, Ar), 8.41 (s, 1H, Ar-triazole), 13.70 (br s, 1H, COOH, D_2O exch.). ^{13}C NMR (101 MHz, DMSO-d_6): δ 21.0 (CH_3), 38.6 (CH_2), 120.4 (2 x Ar), 121.9 (Ar-triazole), 129.0 (Ar), 129.5 (Ar), 130.7 (2 x Ar), 131.1 (Ar), 132.9 (Ar), 133.4 (Ar-triazole), 134.7 (Ar), 138.1 (Ar), 138.8 (Ar), 144.6 (Ar), 169.4 (COOH).

2-(*N*-((1-benzyl-1*H*-1,2,3-triazol-4-yl)methyl)sulfamoyl)benzoic acid (49): white solid, 144-146 °C; ^{1}H NMR (400 MHz, DMSO-d_6): δ 4.15 (s, 2H, CH_2), 5.49 (s, 2H, CH_2), 7.23-7.25 (m, 2H, Ar), 7.31-7.40 (m, 3H, Ar), 7.54-7.59 (m, 1H, Ar), 7.64 (d, J = 4.1 Hz, 2H, Ar), 7.82 (d, J = 7.8 Hz, 1H, Ar), 7.85 (s, 1H, Ar triazole). ^{13}C NMR (101 MHz, DMSO-d_6): 38.7 (CH_2), 53.1 (CH_2), 123.7 (Ar-triazole), 128.4 (2 x Ar), 128.7 (Ar), 128.7 (Ar), 129.2 (2 x Ar), 129.7 (Ar), 130.7 (Ar), 132.9 (Ar), 134.3 (Ar), 136.4 (Ar), 138.0 (Ar-triazole), 144.2 (Ar), 169.4 (COOH).

6-methyl-3-(3-methylbut-2-en-1-yl)-1,2,3-oxathiazin-4(3H)-one 2,2-dioxide (50): 3,3-dimethylallyl bromide (1 equiv.) was added to a stirring solution of acesulfame K (1 equiv.) in *N*,*N*-dimethylformamide and the reaction stirred under nitrogen atmosphere at 0 °C for 48 h. The mixture was poured on ice and the resulting aqueous phase/emulsion extracted with dichloromethane (3 × 20 mL). The organic layers were reunited, dried over sodium sulfate, and concentrated *in vacuo*. Purification through column chromatography on silica gel (*n*-hexane:ethyl acetate, 2:1) gave the title compound as a yellow oil (70% yield); ^{1}H NMR (300 MHz, $CDCl_3$): δ 1.70 (s, 3H, CH_3), 1.73 (s, 3H, CH_3), 2.17 (s, 3H, CH_3) 4.40 (d, J = 6.9 Hz, 2H, CH_2), 5.24 (t, J = 1.2 Hz, 1H, CH=), 5.76 (d, J = 1.2 Hz, 1H, CH=). ^{13}C NMR (76 MHz, $CDCl_3$): δ 17.8 (CH_3), 19.6 (CH_3), 25.7 (CH_3), 41.0 (CH_2), 104.4 (CH=, acesulfame), 117.1 (CH=C), 139.0 (C=CH), 160.0 (C=O), 161.6 ($COSO_2$).

3-benzyl-6-methyl-1,2,3-oxathiazin-4(3*H*)-one 2,2-dioxide (51): benzyl bromide (1 equiv.) was added to a stirring solution of acesulfame K (1 equiv.) in *N*,*N*-dimethylformamide and the reaction stirred under nitrogen atmosphere at 0 °C for 48 h. The mixture was poured on ice and the resulting aqueous phase/emulsion extracted with dichloromethane (3 × 20 mL). The organic layers were reunited, dried over sodium sulfate and concentrated *in vacuo*. Purification through column chromatography on silica gel (*n*-hexane:ethyl acetate, 2:1) gave the title compound as a colourless oil (75% yield); ^{1}H NMR (300 MHz, $CDCl_3$): δ 2.23 (d, J = 0.3 Hz, 3H, CH_3), 5.37 (s, 2H, CH_2), 5.81 (d, J = 0.6 Hz, 1H, CH=), 7.42 (s, 5H, Ar). ^{13}C NMR (101, MHz, $CDCl_3$): δ 20.5 (CH_3), 70.7 (CH_2), 95.7 (CH=, acesulfame), 128.8 (2 x Ar), 128.9 (2 x Ar), 129.2 (Ar), 133.5 (Ar), 168.8 (C=O), 169.1 ($COSO_2$).

3-(3,4-dichlorobenzyl)-6-methyl-1,2,3-oxathiazin-4(3*H*)-one 2,2-dioxide (52): 3,4-dichlorobenzyl bromide (1 equiv.) was added to a stirring solution of acesulfame K (1 equiv.) in *N*,*N*-dimethylformamide and the reaction stirred under nitrogen atmosphere at 0 °C for 48 h. The mixture was poured on ice and the resulting aqueous phase/emulsion extracted with dichloromethane (3 × 20 mL). The organic layers were reunited, dried over sodium sulfate and concentrated *in vacuo*. Purification through column chromatography on silica gel (*n*-hexane:ethyl acetate, 2:1) gave the title

compound as a white solid (75% yield); 93-94 °C; ^{1}H NMR (400 MHz, $CDCl_3$): δ 2.22 (s, 3H, CH_3), 4.92 (s, 2H, CH_2), 5.84 (d, J = 1.2 Hz, 1H, CH=), 7.24-7.27 (m, 1H, Ar), 7.41 (d, J = 8.4 Hz, 1H, Ar), 7.51 (d, J = 2.4 Hz, 1H, Ar). ^{13}C NMR (101 MHz, $CDCl_3$): δ 19.8 (CH_3), 45.0 (CH_2), 104.5 (CH=, acesulfame), 128.2 (2 x Ar), 130.7 (Ar), 130.8 (Ar), 132.8 (Ar), 134.8 (Ar), 160.0 (C=O), 162.1 ($COSO_2$).

Synthesis and characterization of derivatives 53-60

Propargylated acesulfame (1.0 equiv.) and the proper benzyl azide (1.05 equiv.) were added to a stirring solution of CuI (0.02 equiv.), DIPEA (0.04 equiv.) and CH_3COOH (0.04 equiv.) in dichloromethane and stirred at room temperature until the alkyne disappeared. The mixture was extracted with dichloromethane (3 × 20 mL) and H_2O. The organics were reunited, dried over sodium sulfate and concentrated *in vacuo*. Purification through column chromatography on silica gel, using the proper solvents, gave compounds **53-60** as white solids.

6-methyl-3-((1-phenyl-1*H*-1,2,3-triazol-4-yl)methyl)-1,2,3-oxathiazin-4(3*H*)-one 2,2-dioxide (53): white solid, 140-142 °C; ^{1}H NMR (400 MHz, $CDCl_3$): δ 2.15 (s, 3H, CH_3), 5.15 (s, 2H, CH_2), 5.78 (s, 1H, CH=), 7.34-7.38 (m, 1H, Ar), 7.44 (t, J = 7.7 Hz, 2H, Ar), 7.64 (d, J = 7.7 Hz, 2H, Ar), 7.97 (s, 1H, Ar-triazole). ^{13}C NMR (101 MHz, $CDCl_3$): δ 19.8 (CH_3), 37.8 (CH_2), 104.4 (CH=, acesulfame), 120.7 (2 x Ar), 121.8 (Ar-triazole), 128.9 (Ar), 129.8 (2 x Ar), 136.9 (Ar-triazole), 142.5 (Ar), 160.0 (C=O), 162.3 ($COSO_2$).

3-((1-(4-chlorophenyl)-1*H*-1,2,3-triazol-4-yl)methyl)-6-methyl-1,2,3-oxathiazin-4(3*H*)-one 2,2-dioxide (54): white solid, 154-158 °C; ^{1}H NMR (400 MHz, $CDCl_3$): δ 2.16 (d, J = 0.9 Hz, 3H, CH_3), 5.14 (s, 2H, CH_2), 5.78 (d, J = 0.9 Hz, 1H, CH=), 7.40-7.43 (m, 2H, Ar), 7.58-7.62 (m, 2H, Ar), 7.96 (s, 1H, Ar-triazole). ^{13}C NMR (101 MHz, $CDCl_3$): δ 19.8 (CH_3), 37.8 (CH_2), 104.4 (CH=, acesulfame), 121.7 (Ar-triazole), 121.8 (2 x Ar), 129.9 (2 x Ar), 134.7 (Ar), 135.3 (Ar-triazole), 142.7 (Ar), 160.0 (C=O), 162.3 ($COSO_2$).

6-methyl-4-((1-phenyl-1*H*-1,2,3-triazol-4-yl)methoxy)-1,2,3-oxathiazine 2,2-dioxide (55): white solid, 118-120 °C; ^{1}H NMR (400 MHz, $CDCl_3$): δ 2.24 (d, J = 0.7 Hz, 3H, CH_3), 5.60 (s, 2H, CH_2), 5.81 (d, J = 0.9 Hz, 1H, CH=), 7.46-7.50 (m, 1H, Ar), 7.54-7.57 (m, 2H, Ar), 7.73-7.76 (m, 2H, Ar), 8.21 (s, 1H, Ar-triazole). ^{13}C NMR (101 MHz, $CDCl_3$): δ 20.6 (CH_3), 61.3 (CH_2), 95.5 (CH=, acesulfame), 120.7 (2 x Ar), 123.3 (Ar-triazole), 129.2 (Ar), 129.9 (2 x Ar), 136.7 (Ar), 141.3 (Ar-triazole), 168.9 (C=N), 169.2 ($COSO_2$).

6-methyl-4-((1-(*p*-tolyl)-1*H*-1,2,3-triazol-4-yl)methoxy)-1,2,3-oxathiazine 2,2-dioxide (56): white solid, 144-148 °C; ^{1}H NMR (400 MHz, $CDCl_3$): δ 2.24 (d, J = 0.7 Hz, 3H, CH_3), 2.44 (s, 3H, CH_3), 5.59 (s, 2H, CH_2), 5.81 (d, J = 0.7 Hz, 1H, CH=), 7.34 (d, J = 8.0 Hz, 2H, Ar), 7.61 (d, J = 8.4 Hz, 2H, Ar), 8.16 (s, 1H, Ar-triazole). ^{13}C NMR (101 MHz, $CDCl_3$): δ 20.6 (CH_3), 21.3 (CH_3), 61.4 (CH_2), 95.5 (CH=, acesulfame), 120.6 (2 x Ar), 123.2 (Ar-triazole), 130.4 (2 x Ar), 134.4 (Ar), 139.4 (Ar), 141.1 (Ar-triazole), 168.9 (C=N), 169.2 ($COSO_2$).

4-((1-(4-chlorophenyl)-1*H*-1,2,3-triazol-4-yl)methoxy)-6-methyl-1,2,3-oxathiazine 2,2-dioxide (57): white solid, 152-154 °C; ^{1}H NMR (400 MHz, $CDCl_3$): δ 2.24 (s, 3H, CH_3), 5.60 (s, 2H, CH_2), 5.81 (s, 1H, CH=), 7.52-7.55 (m, 2H, Ar), 7.69-7.72 (m, 2H, Ar), 8.20 (s, 1H, Ar-triazole). ^{13}C NMR (101 MHz, $CDCl_3$): δ 20.6 (CH_3), 61.2 (CH_2), 95.5 (CH=, acesulfame), 121.9 (2 x Ar), 123.2 (Ar-triazole), 130.1 (2 x Ar), 135.0 (2 x Ar), 135.1 (Ar), 141.5 (Ar-triazole), 168.8 (C=N), 169.3 ($COSO_2$).

4-((1-(4-bromophenyl)-1*H*-1,2,3-triazol-4-yl)methoxy)-6-methyl-1,2,3-oxathiazine 2,2-dioxide (58): white solid, 168-170 °C; ^{1}H NMR (400 MHz, $CDCl_3$): δ 2.25 (d, J = 0.7 Hz, 3H, CH_3), 5.60 (s, 2H, CH_2), 5.81 (d, J = 0.8 Hz, 1H, CH=), 7.63-7.66 (m, 2H, Ar), 7.68-7.71 (m, 2H, Ar), 8.20 (s, 1H, Ar-triazole). ^{13}C NMR (101 MHz, $CDCl_3$): δ 20.6 (CH_3), 61.2 (CH_2), 95.5 (CH=, acesulfame), 122.1 (2 x Ar), 123.0 (Ar-triazole), 123.2 (Ar), 133.1 (2 x Ar), 135.6 (Ar), 141.6 (Ar-triazole), 168.8 (C=N), 169.3 ($COSO_2$).

4-((1-(4-methoxyphenyl)-1*H*-1,2,3-triazol-4-yl)methoxy)-6-methyl-1,2,3-oxathiazine 2,2-dioxide (59): white solid, 138-140 °C; ^{1}H NMR (400 MHz, $CDCl_3$): δ 2.24 (d, J = 0.7 Hz, 3H, OCH_3), 3.88 (s, 3H, CH_3) 5.85 (s, 2H, CH_2), 5.81 (d, J = 0.8 Hz, 1H, CH=), 7.03-7.05 (m, 2H, Ar), 7.62-7.65

(m, 2H, Ar), 8.12 (s, 1H, Ar-triazole). ^{13}C NMR (101, MHz, $CDCl_3$): δ 20.6 (CH_3), 55.7 (CH_3), 61.4 (CH_2), 95.5 (CH=, acesulfame), 114.9 (2 x Ar), 122.4 (2 x Ar), 123.4 (Ar-triazole), 130.1 (Ar), 135.6 (Ar), 141.0 (Ar-triazole), 168.9 (C=N), 169.2 ($COSO_2$).

4-((1-benzyl-1*H*-1,2,3-triazol-4-yl)methoxy)-6-methyl-1,2,3-oxathiazine 2,2-dioxide (60): white solid, 142-144 °C; ^{1}H NMR (400 MHz, $CDCl_3$): δ 2.13 (s, 3H, CH_3), 5.39 (s, 2H, CH_2), 5.47 (s, 2H, CH_2), 5.68 (s, 1H, CH=), 7.21-7.23 (m, 2H, Ar), 7.28-7.35 (m, 3H, Ar), 7.59 (s, 1H, Ar-triazole). ^{13}C NMR (101 MHz, $CDCl_3$): δ 20.6 (CH_3), 54.4 (CH_3), 61.4 (CH_2), 95.5 (CH=, acesulfame), 124.8 (Ar-triazole), 128.2 (2 x Ar), 129.0 (Ar), 129.3 (2 x Ar), 134.1 (Ar), 140.9 (Ar-triazole), 168.8 (C=N), 169.1 ($COSO_2$).

^{1}H-NMR of compound 6

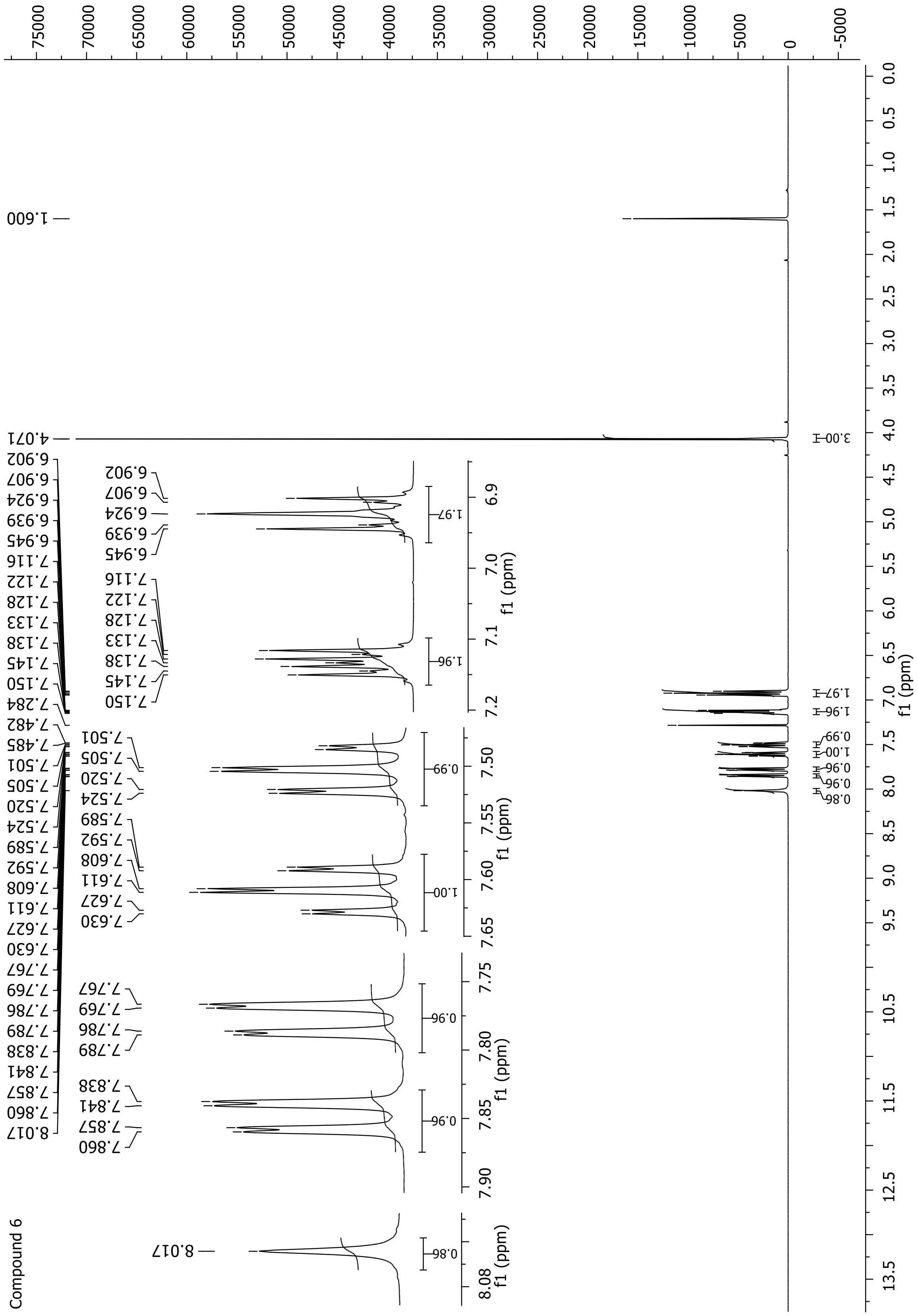

^{13}C-NMR of compound 6

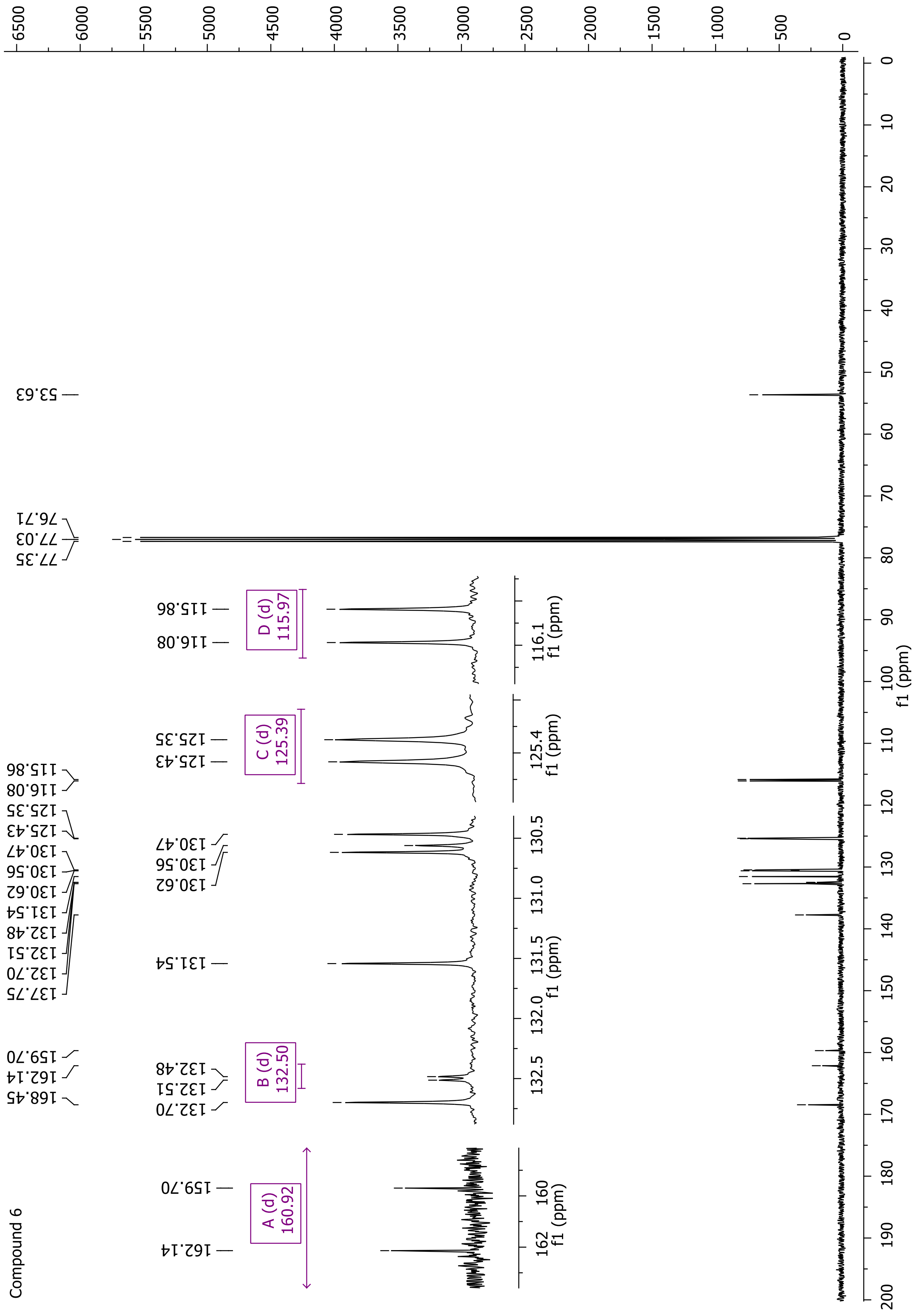

^{1}H-NMR compound 8

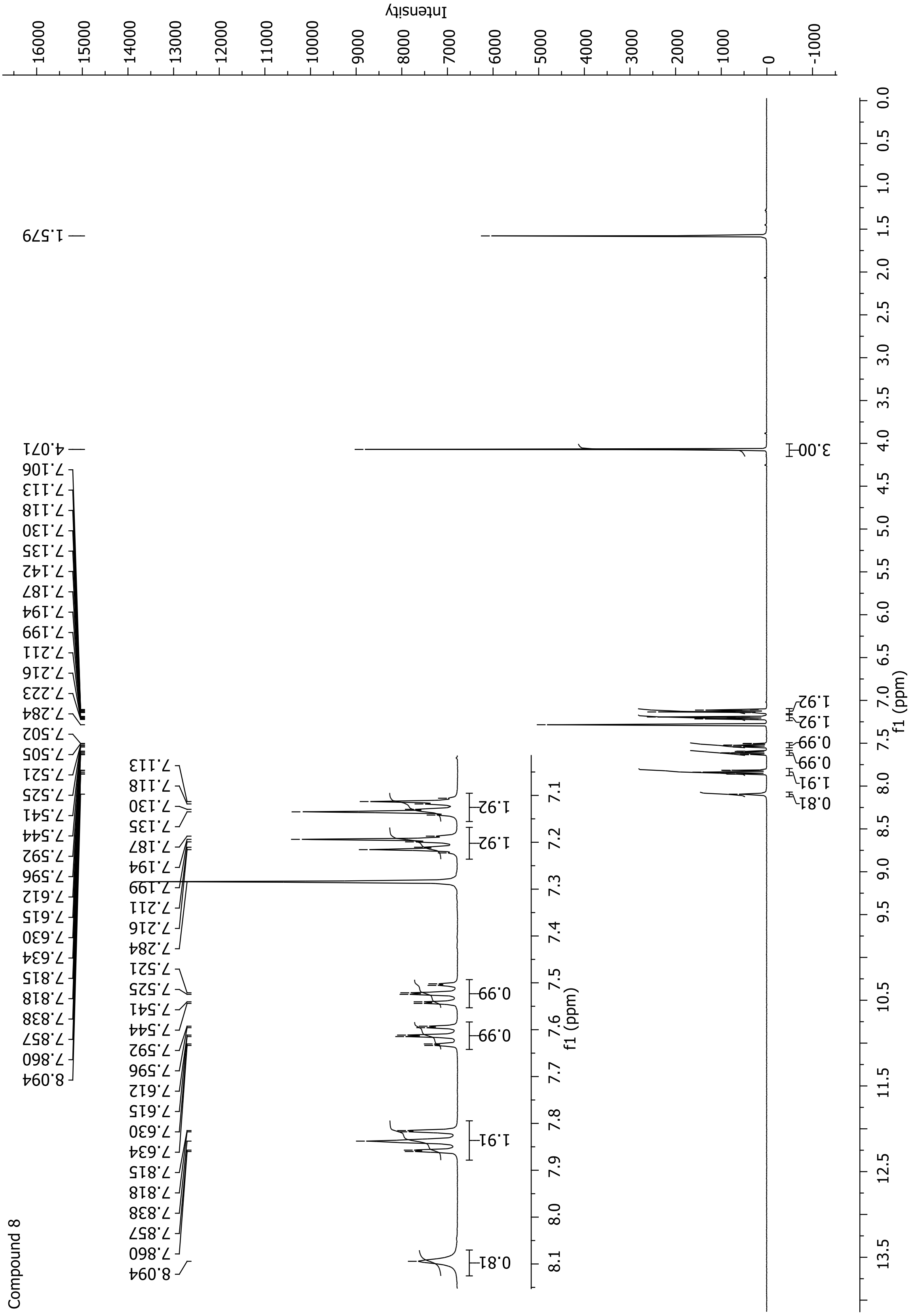

^{1}H-NMR compound 9

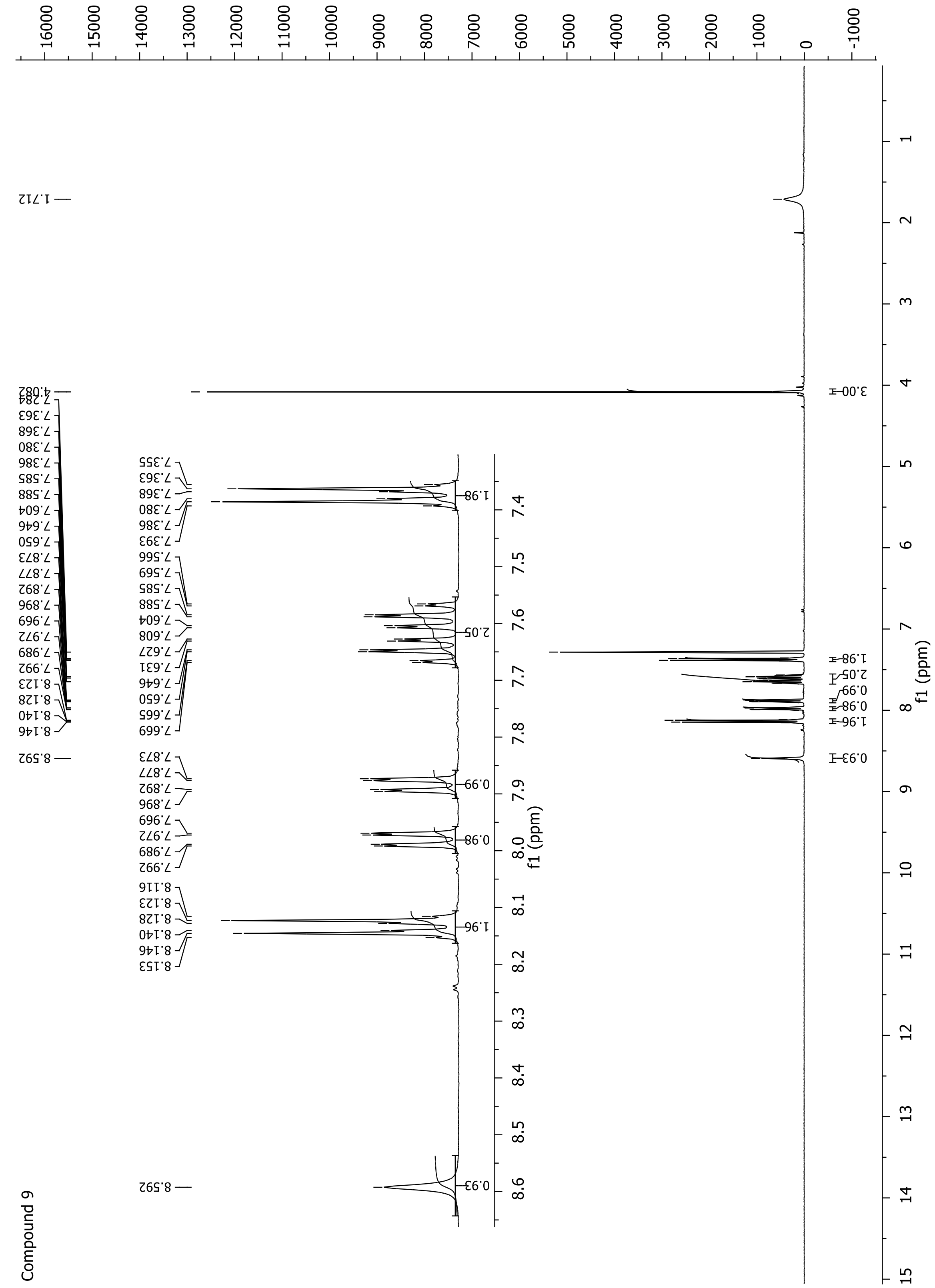

^{13}C-NMR of compound 9

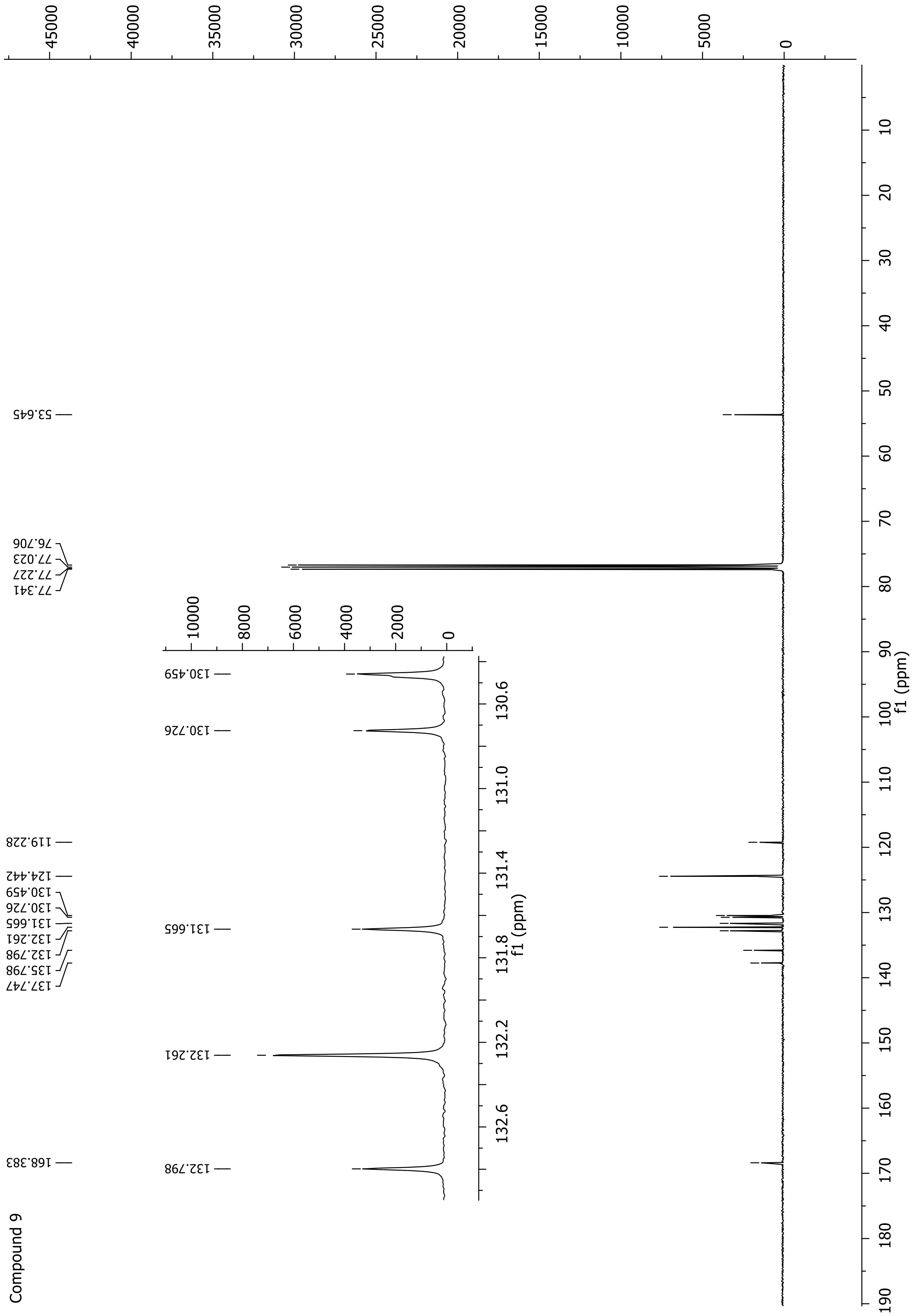

^{1}H-NMR of compound 10

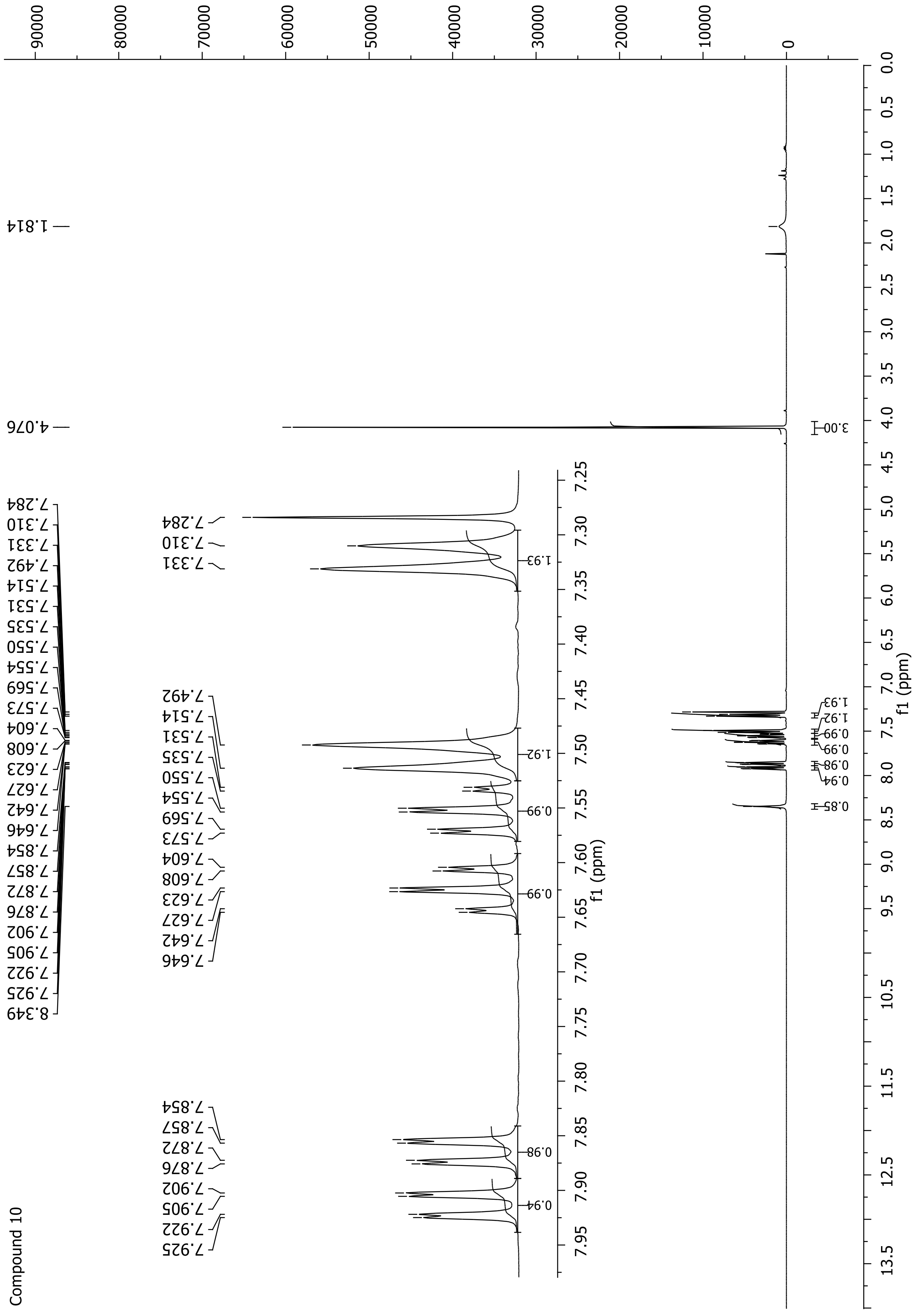

^{13}C-NMR of compound 10

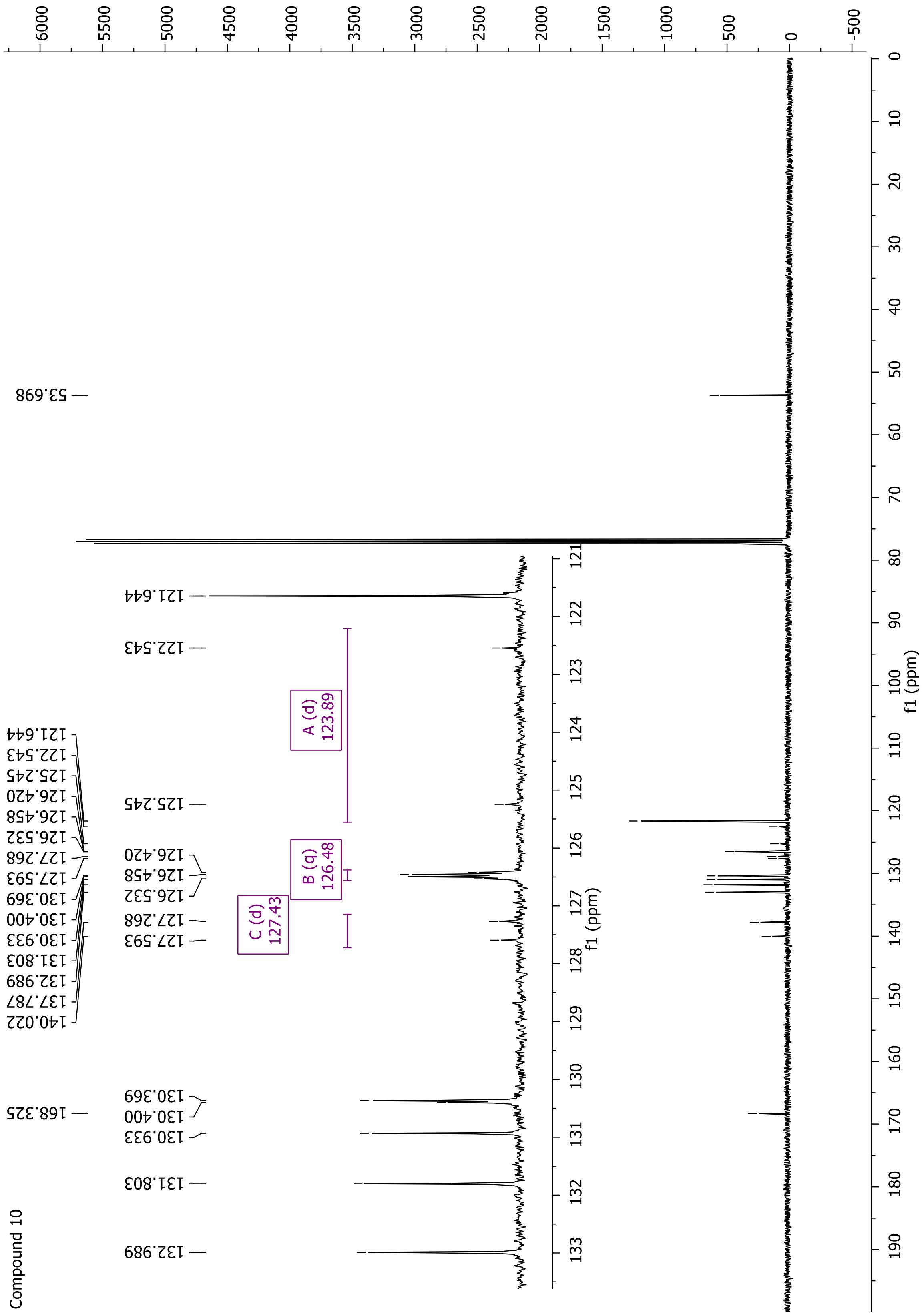

^{1}H-NMR of compound 11

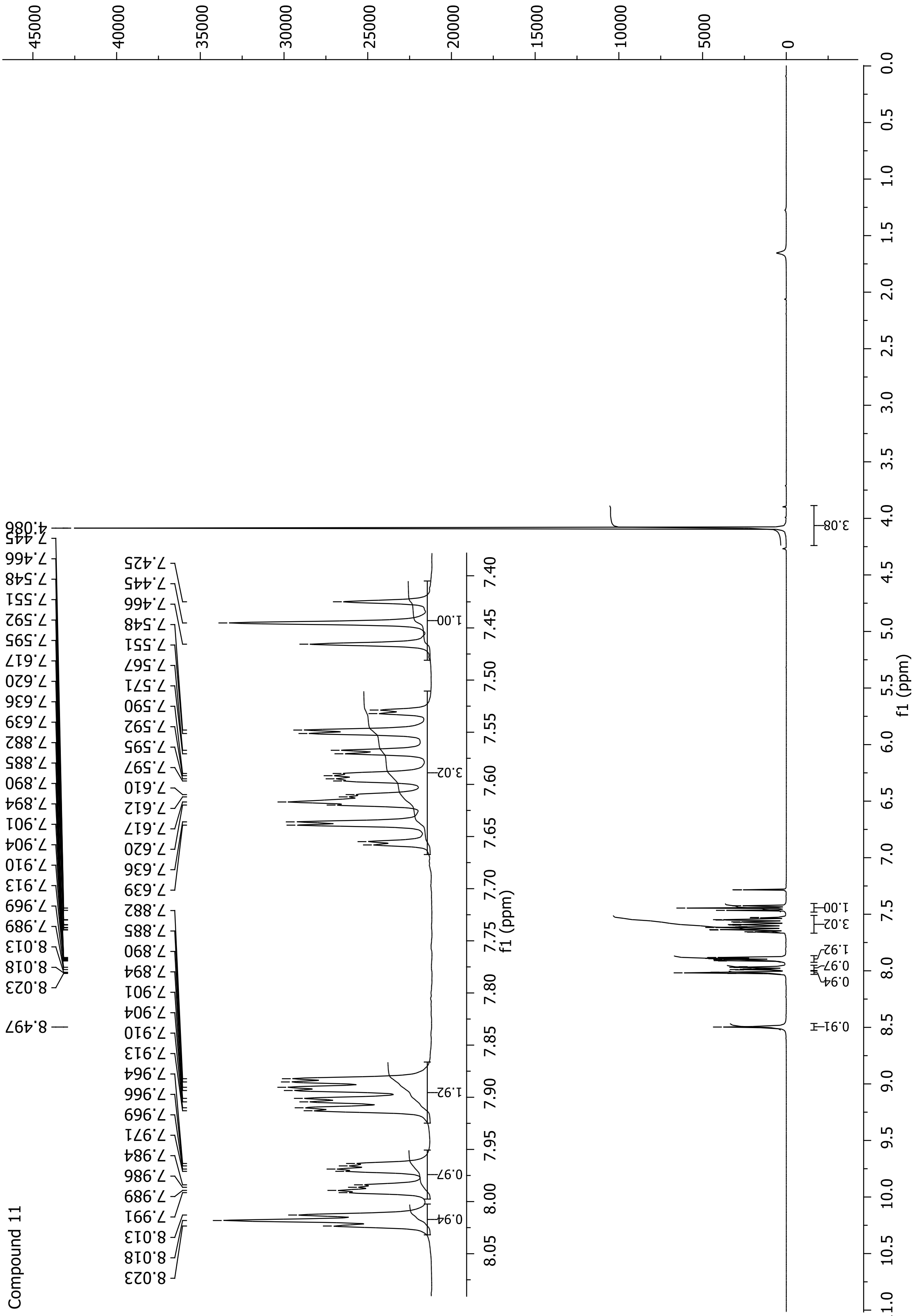

^{13}C-NMR of compound 11

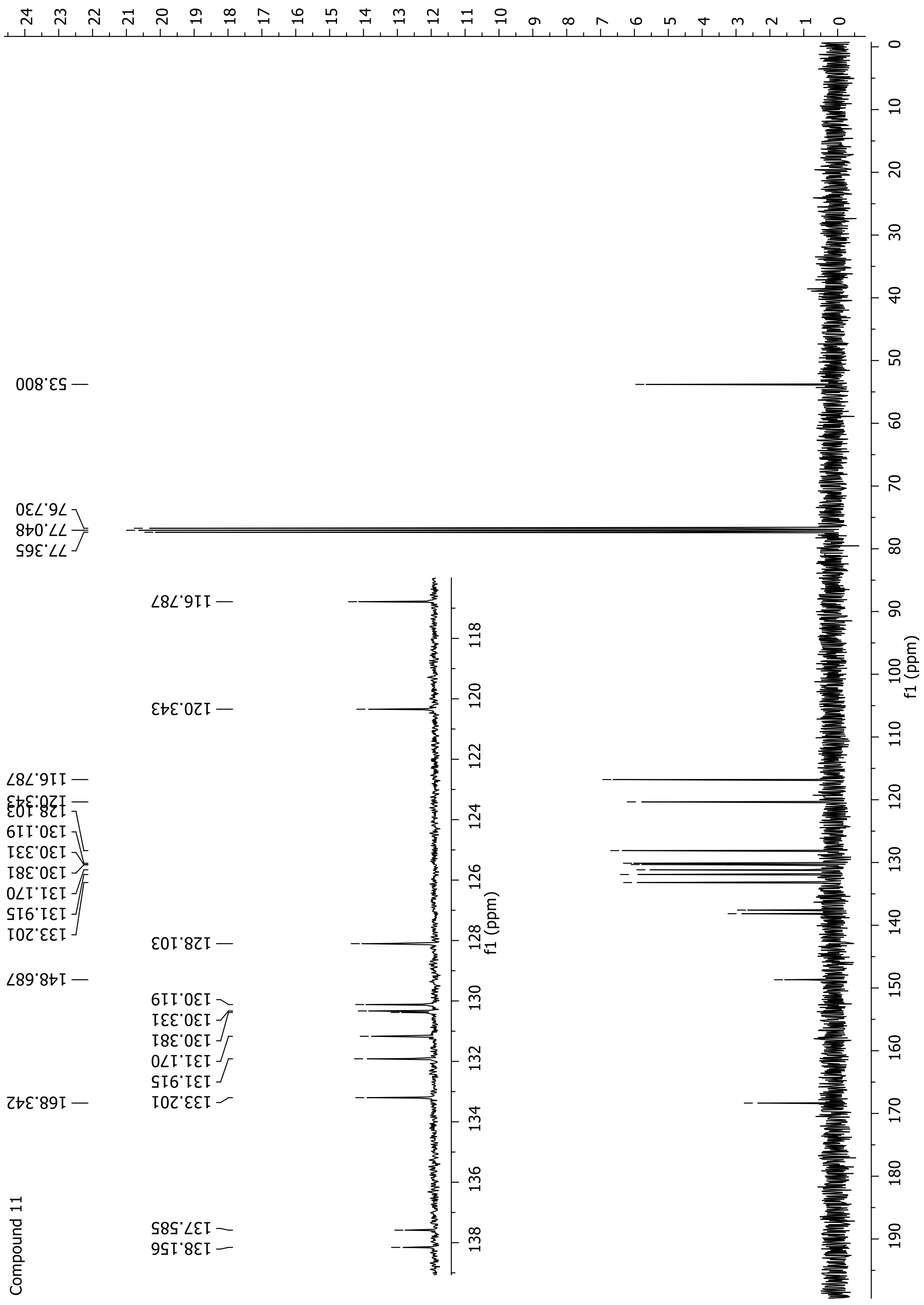

^{13}C-NMR of compound 12

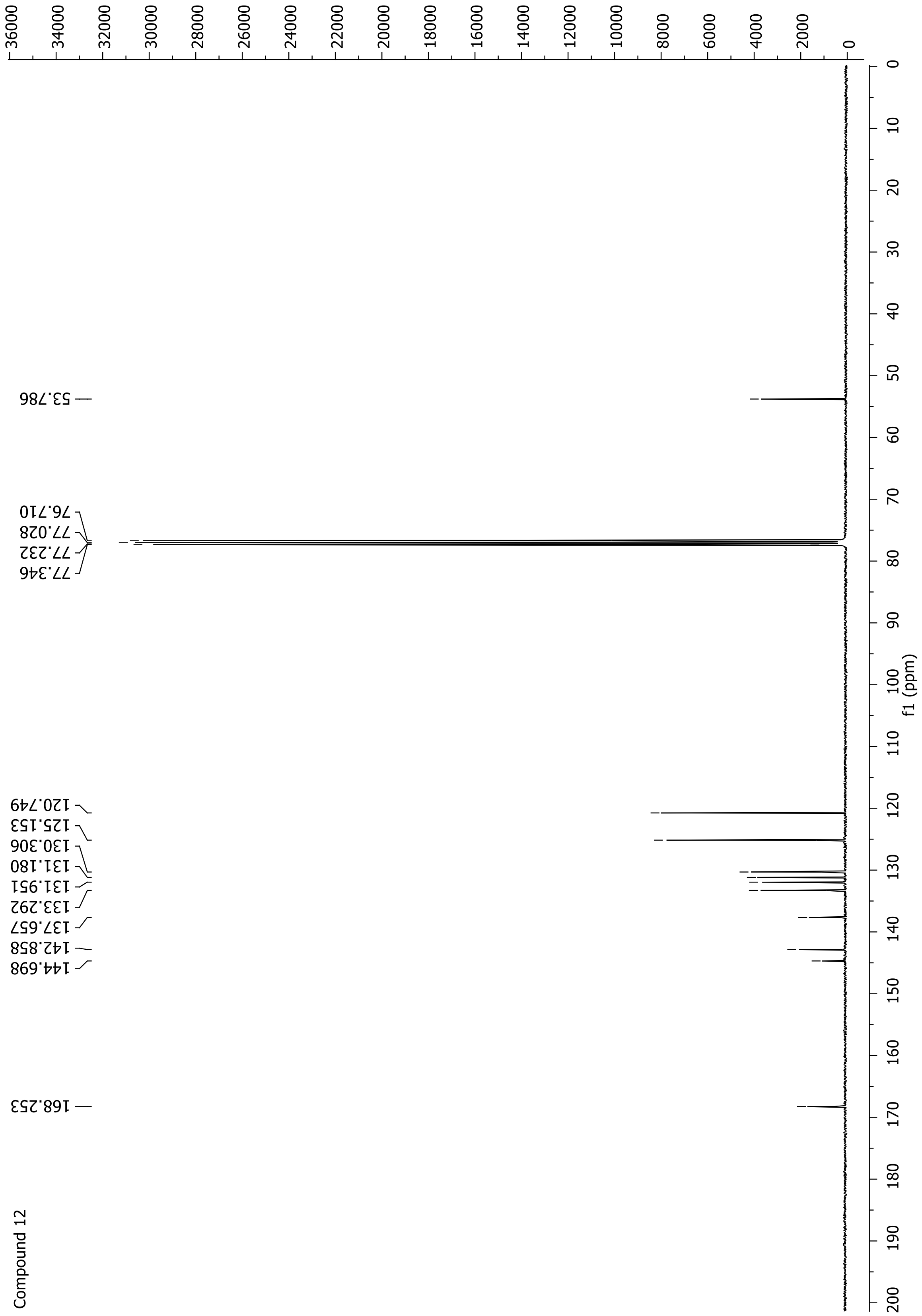

^{1}H-NMR of compound 16

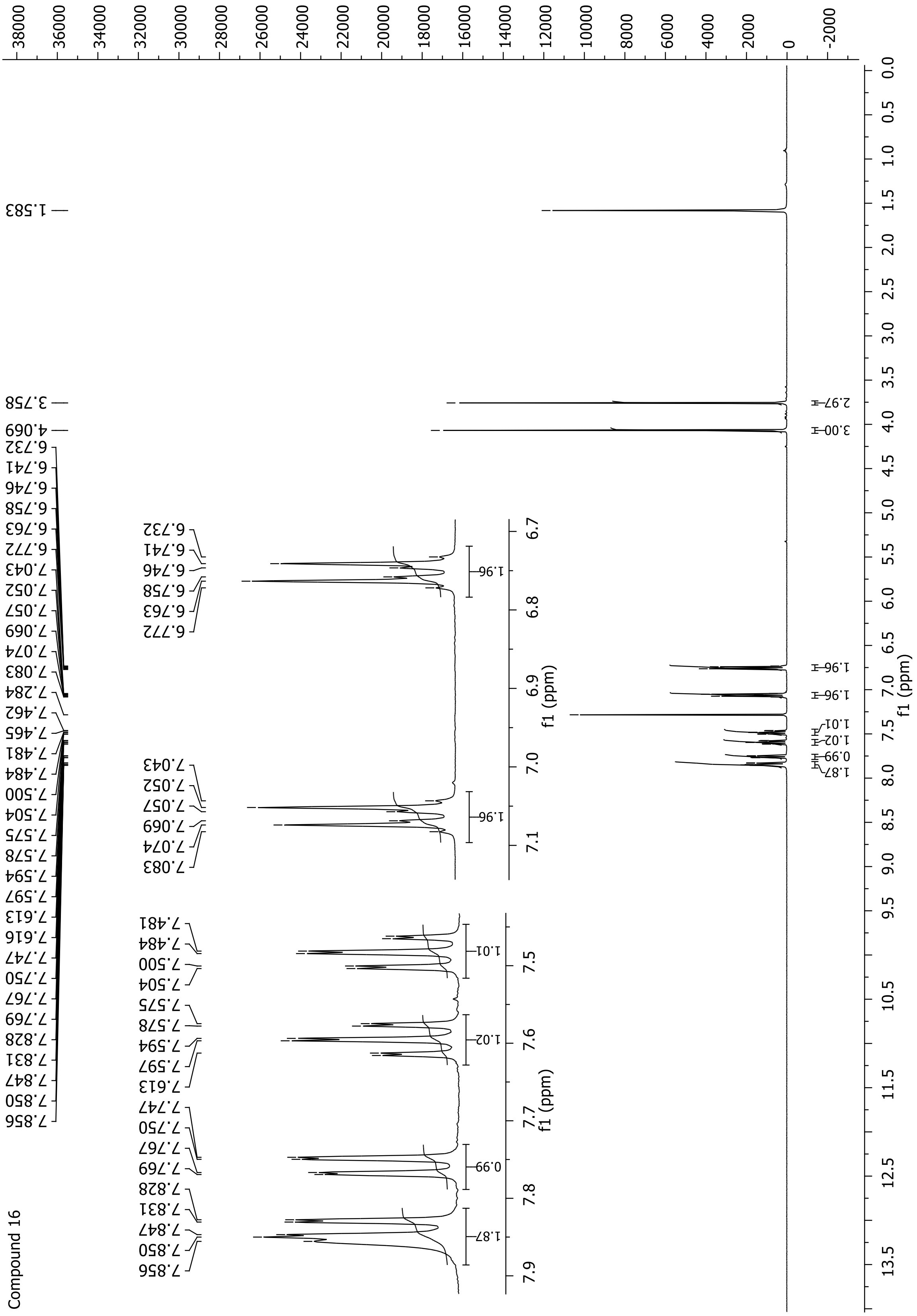

^{1}H-NMR of compound 18

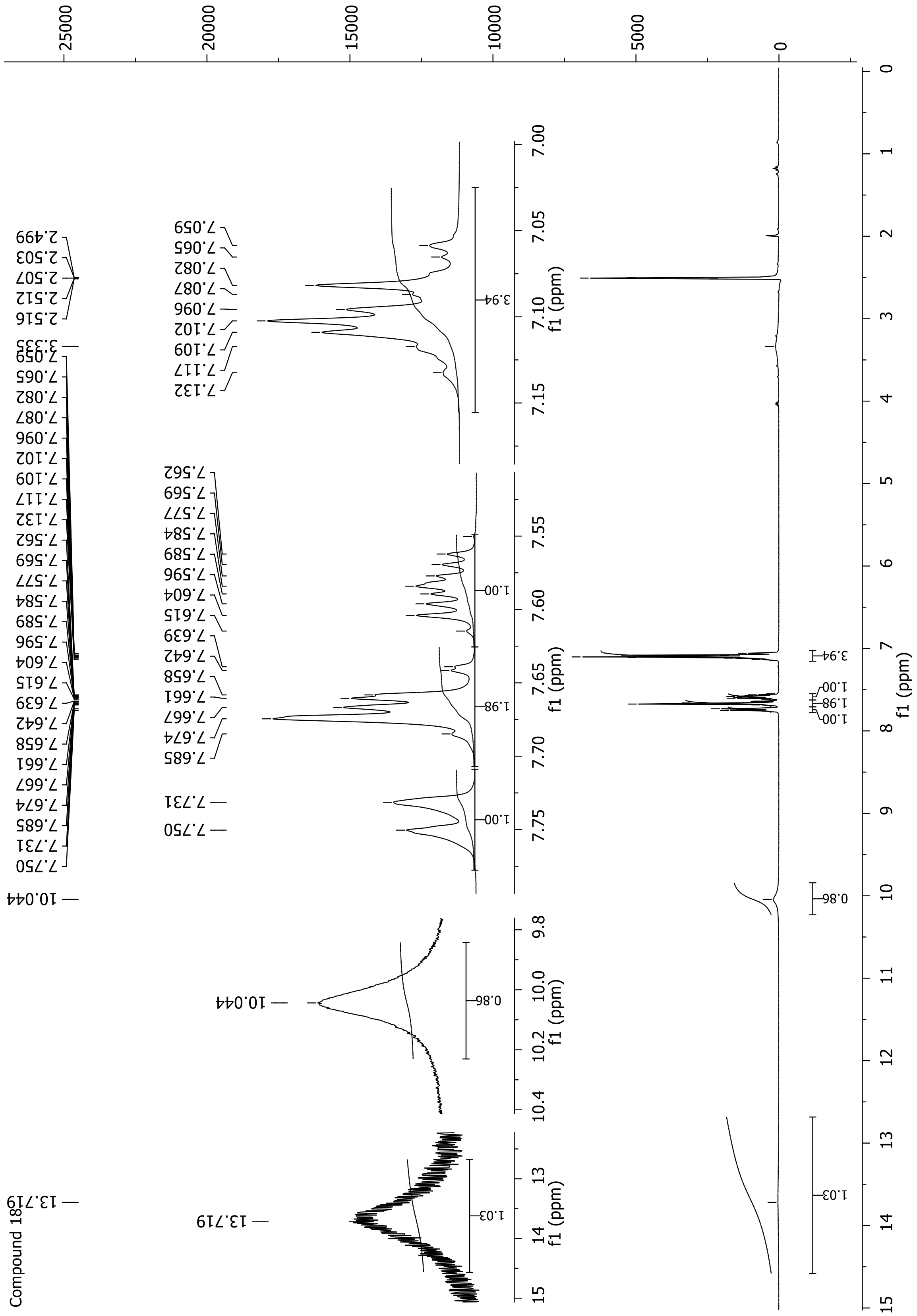

^{13}C-NMR of compound 18

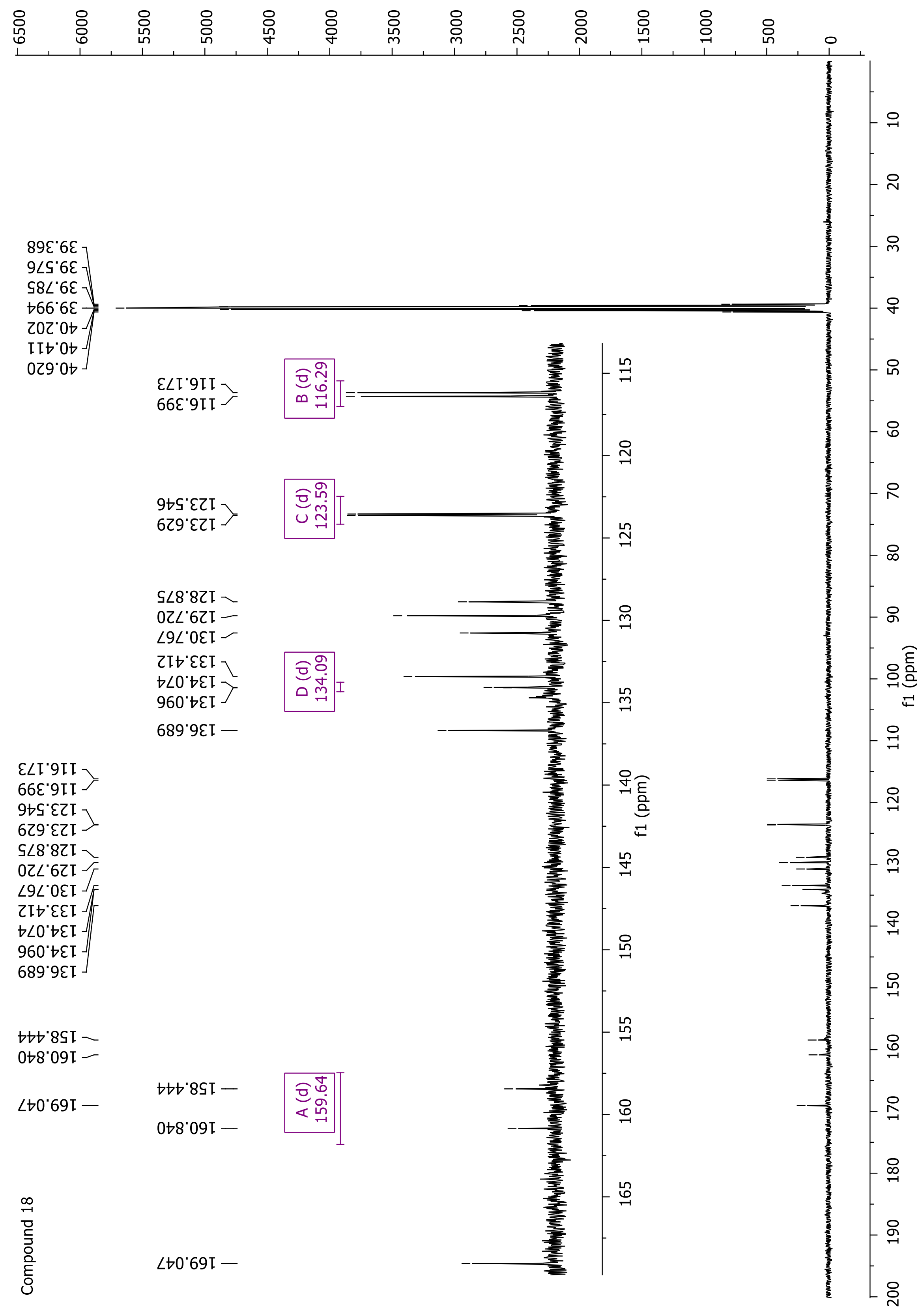

^{1}H-NMR of compound 20

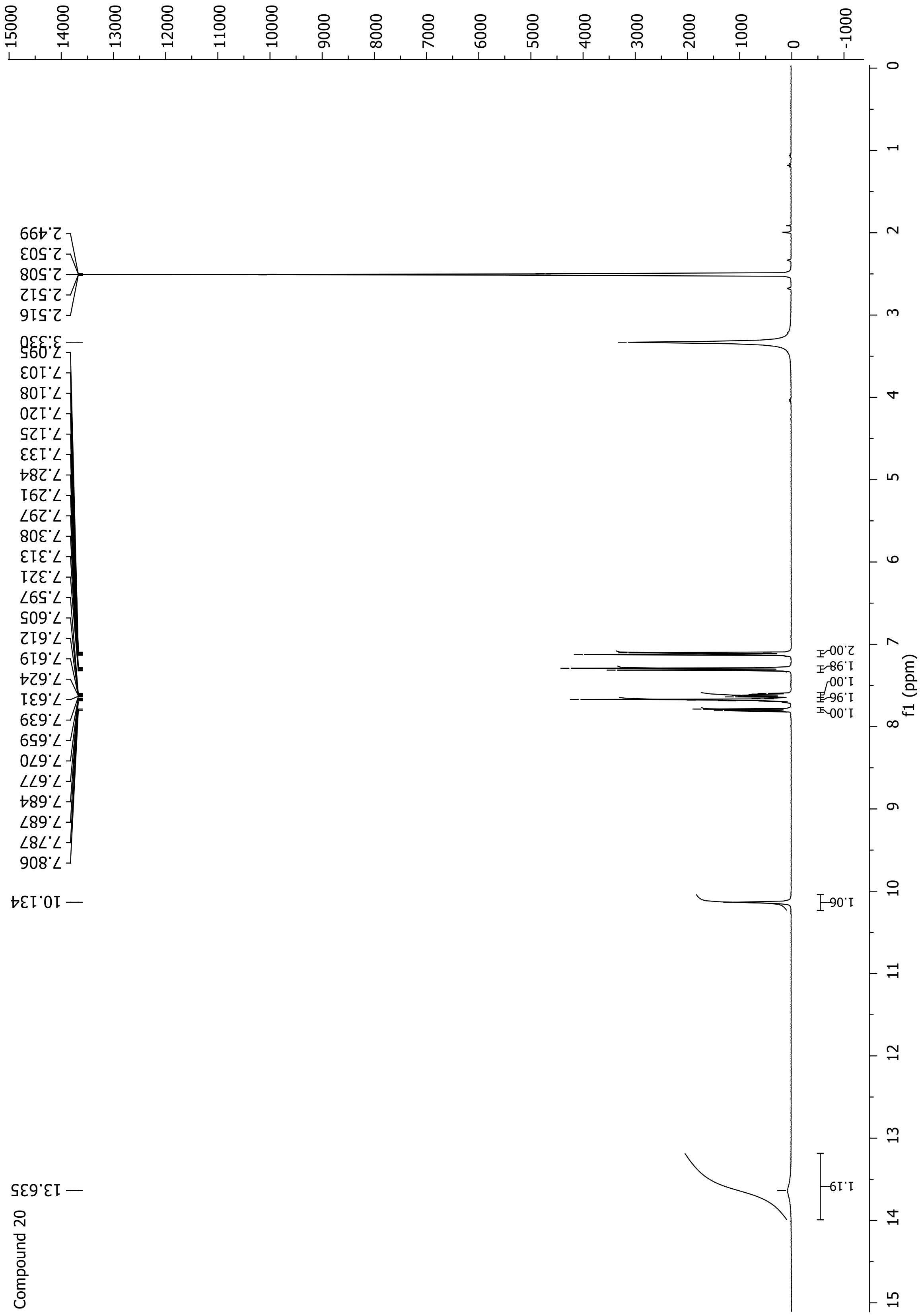

^{1}H-NMR of compound 23

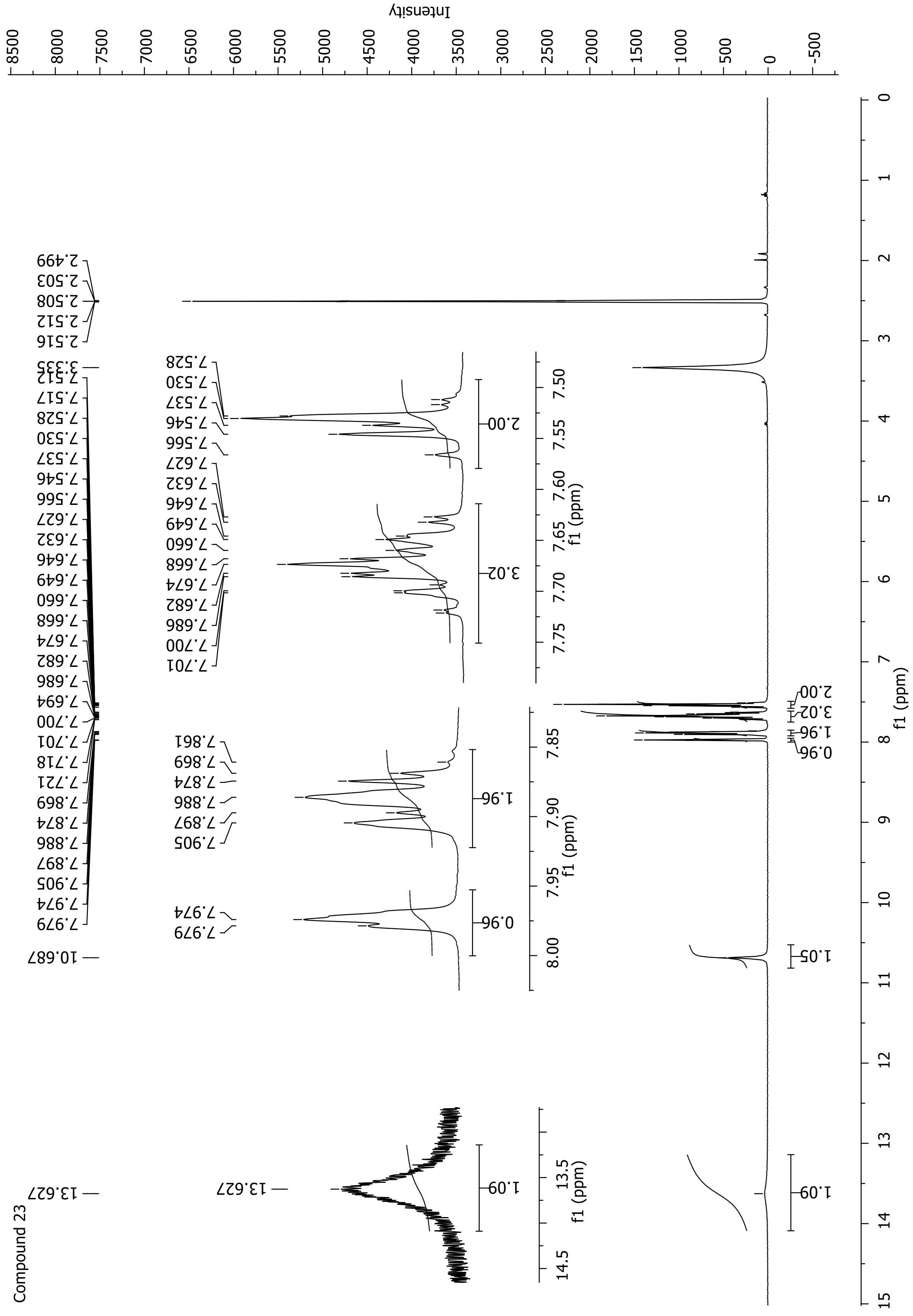

^{13}C-NMR of compound 23

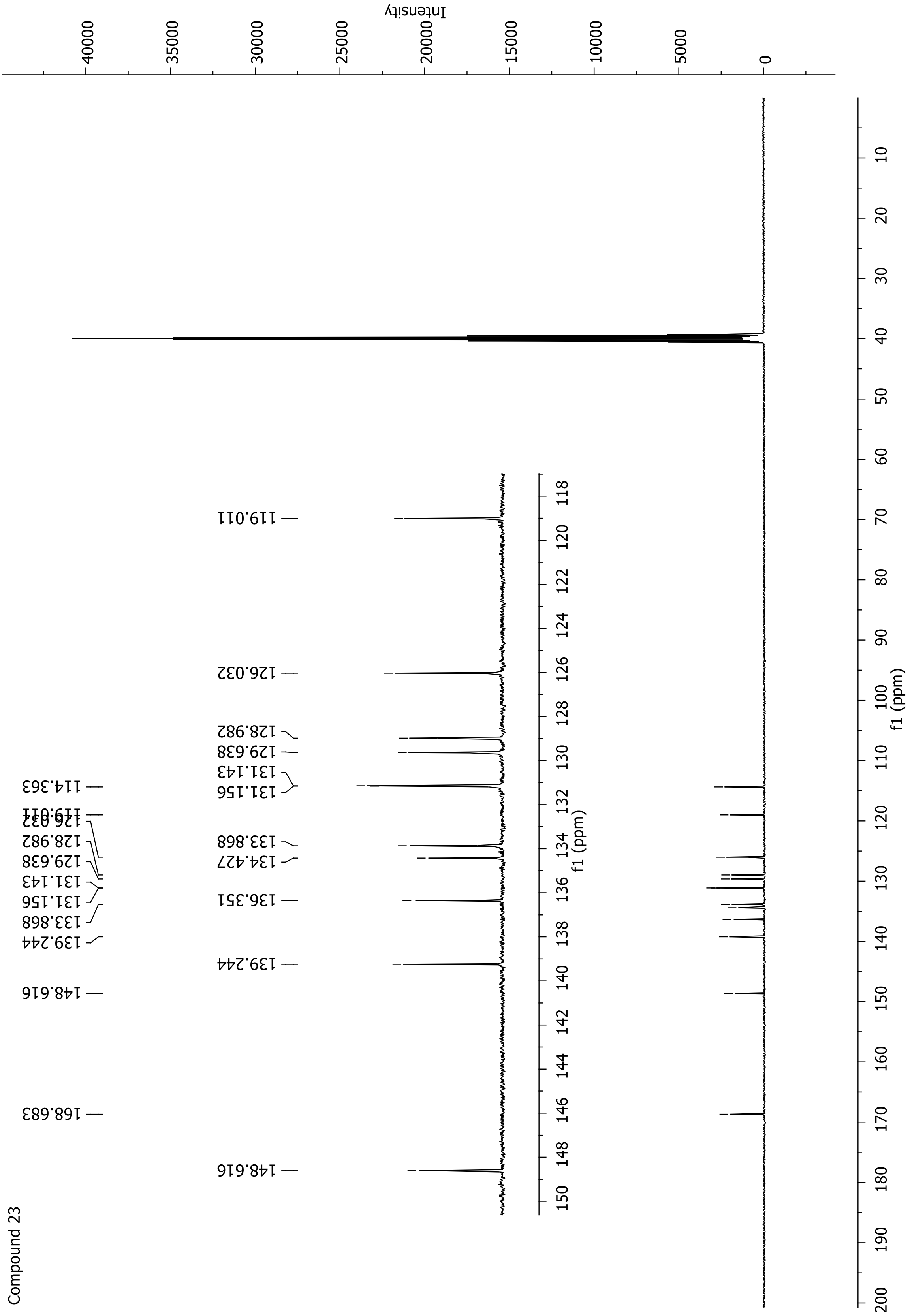

^{1}H-NMR of compound 24

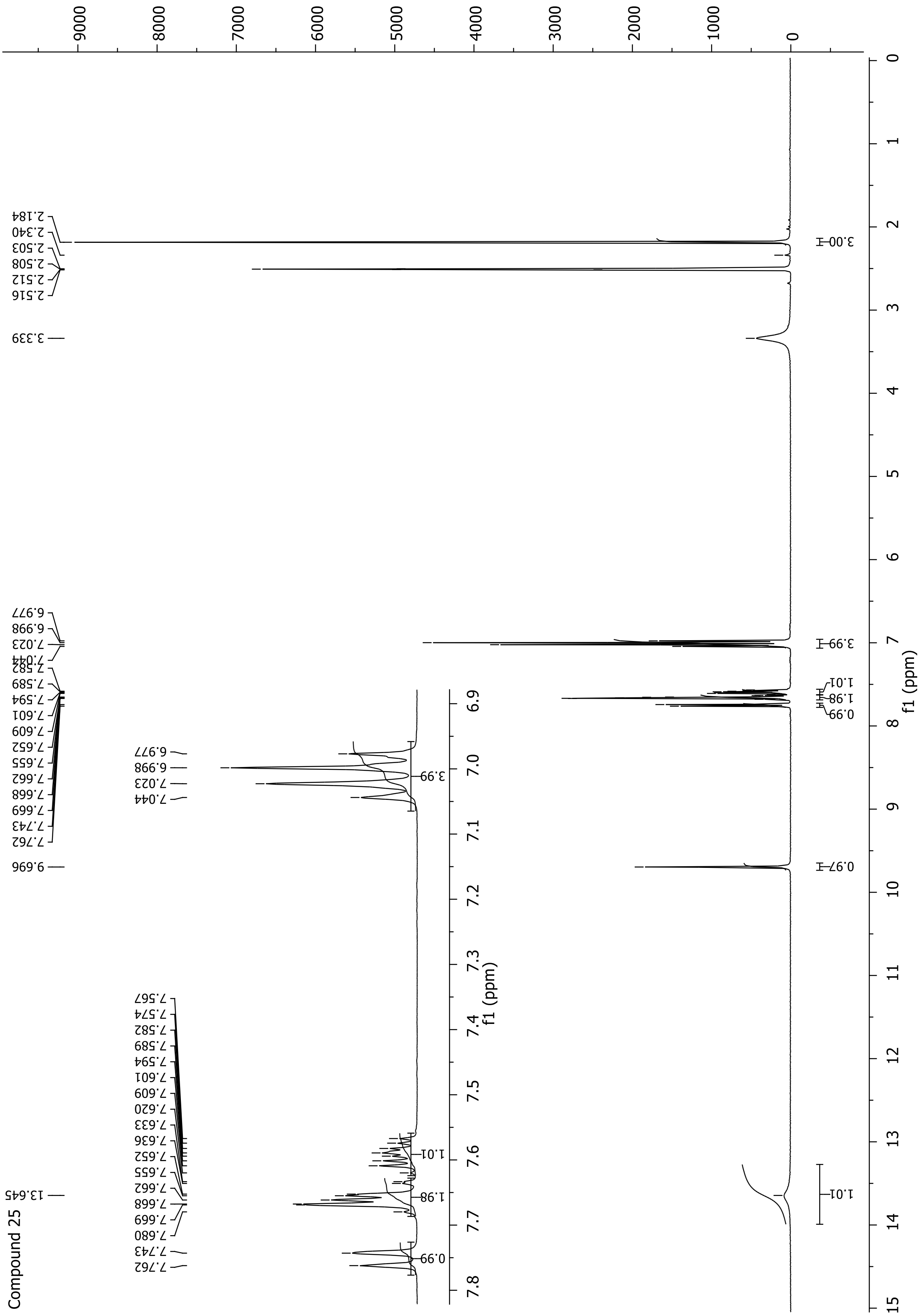

^{13}C-NMR of compound 24

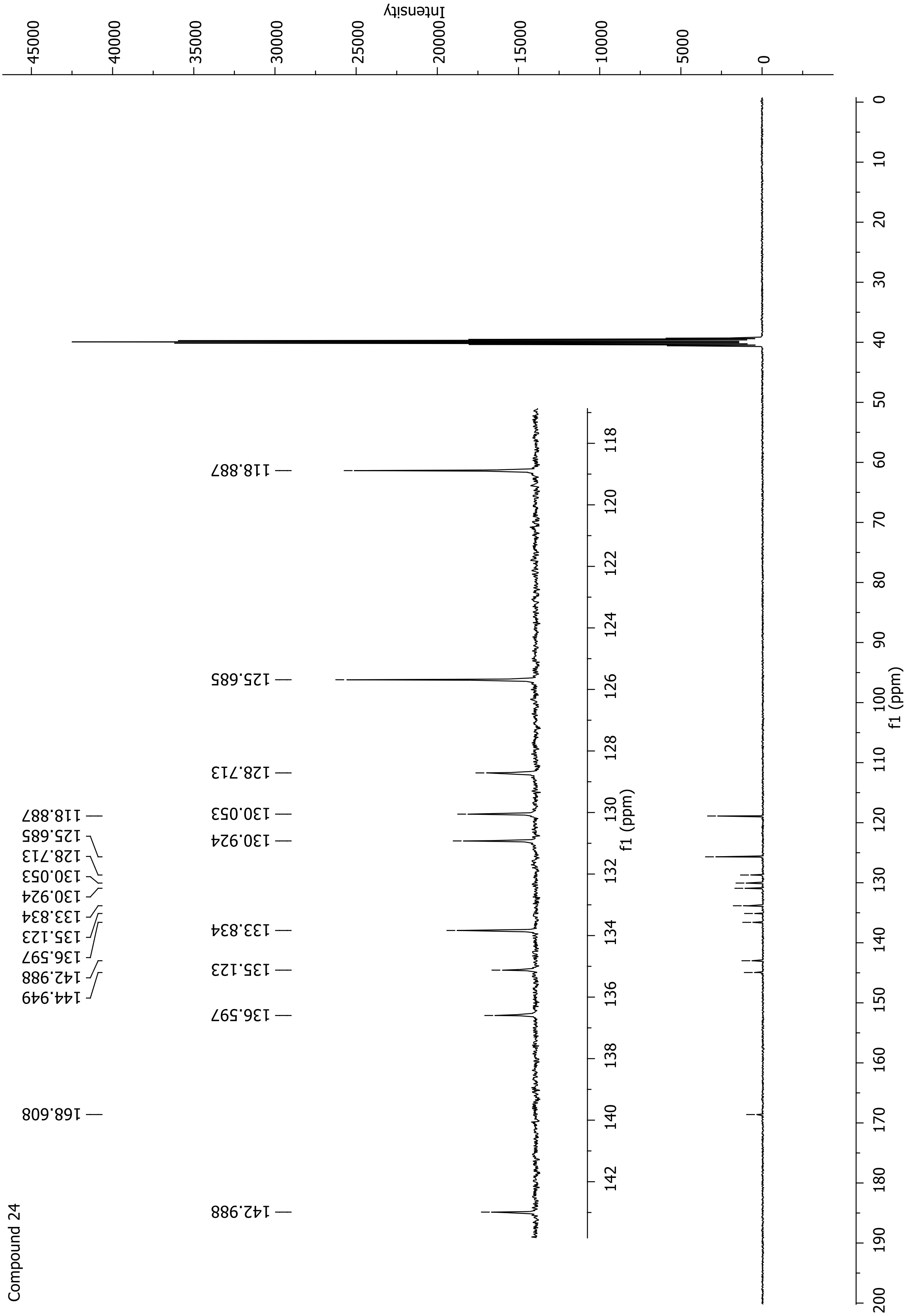

^{1}H-NMR of compound 27

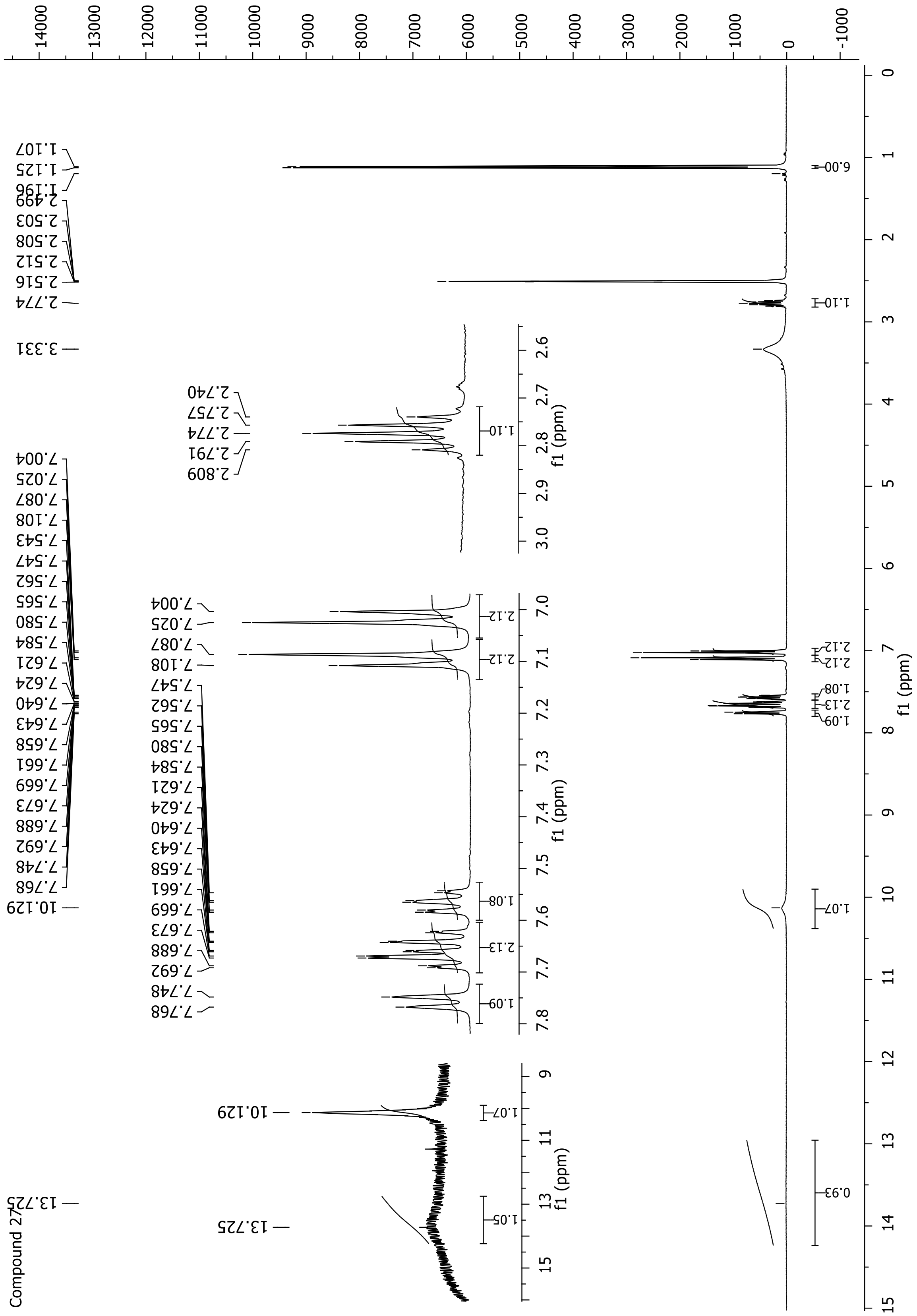

^{13}C-NMR of compound 27

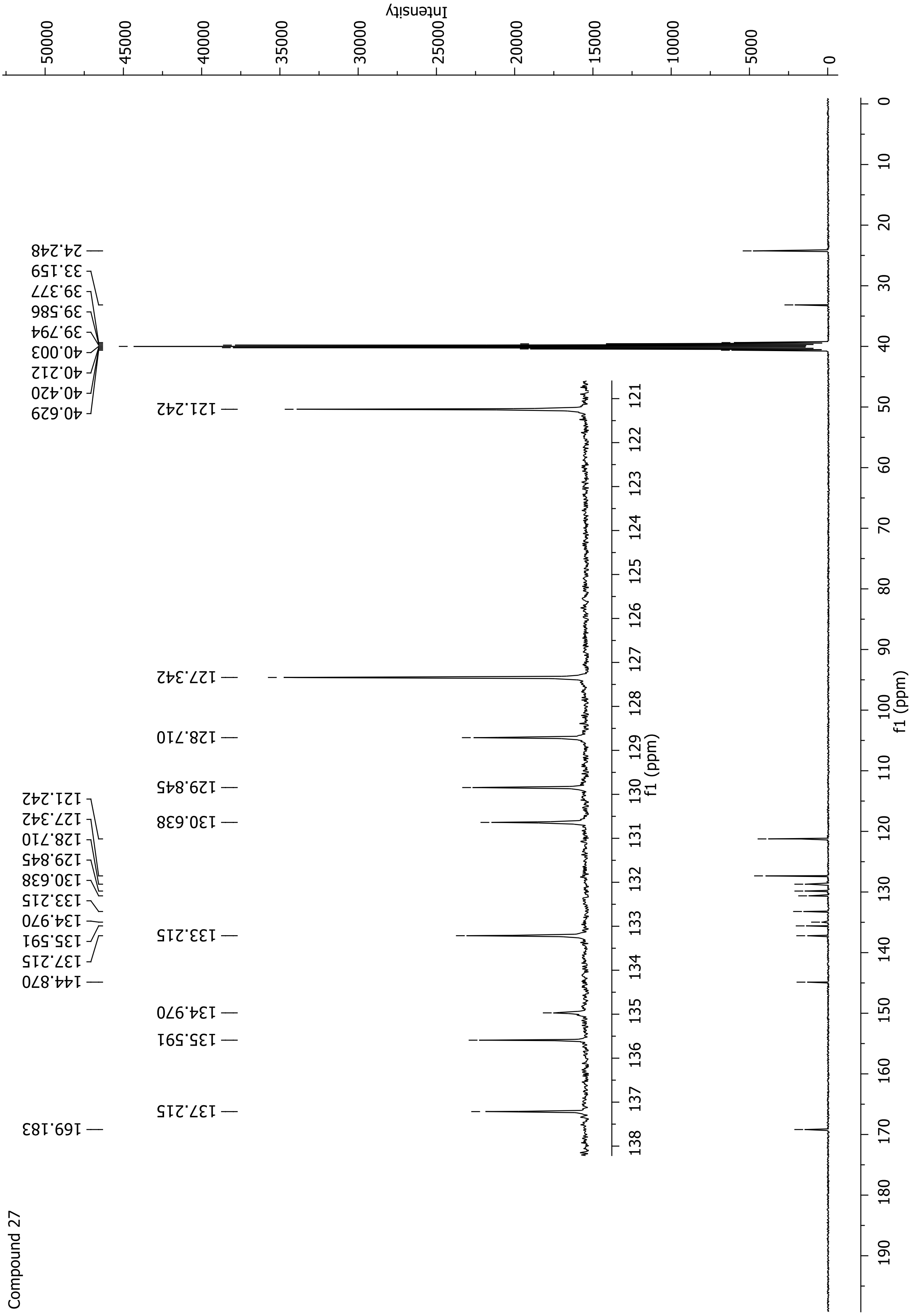

^{1}H-NMR of compound 30

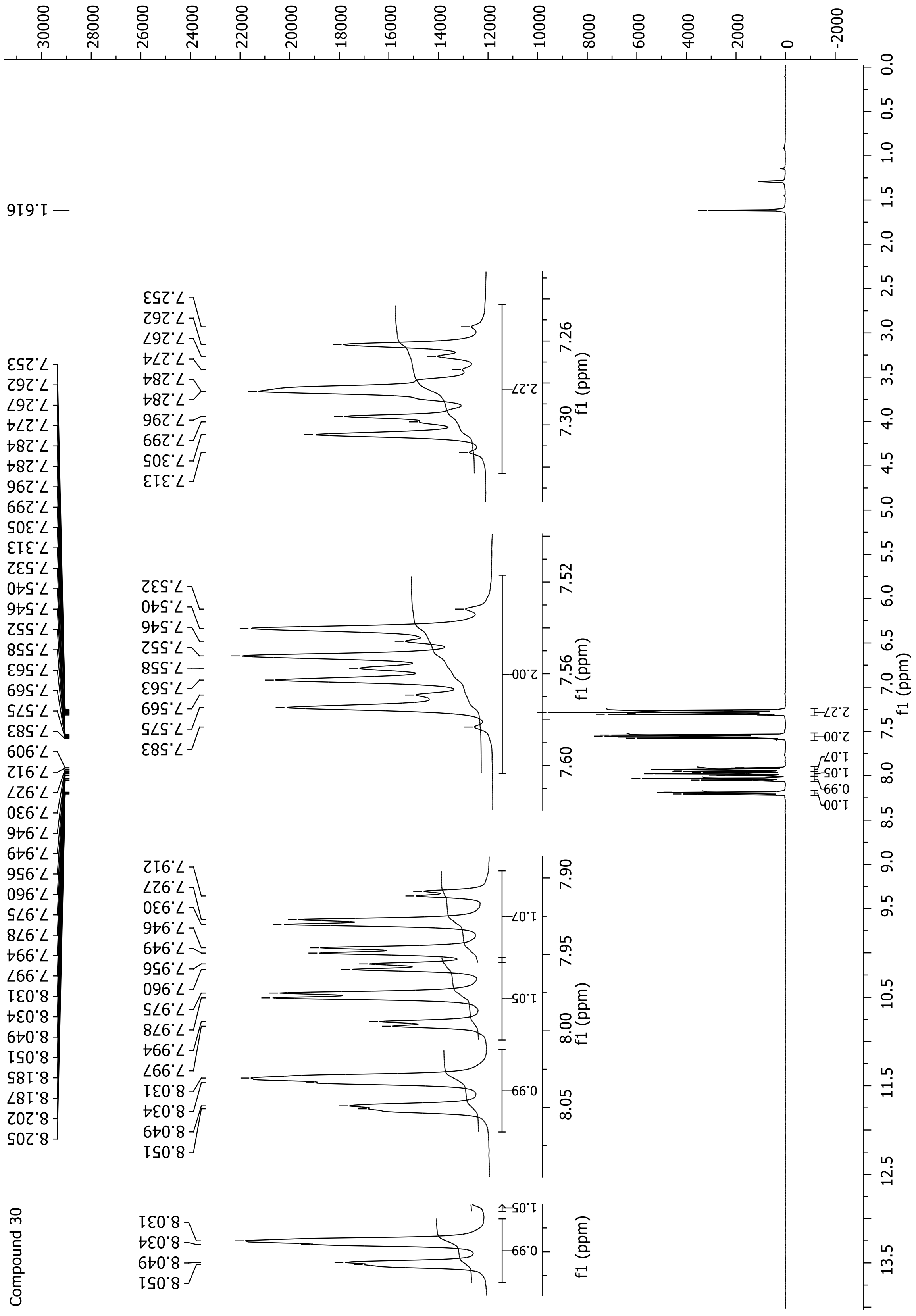

^{13}C-NMR of compound 30

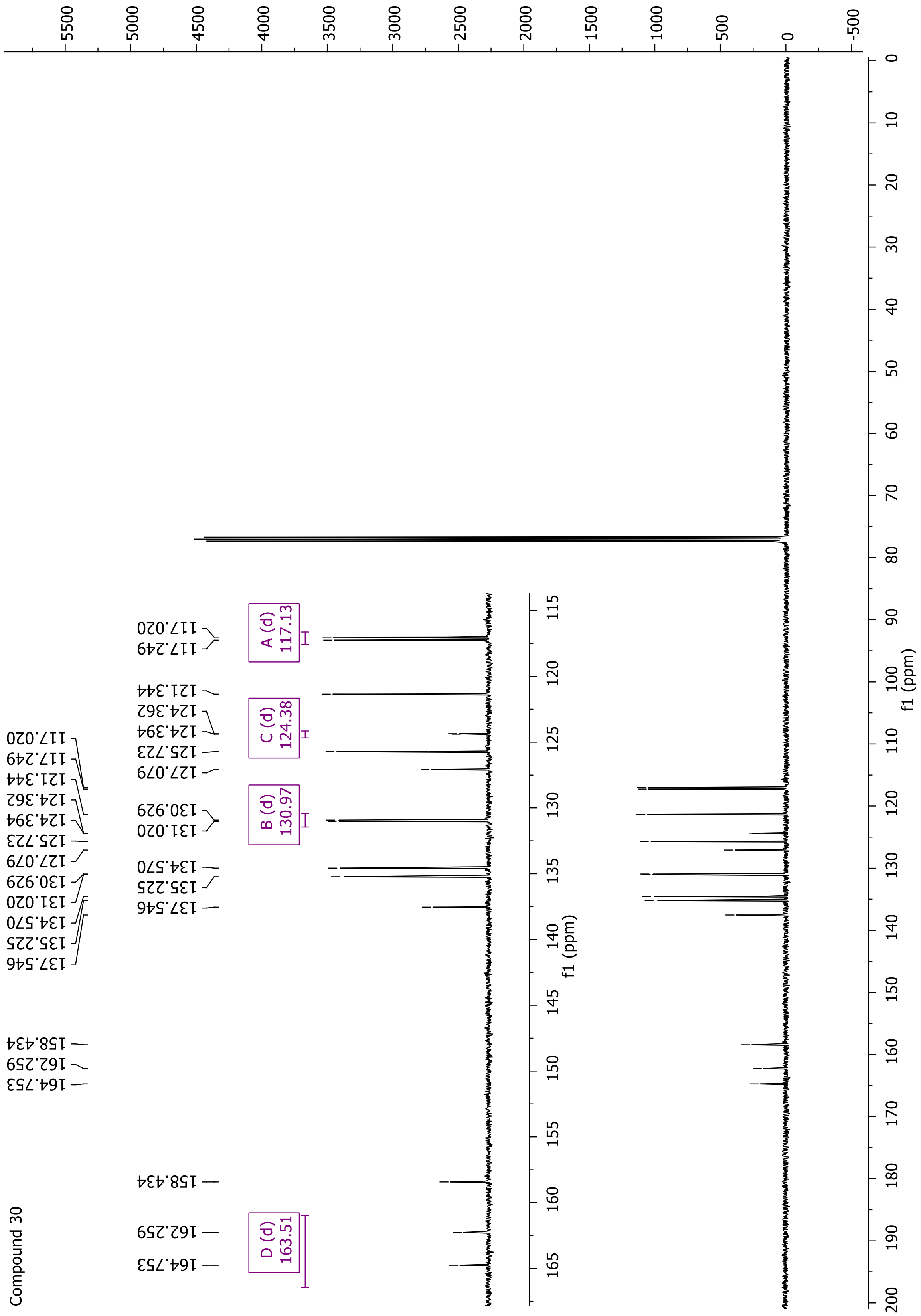

^{1}H-NMR of compound 31

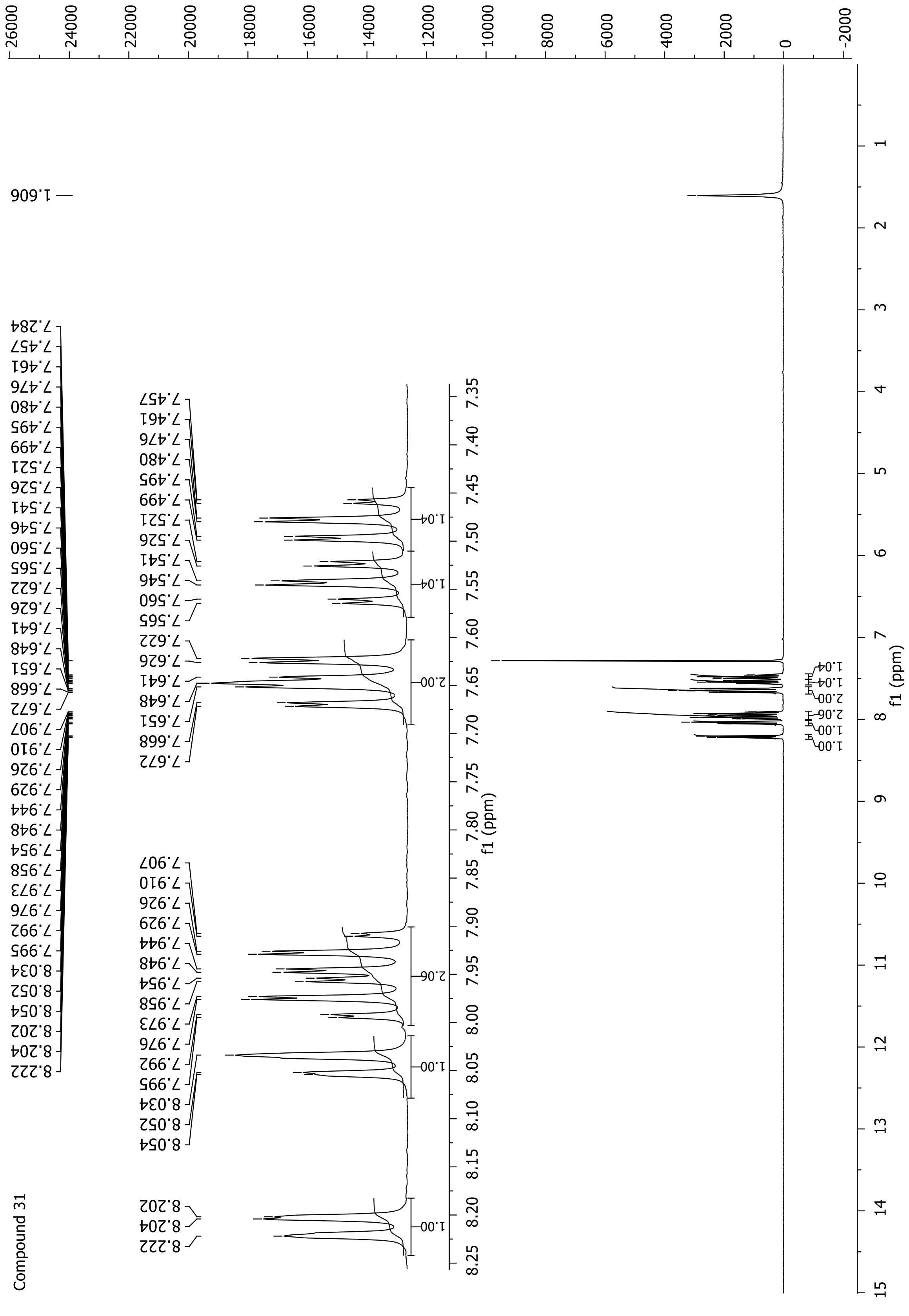

^{1}H-NMR of compound 33

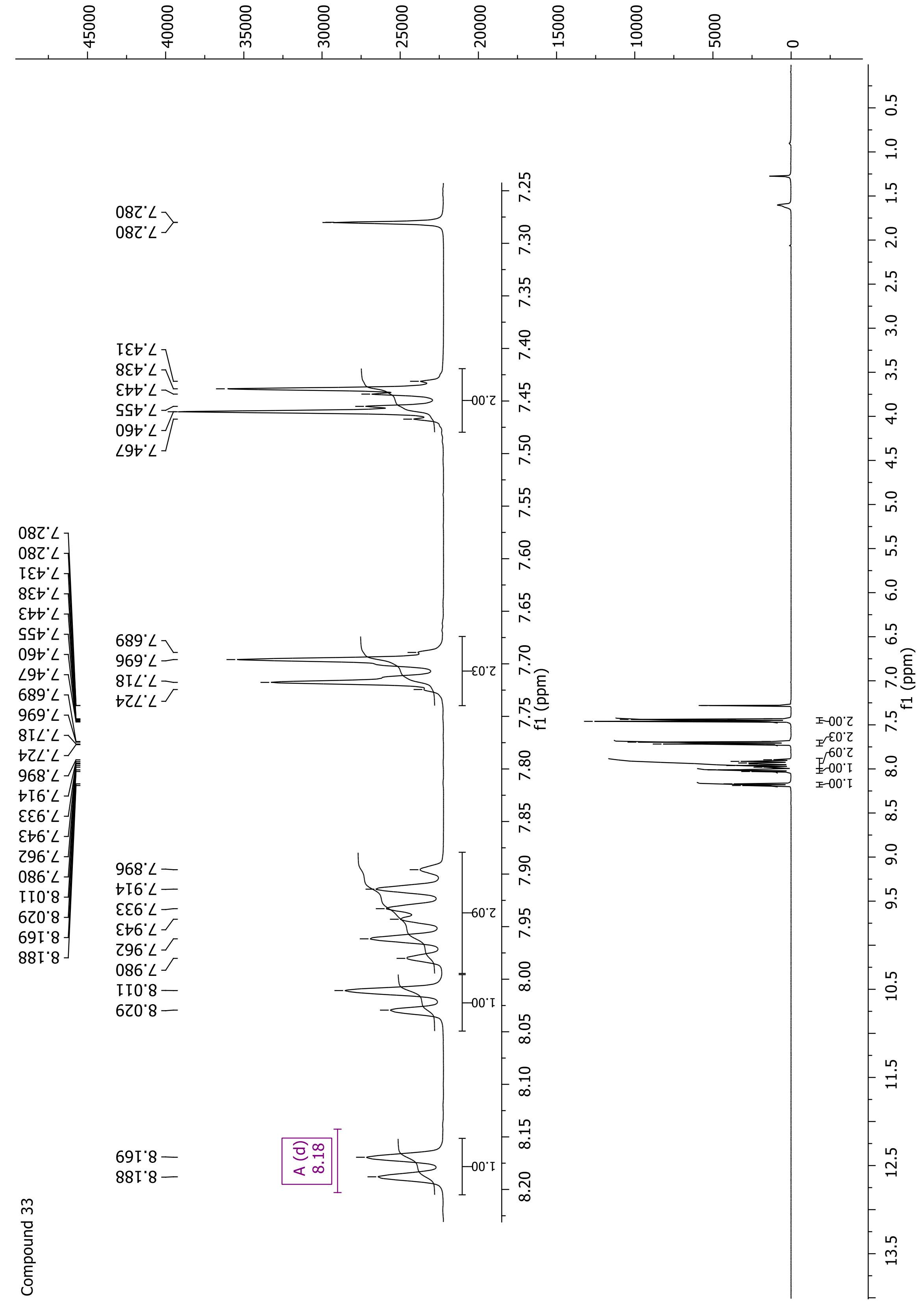

^{13}C-NMR of compound 33

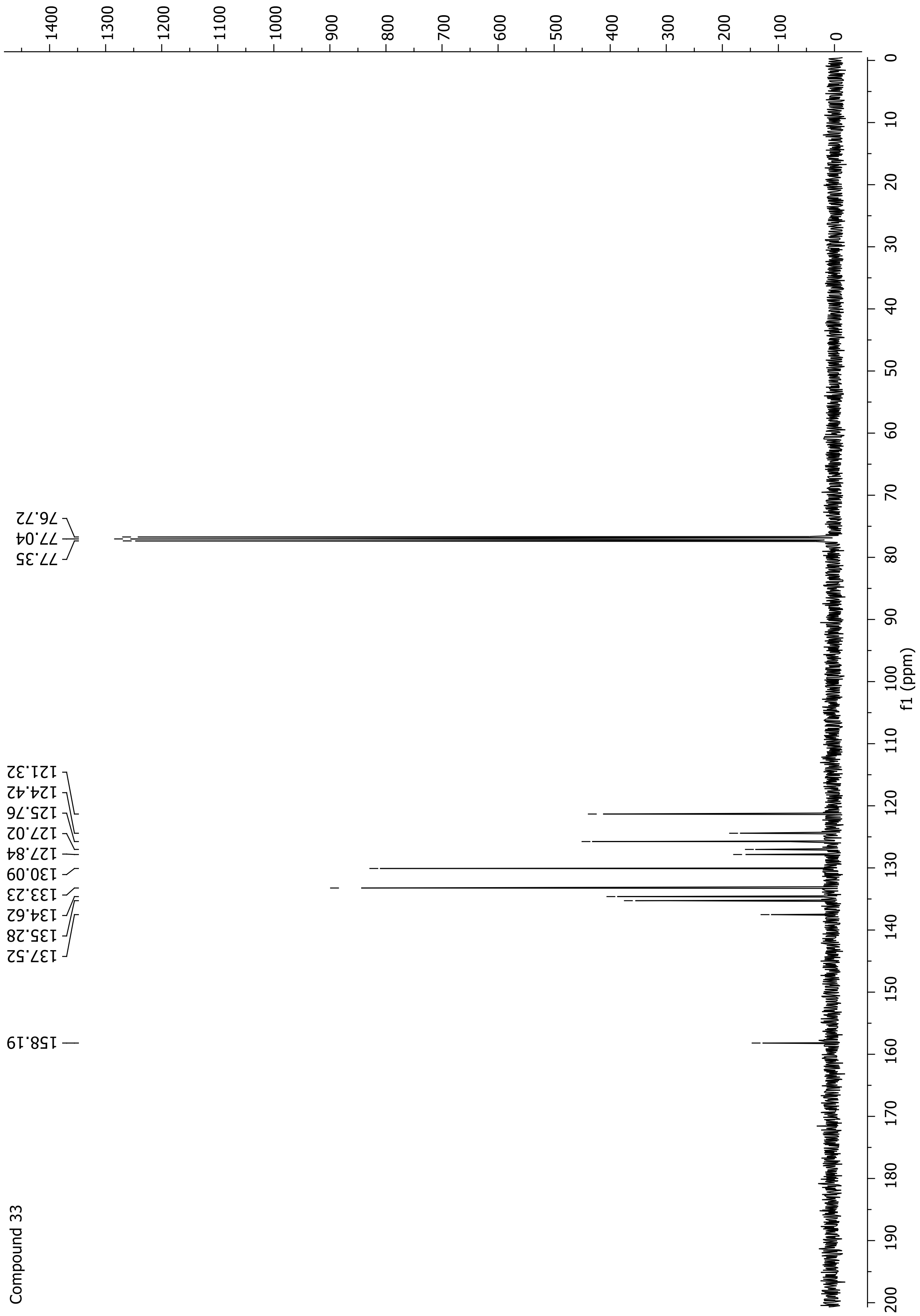

^{1}H-NMR of compound 34

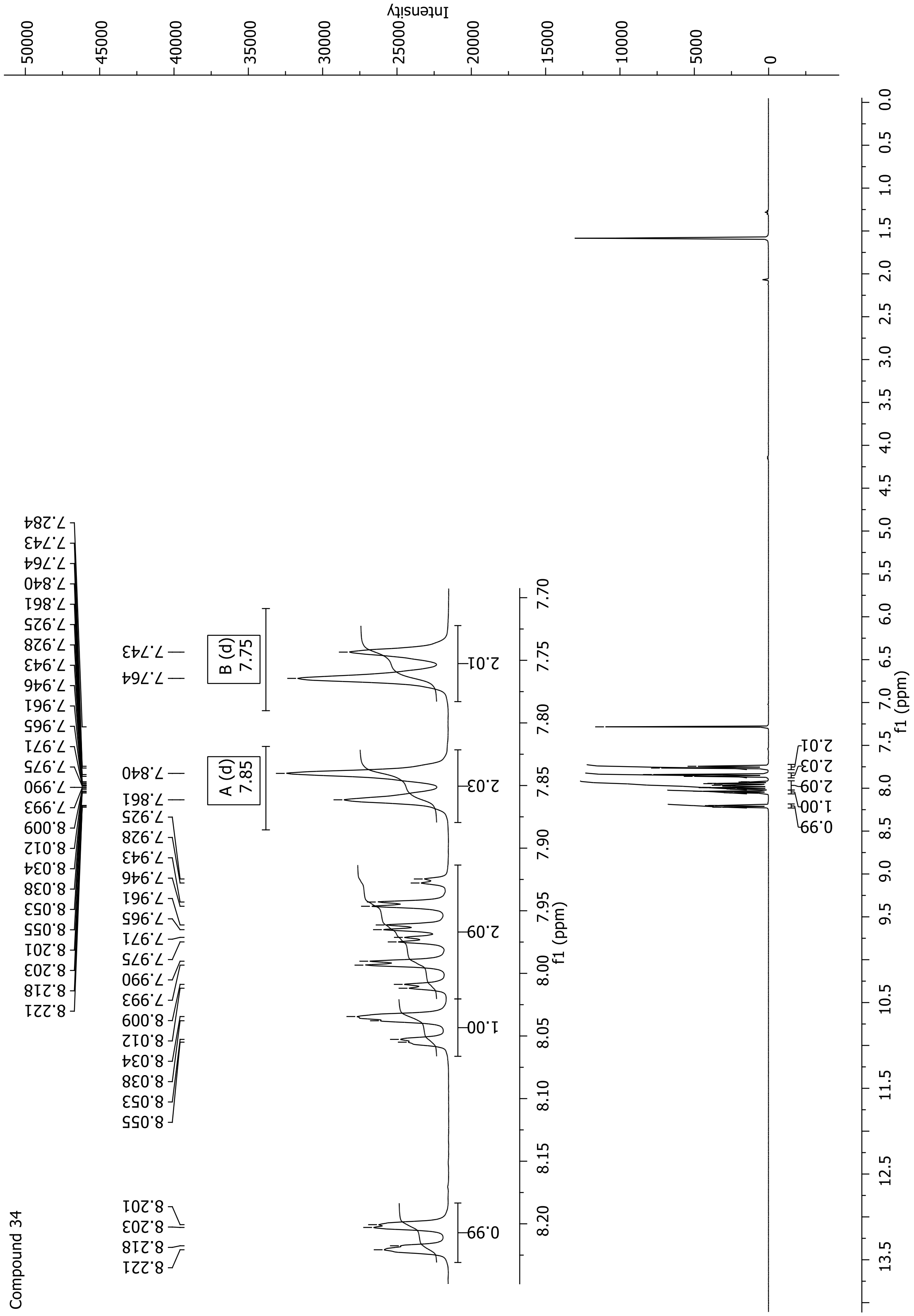

^{13}C-NMR of compound 34

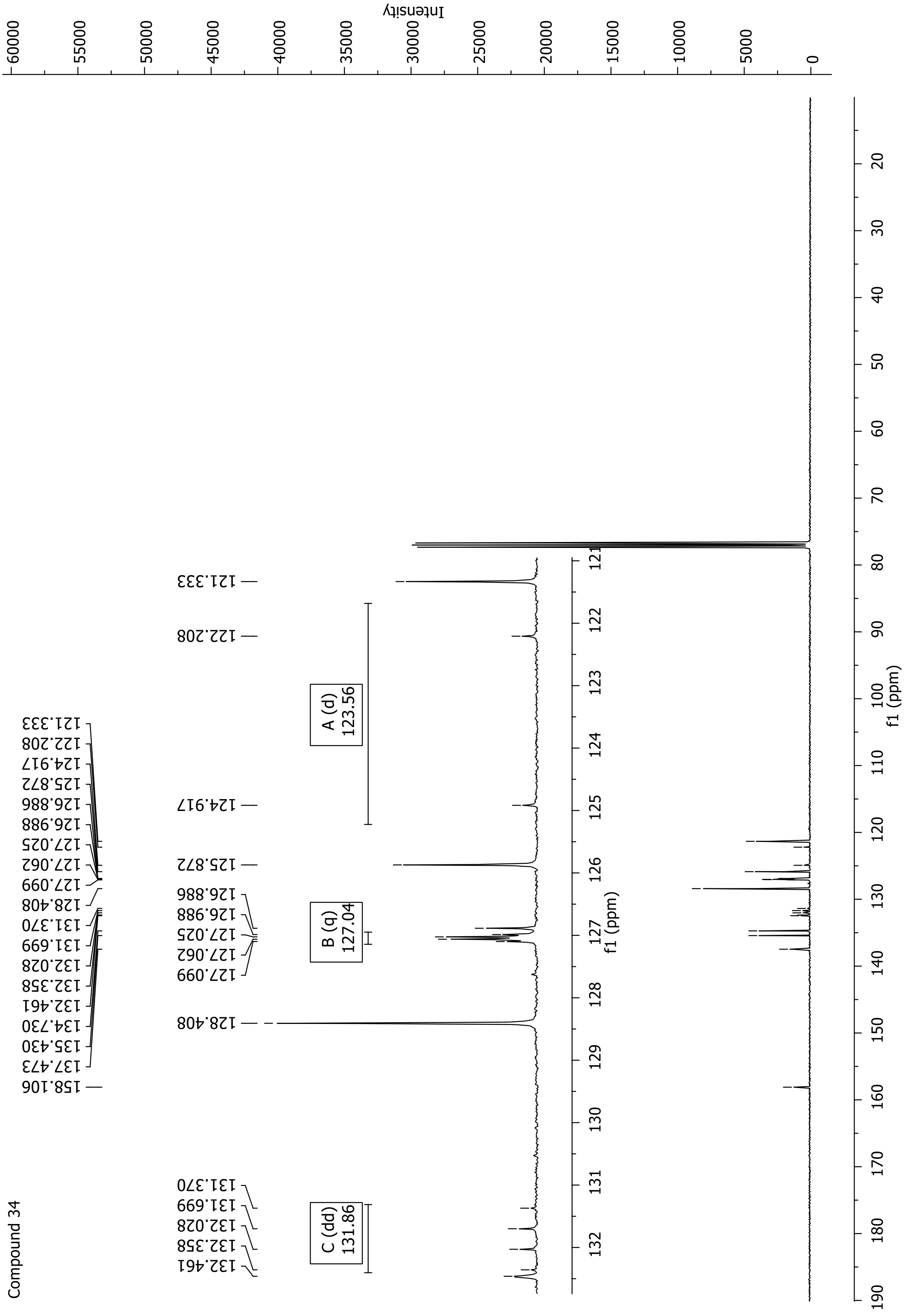

^{13}C-NMR of compound 36

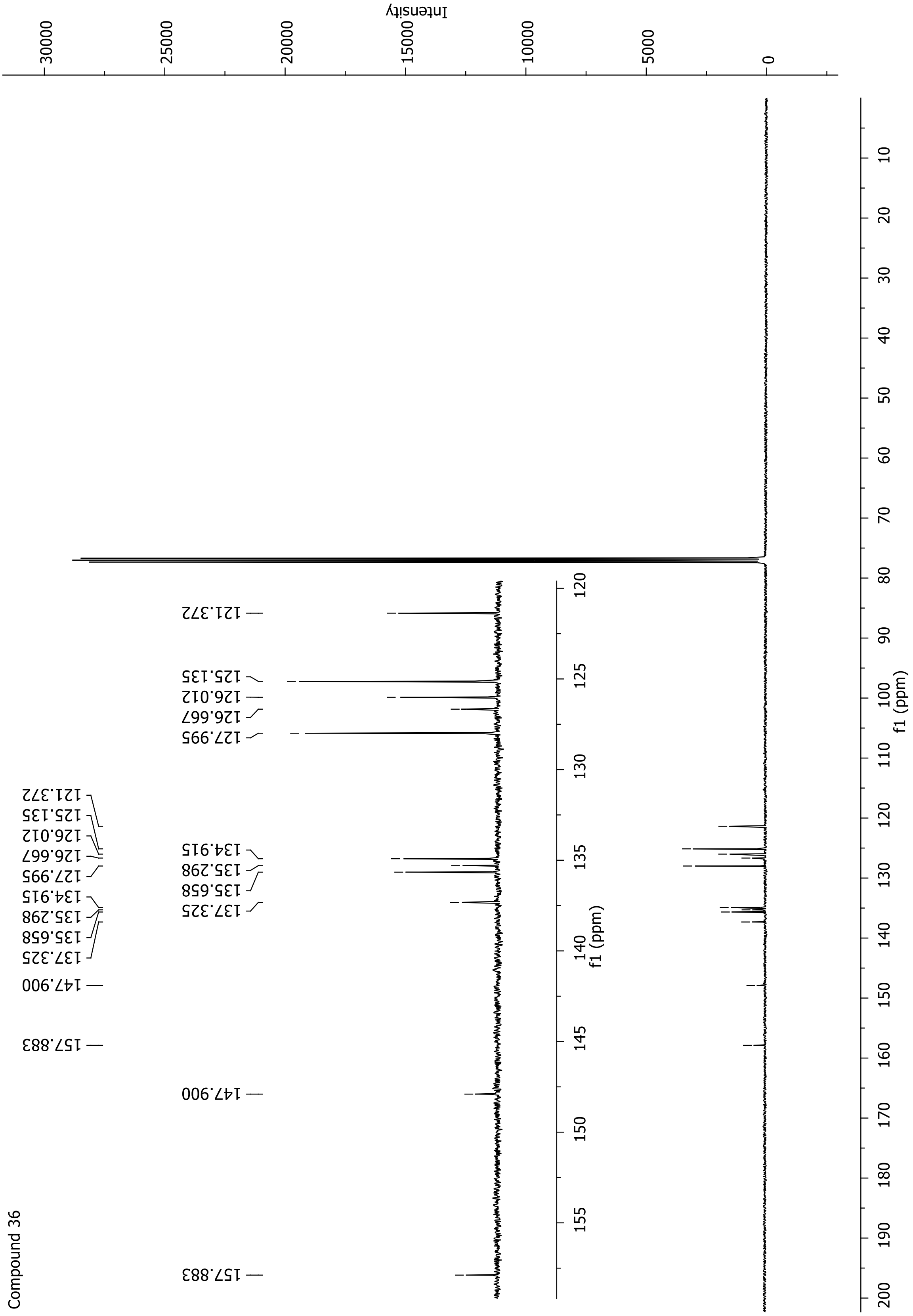

^{1}H-NMR of compound 37

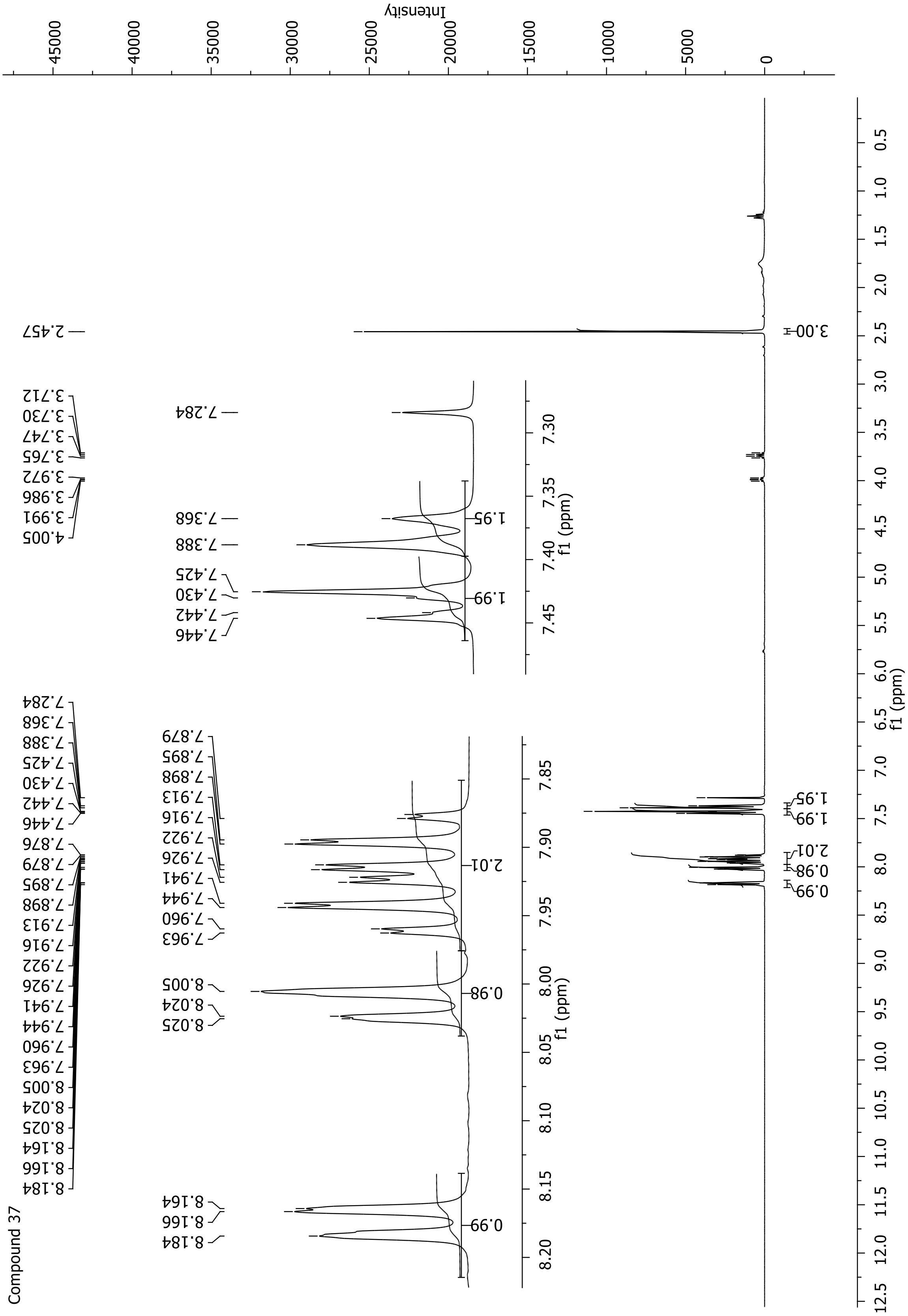

^{13}C-NMR of compound 37

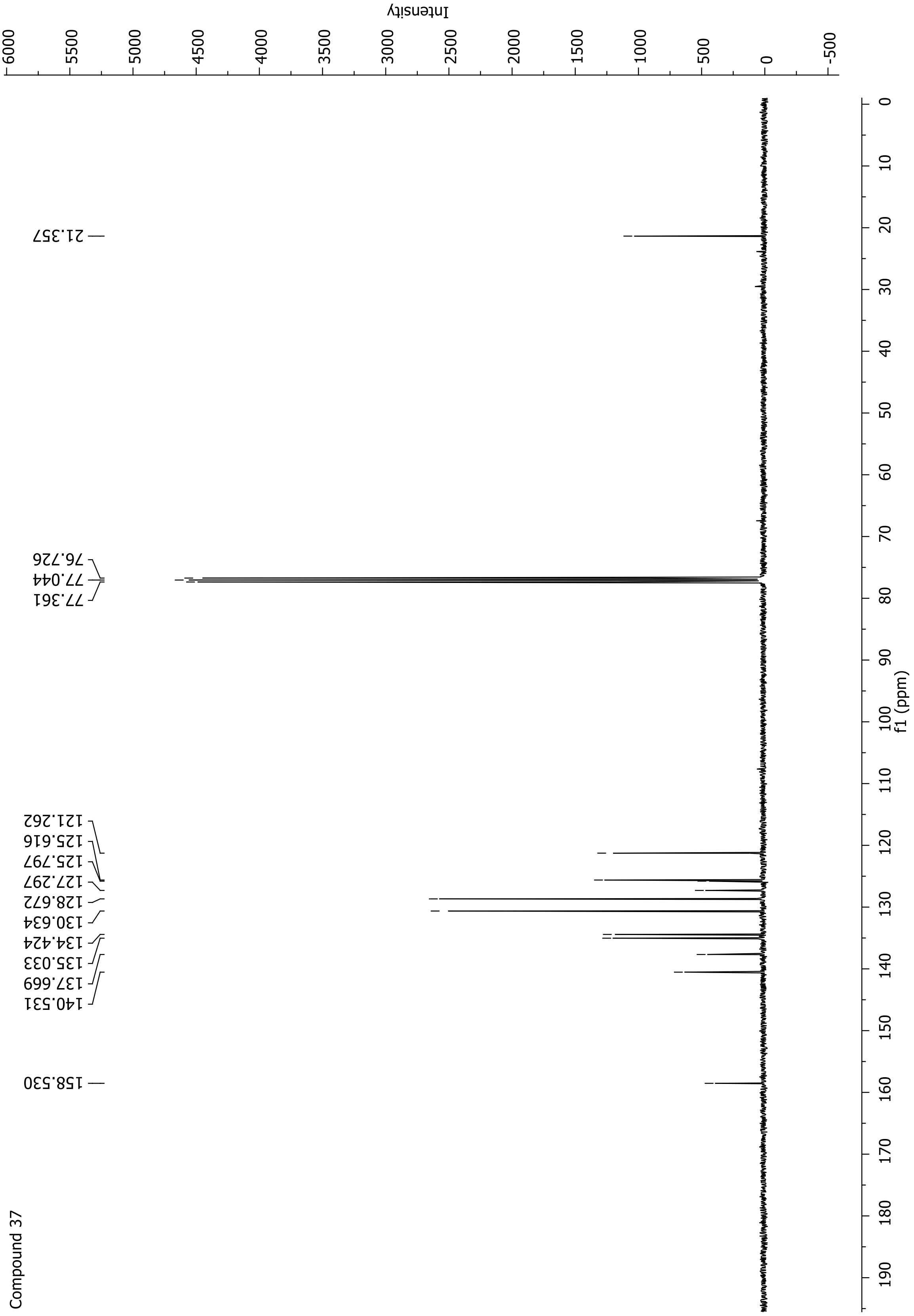

^{1}H-NMR of compound 38

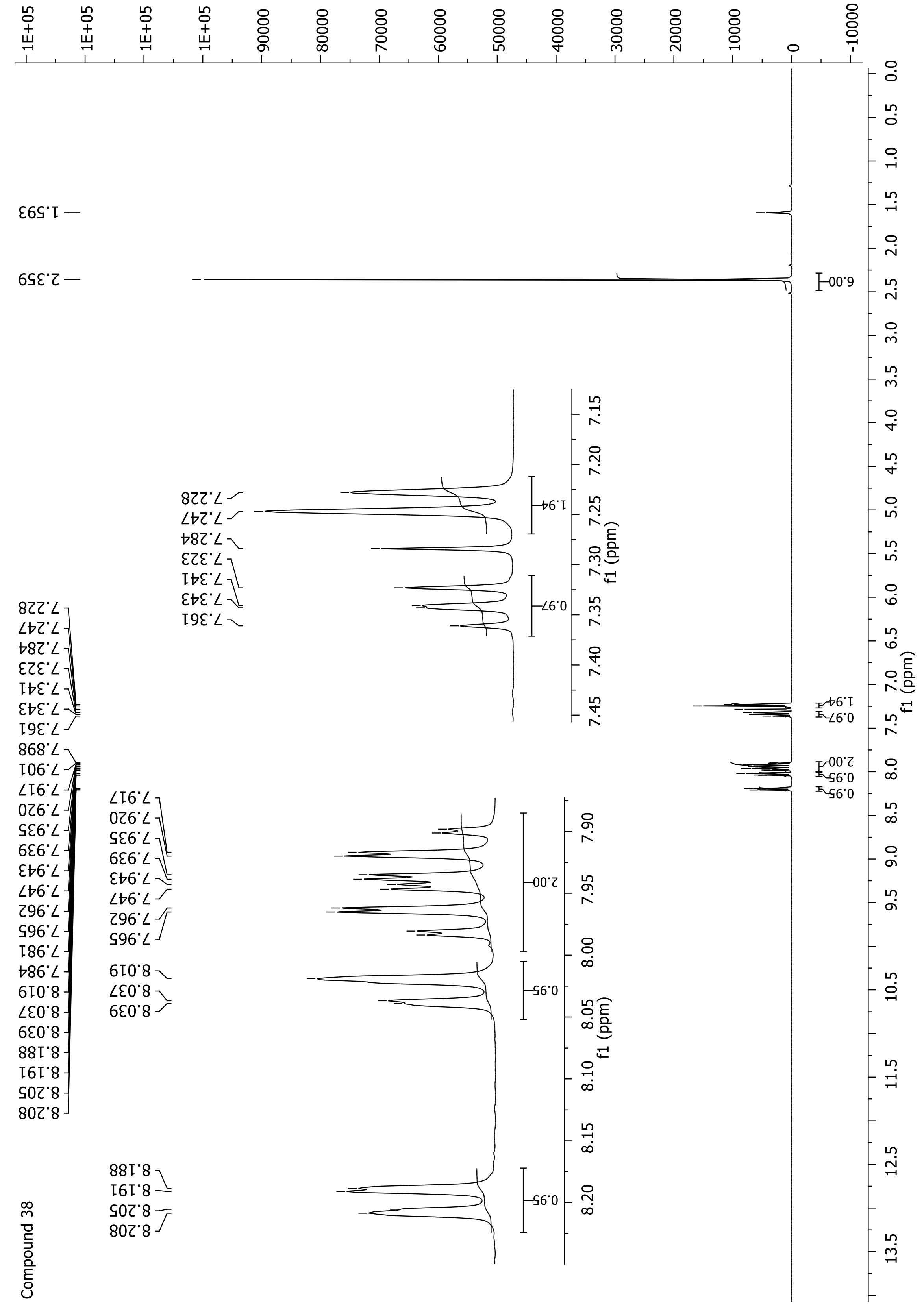

^{13}C-NMR of compound 38

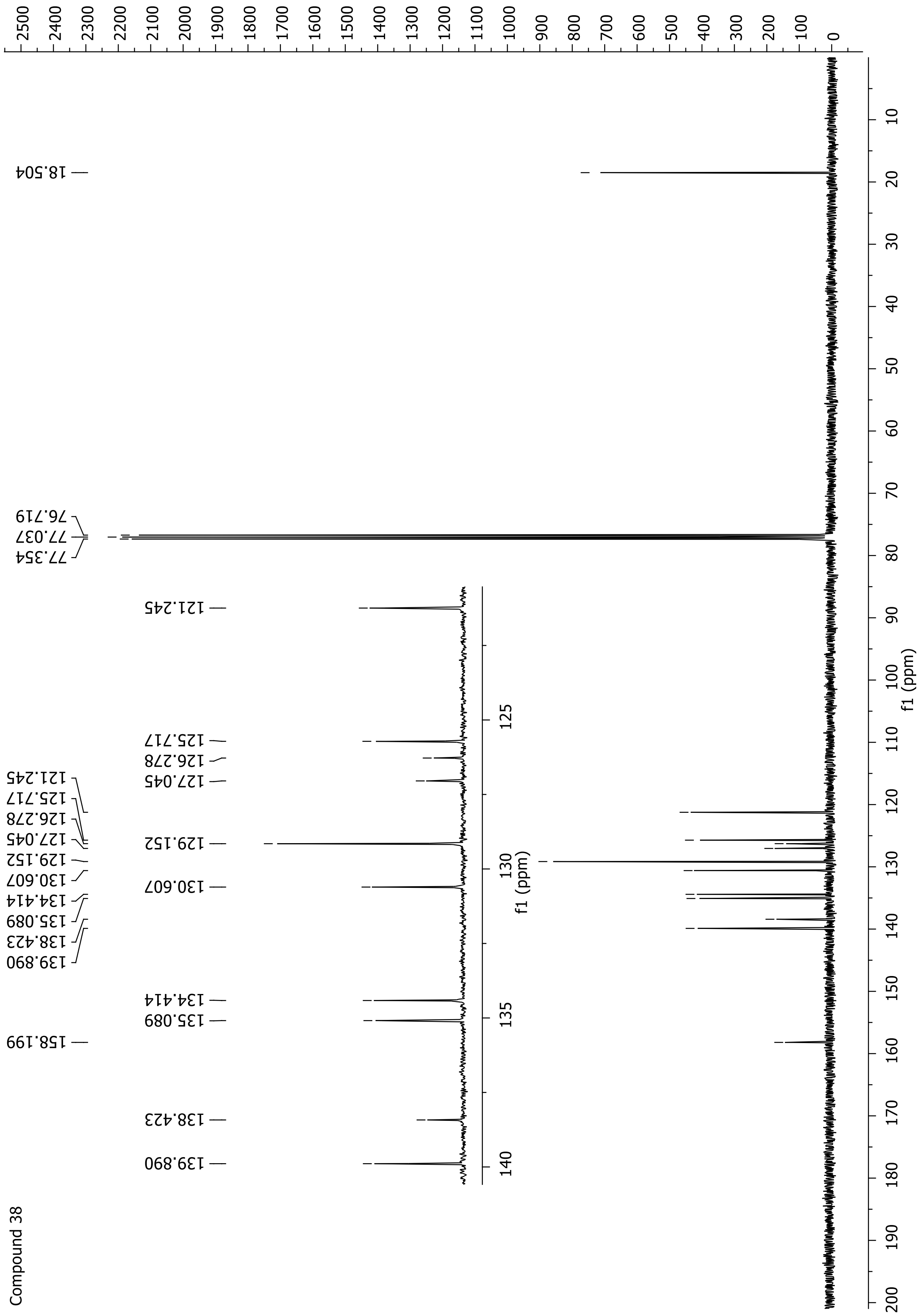

^{1}H-NMR of compound 39

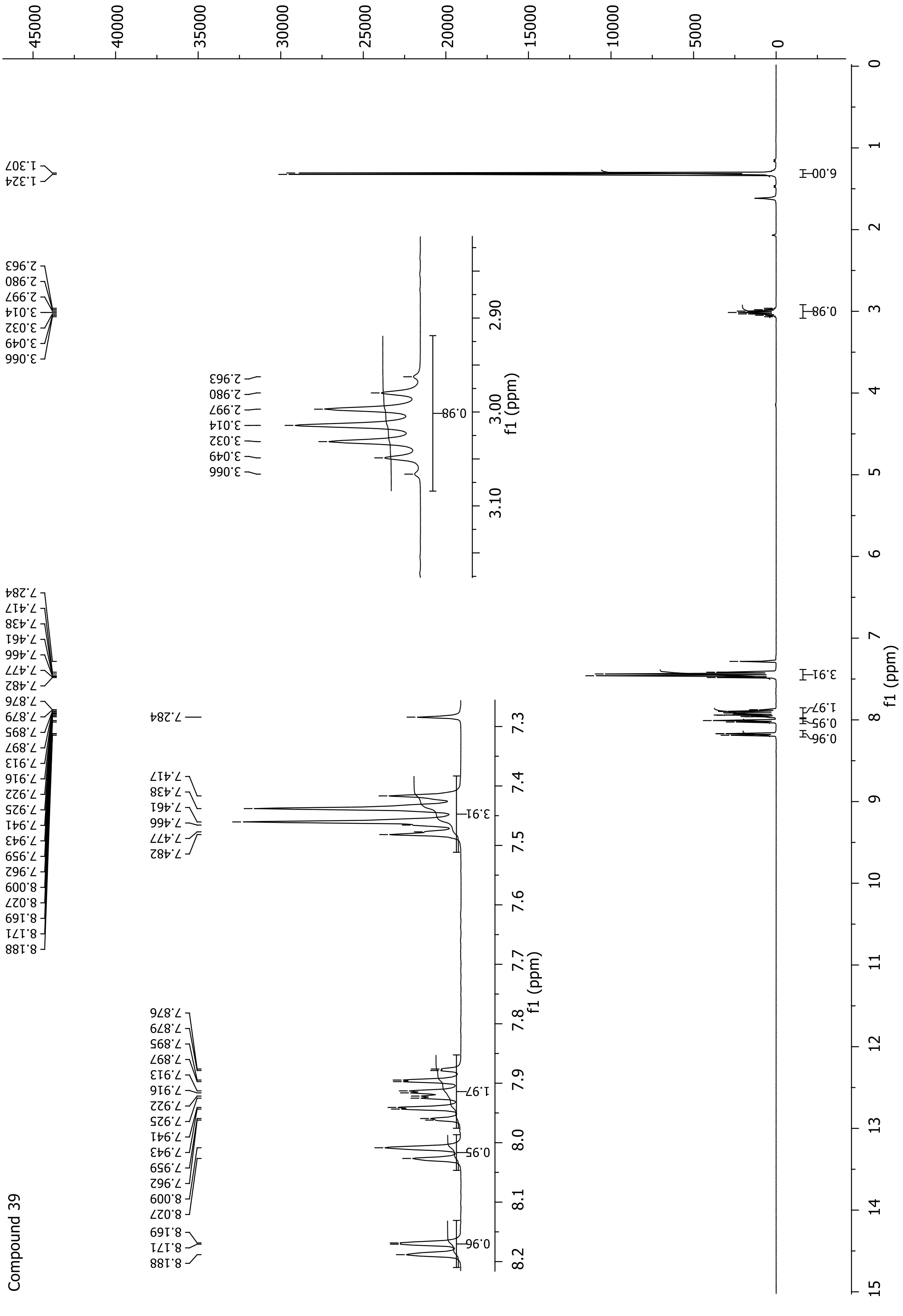

^{13}C-NMR of compound 39

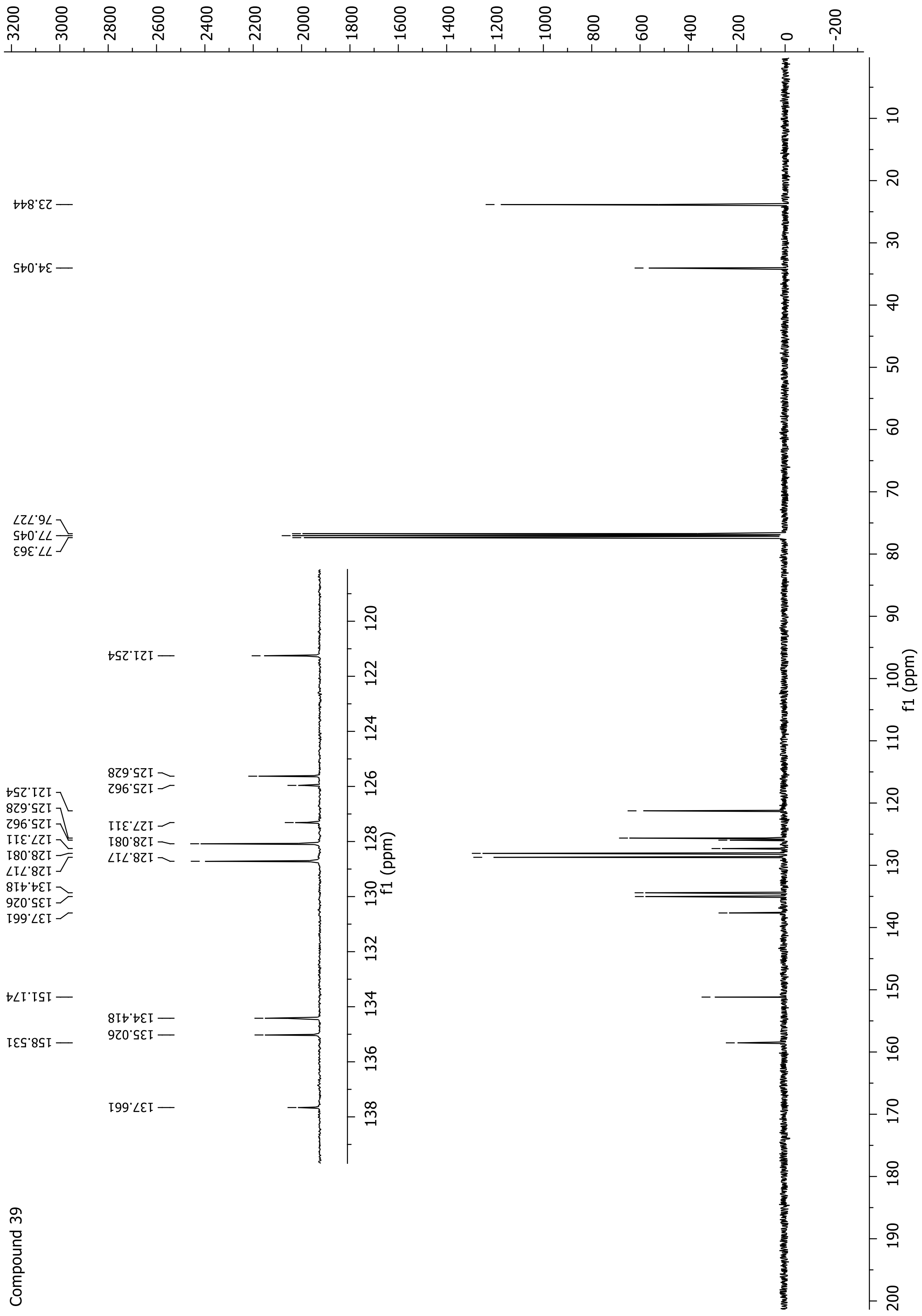

^{1}H-NMR of compound 40

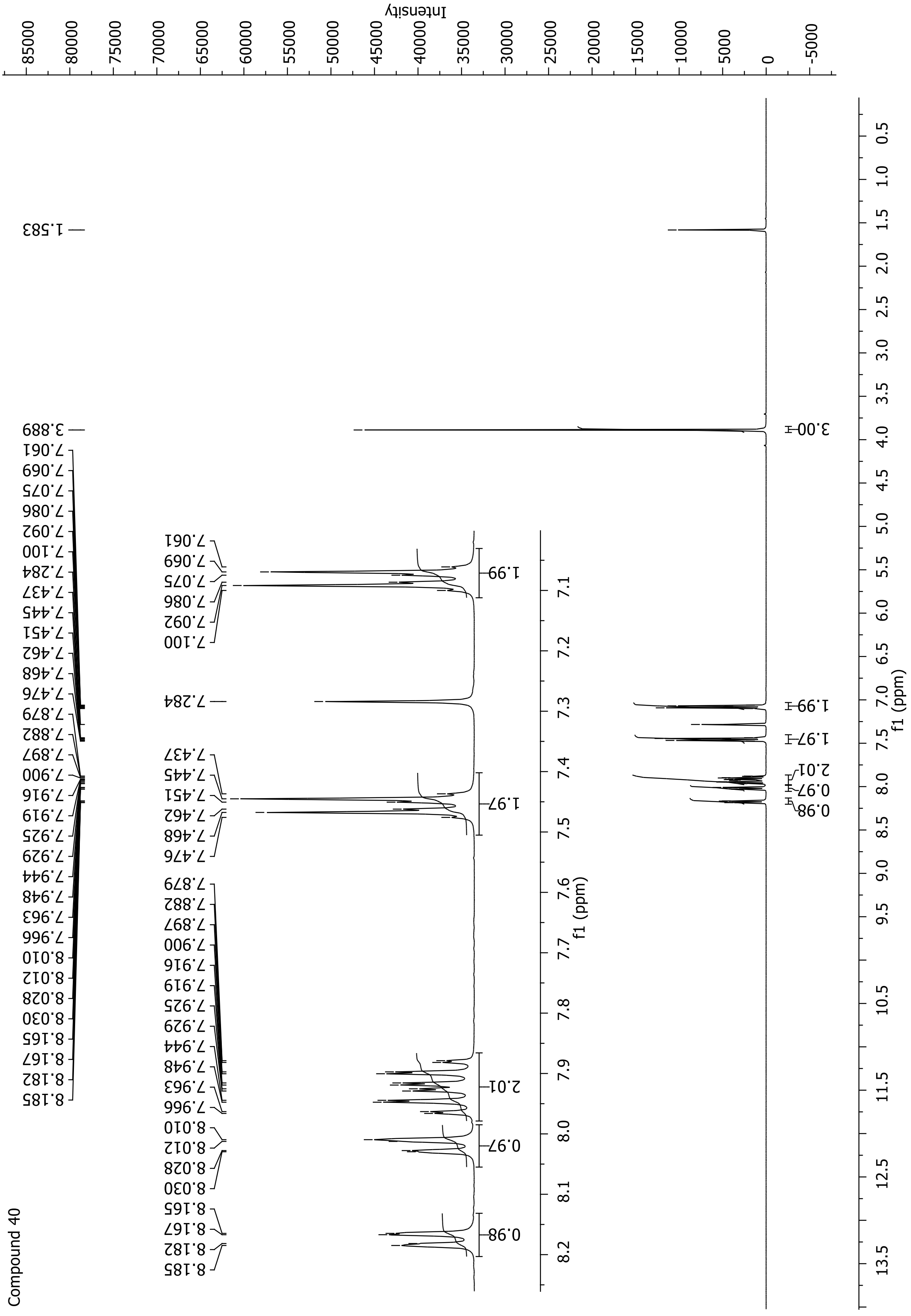

^{1}H-NMR of compound 43

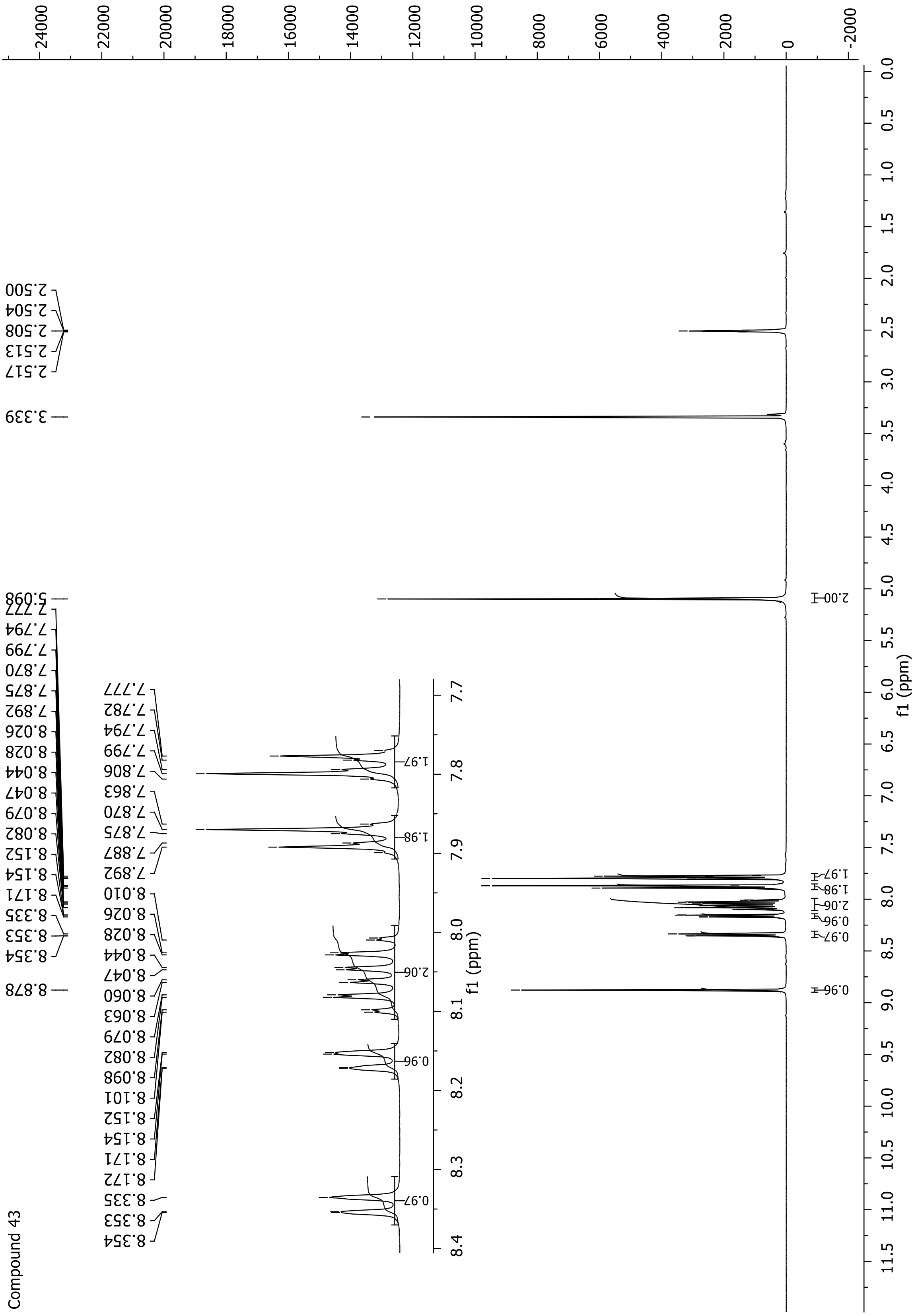

^{13}C-NMR of compound 43

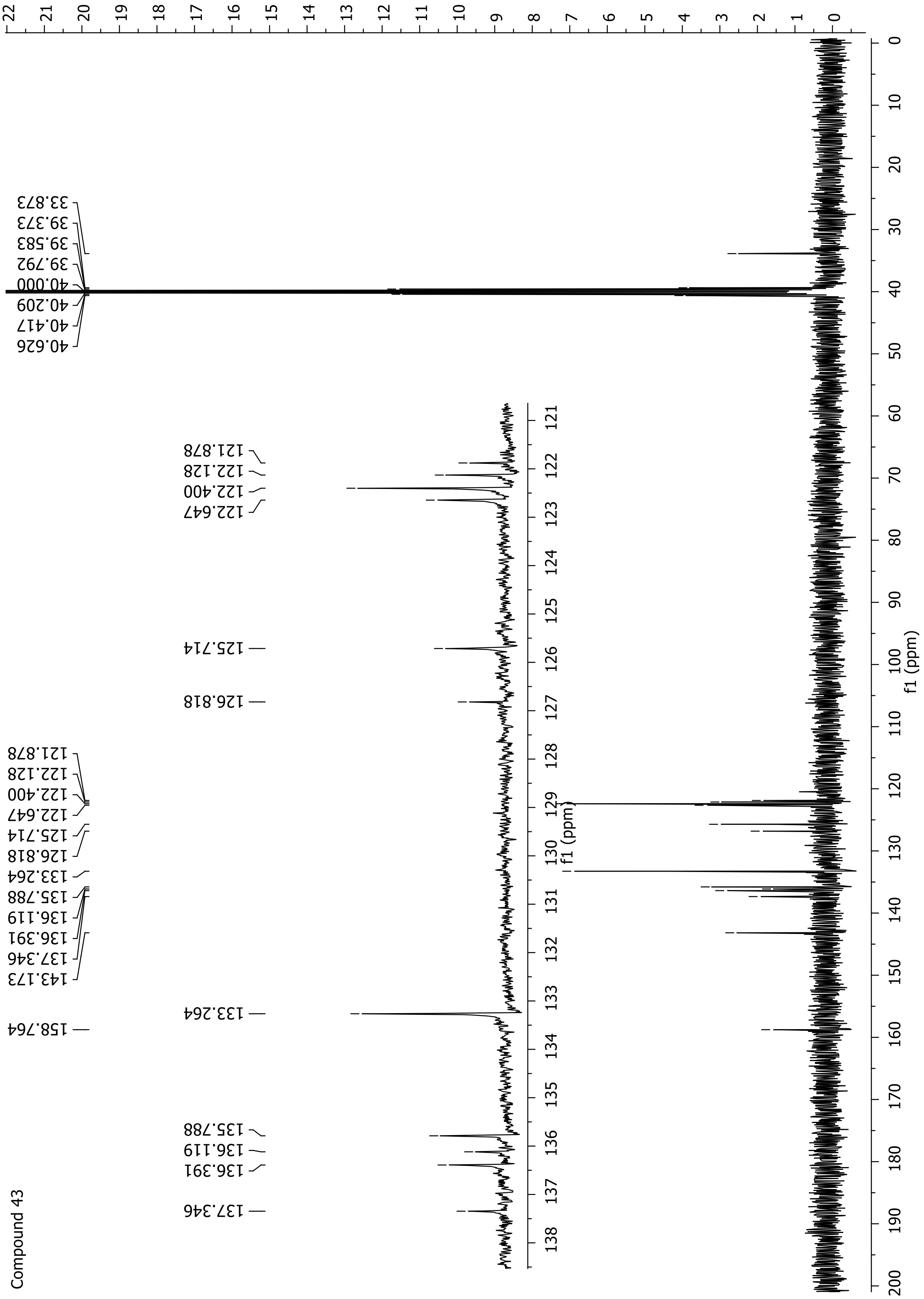

^{1}H-NMR of compound 44

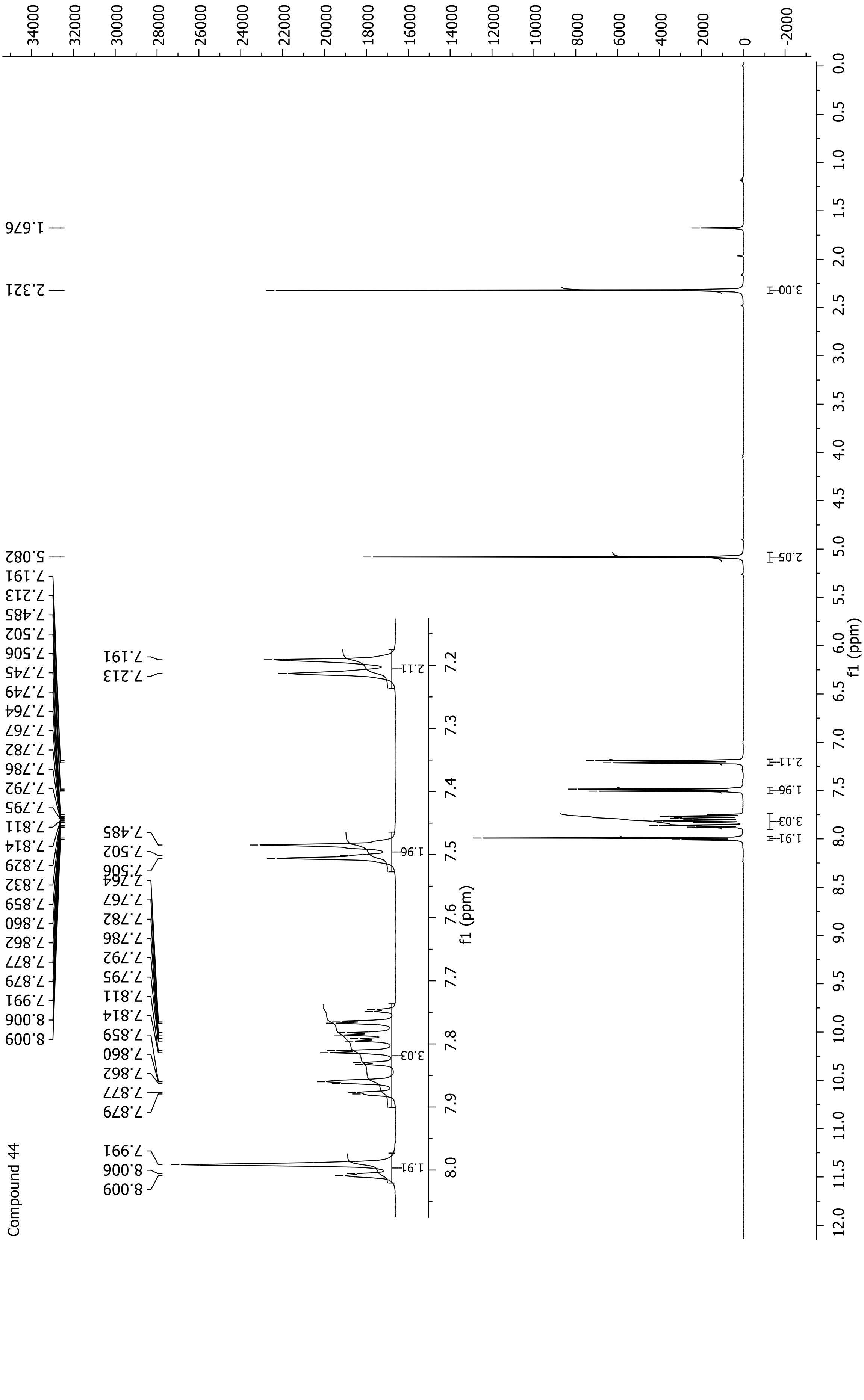

^{13}C-NMR of compound 44

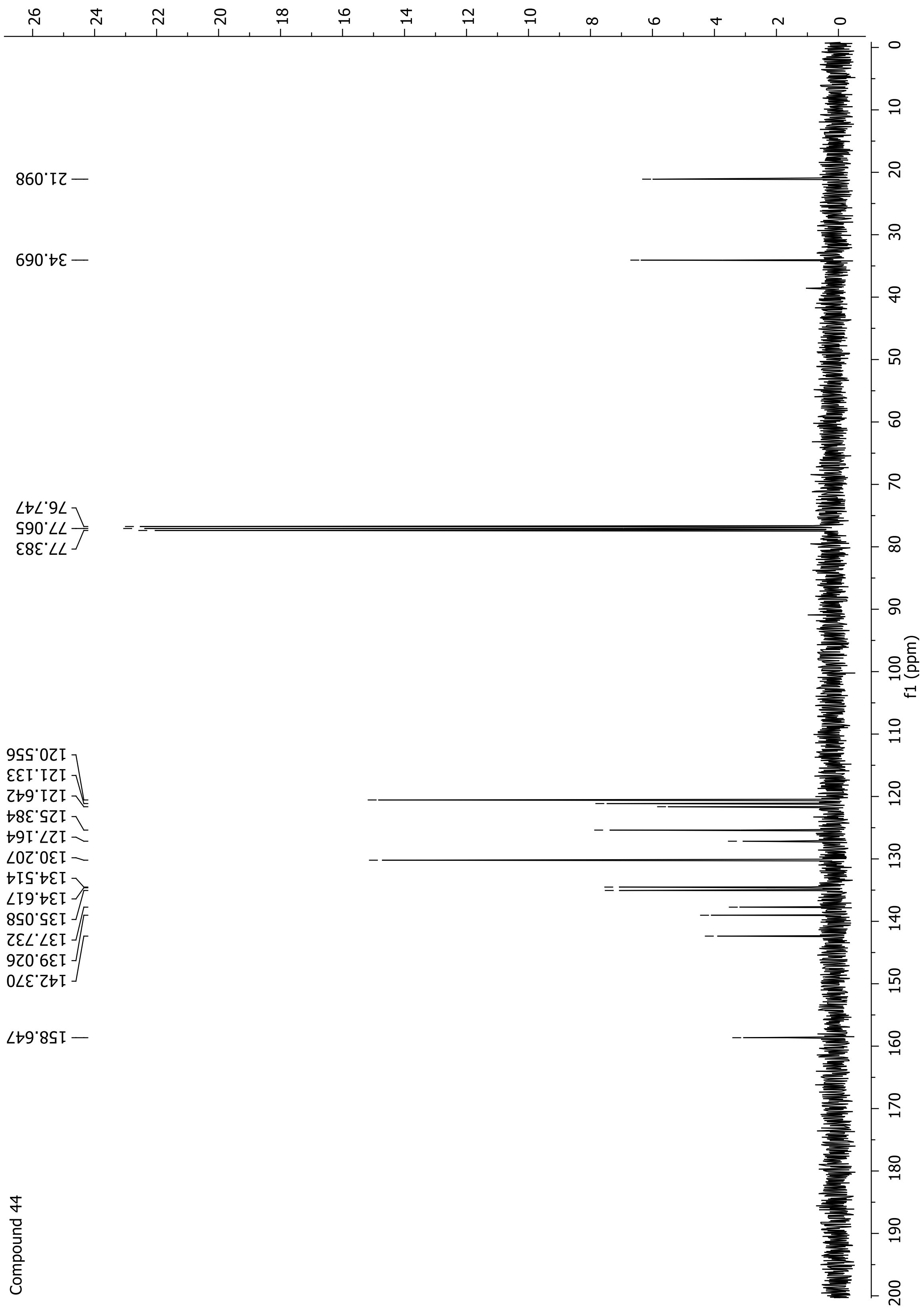

^{1}H-NMR of compound 47

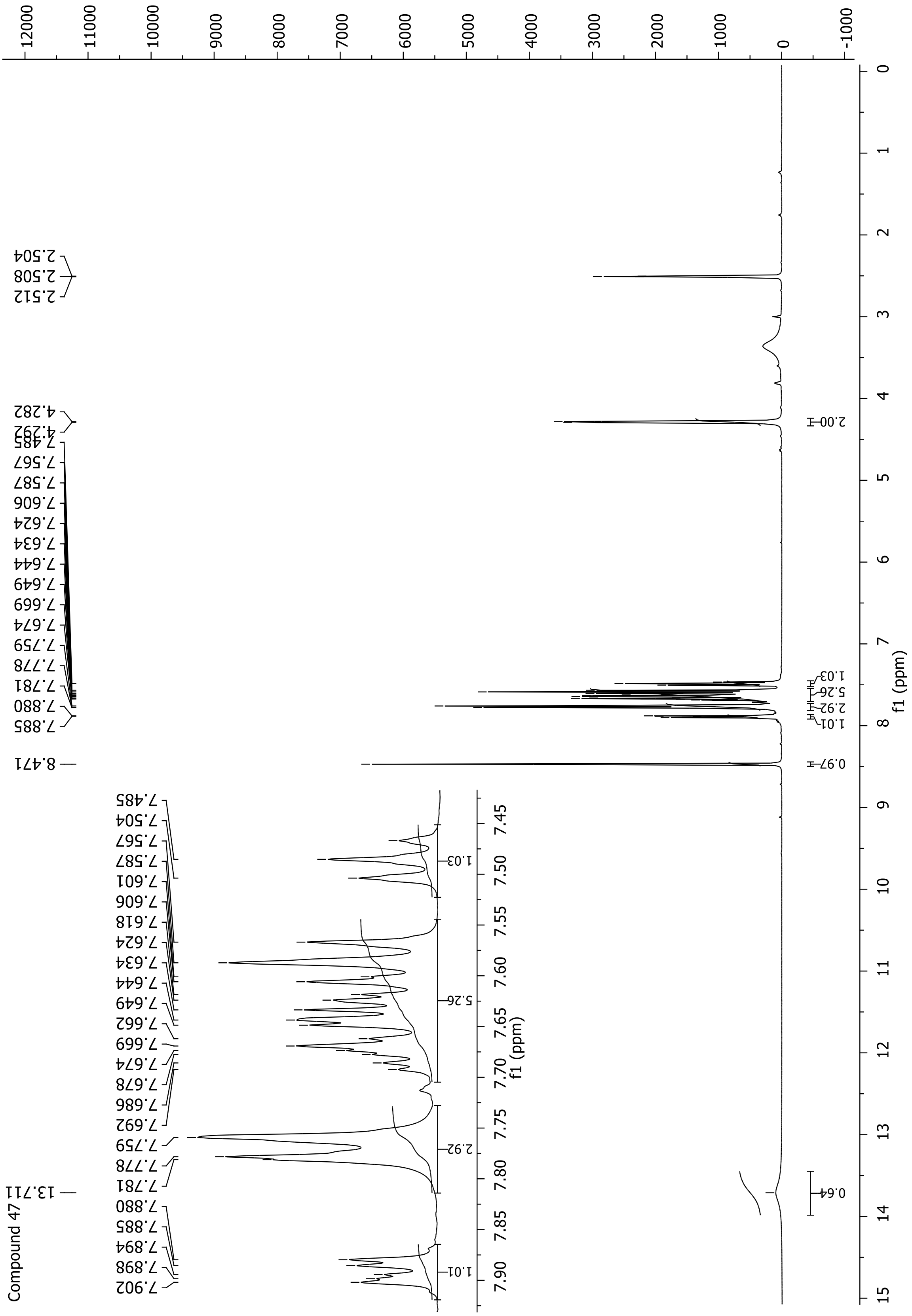

^{13}C-NMR of compound 47

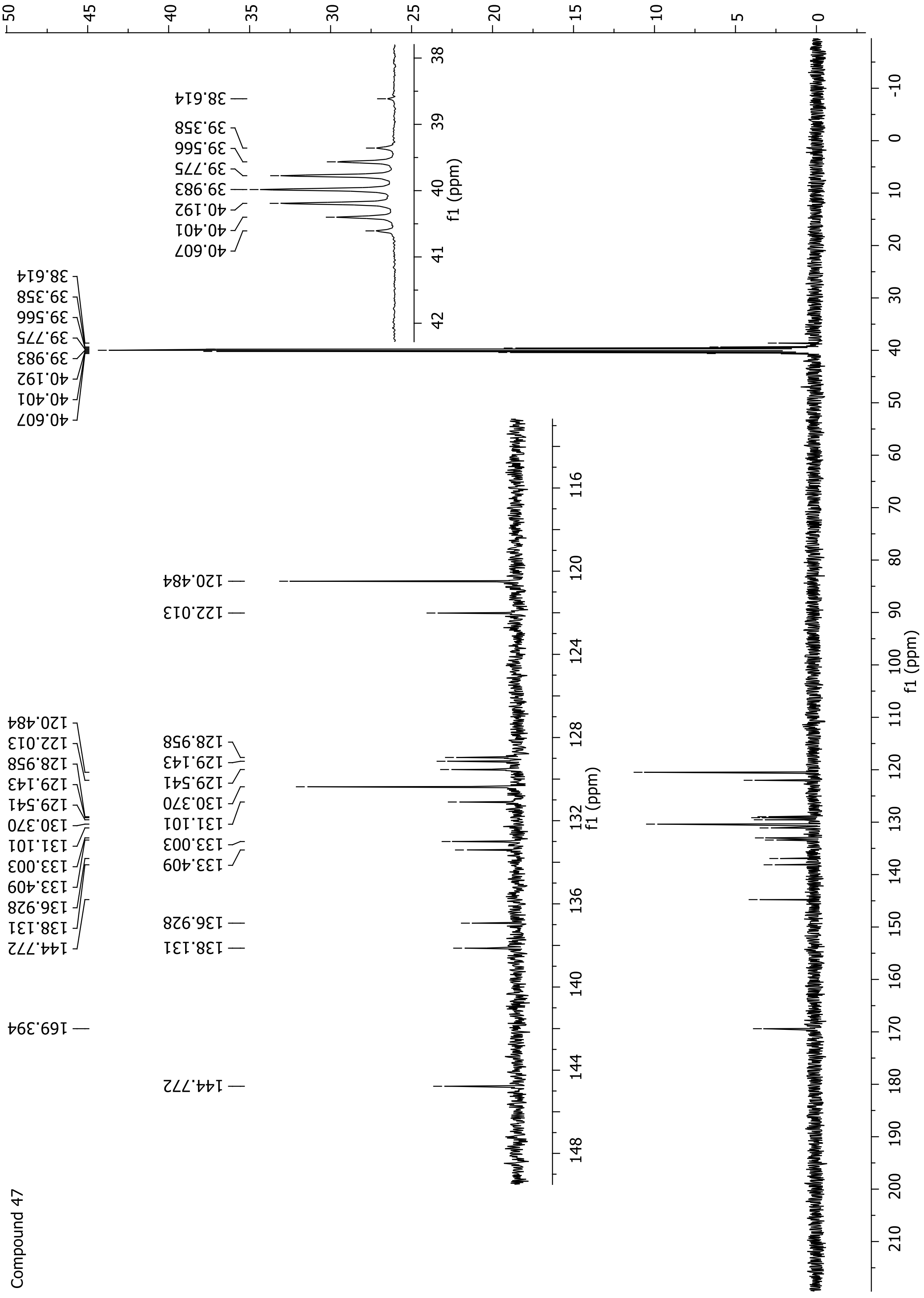

^{1}H-NMR of compound 53

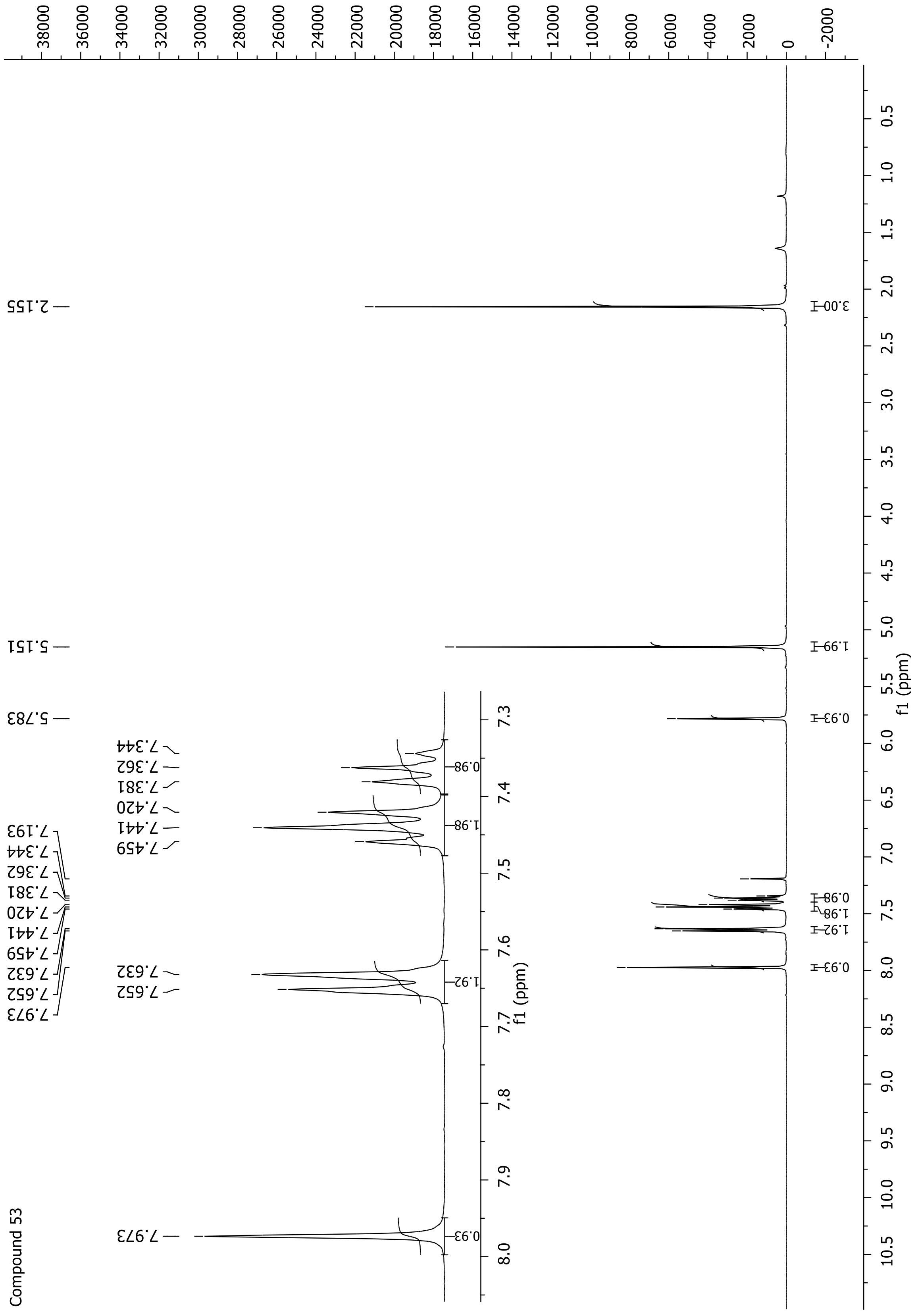

^{13}C-NMR of compound 53

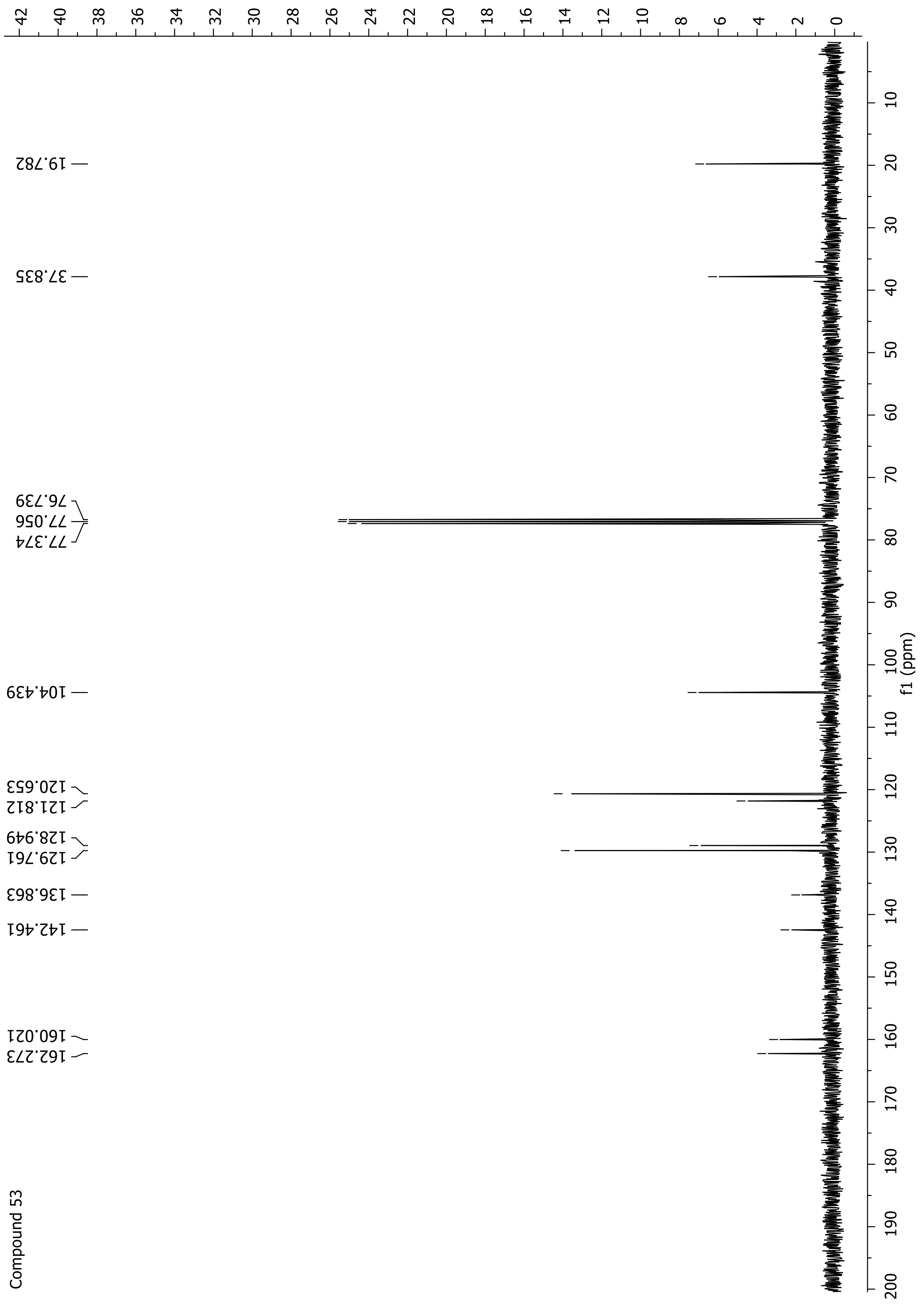

^{1}H-NMR of compound 54

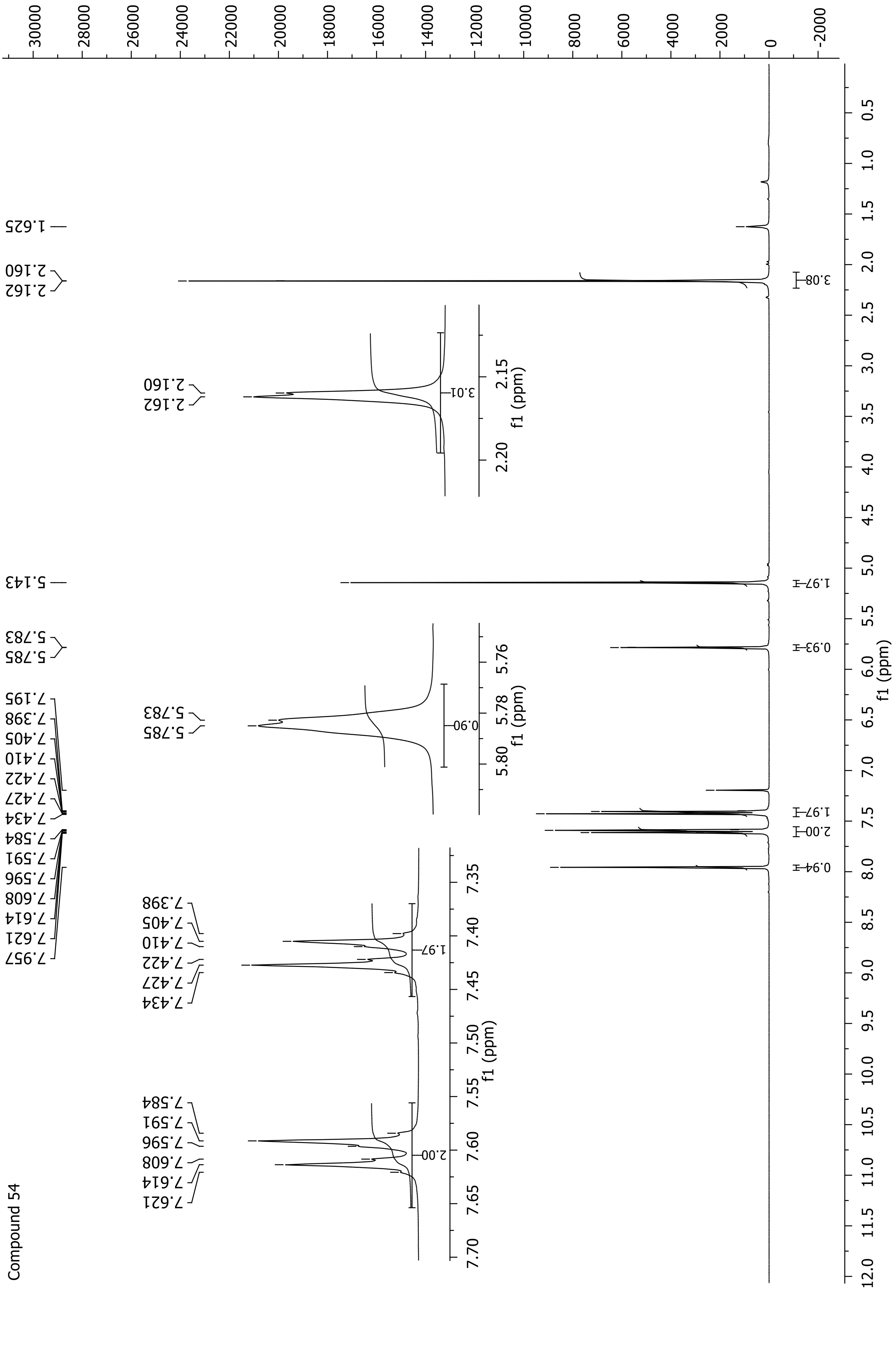

^{13}C-NMR of compound 54

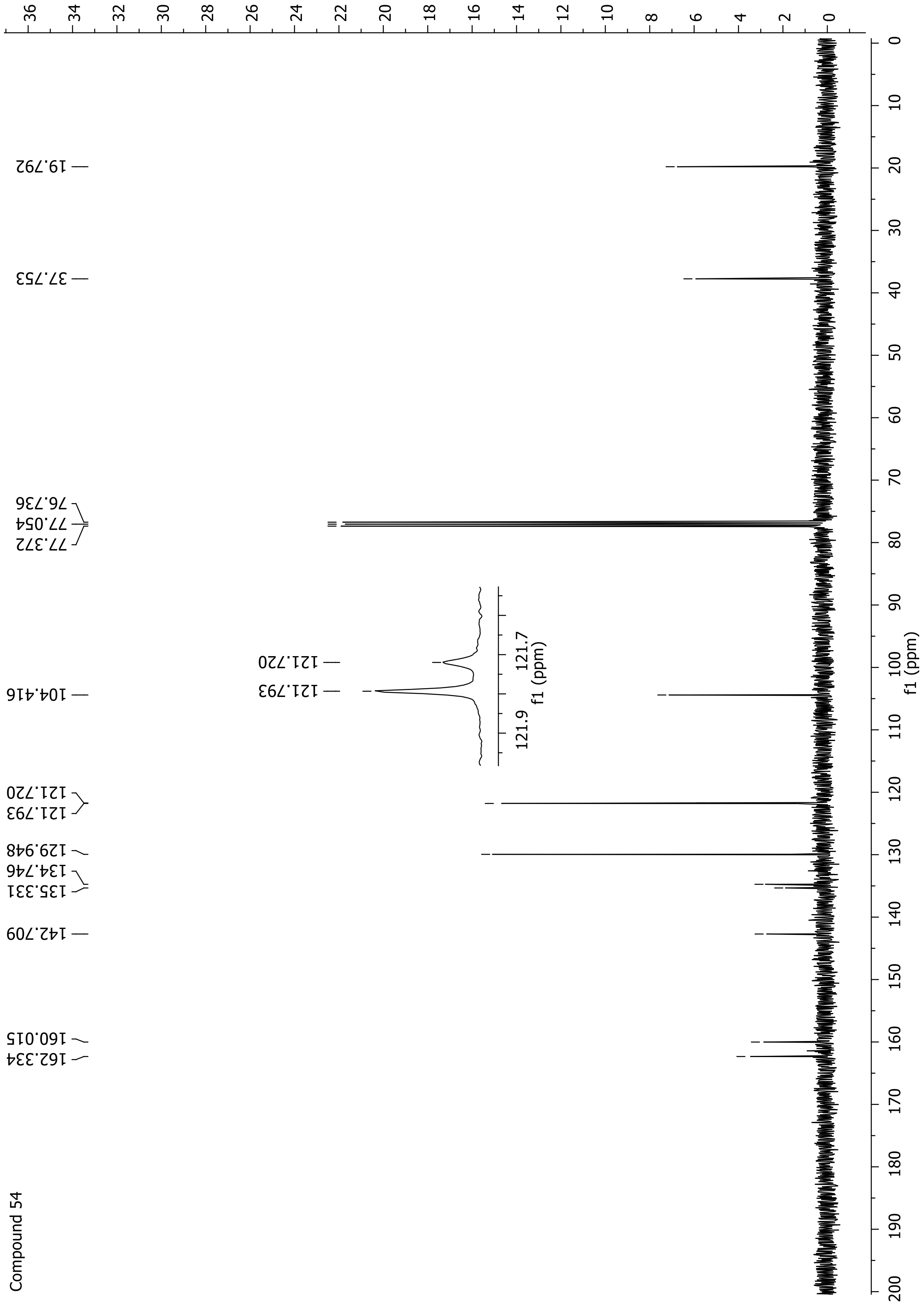

HPLC analysis of the compounds 1-60

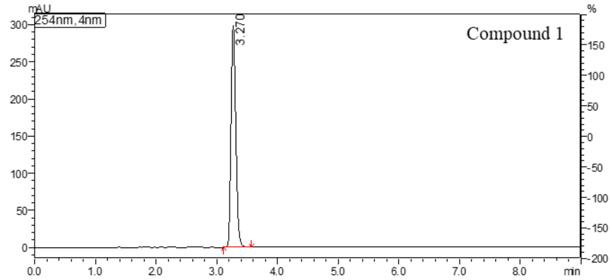

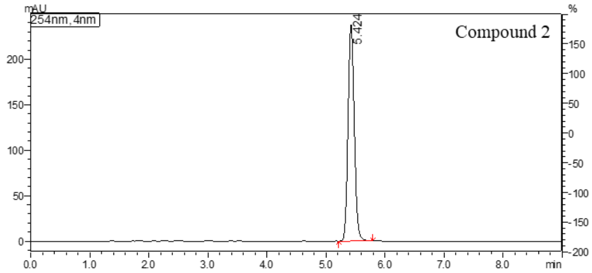

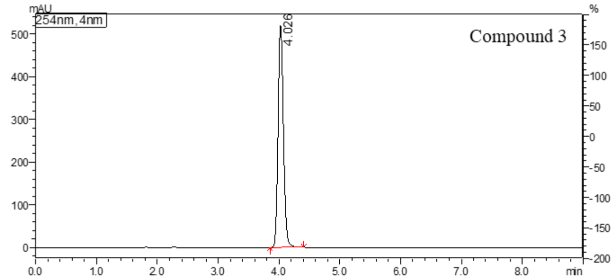

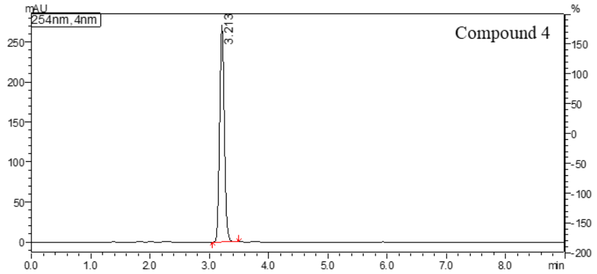

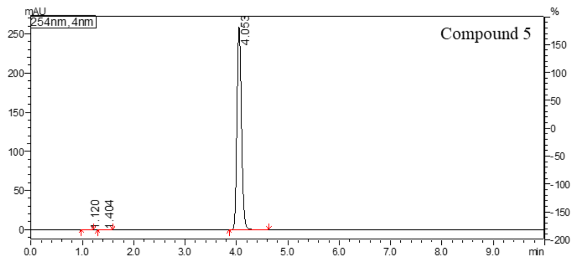

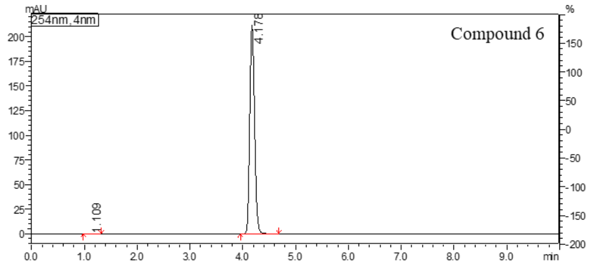

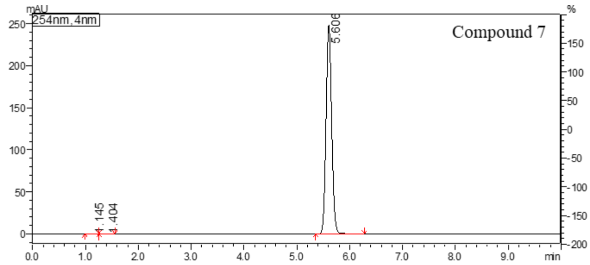

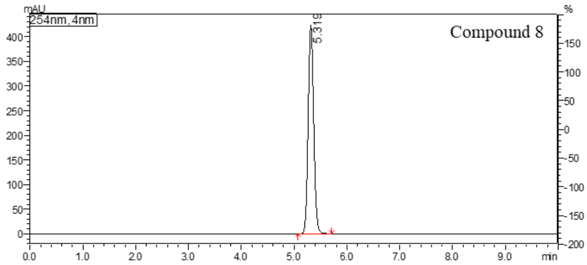

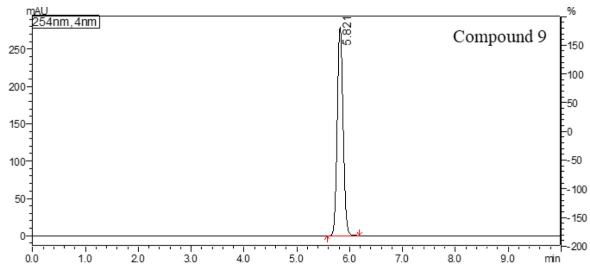

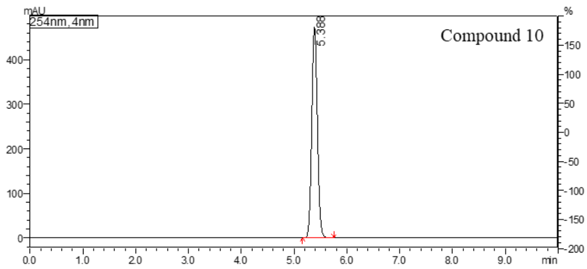

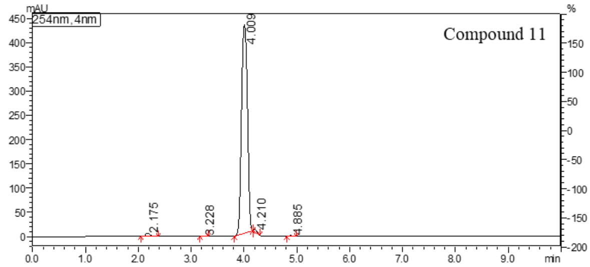

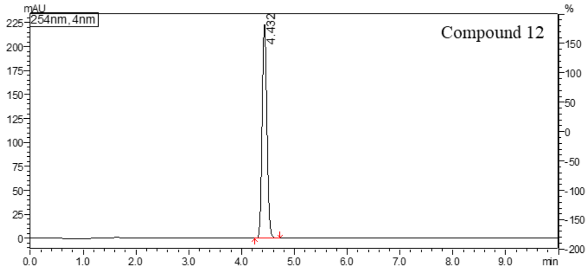

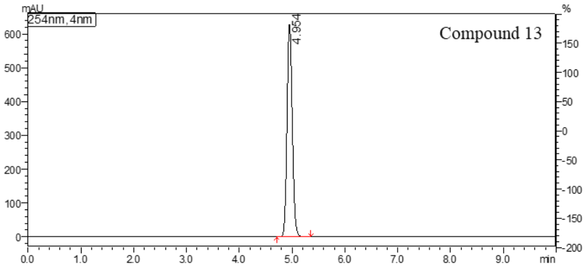
mAU
254nm,4nm
4.954
Compound 13
min
%

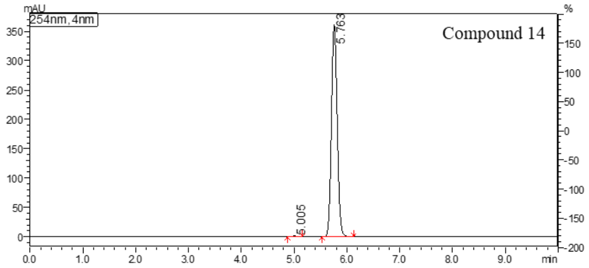
mAU
254nm,4nm
5.005
5.763
Compound 14
min
%

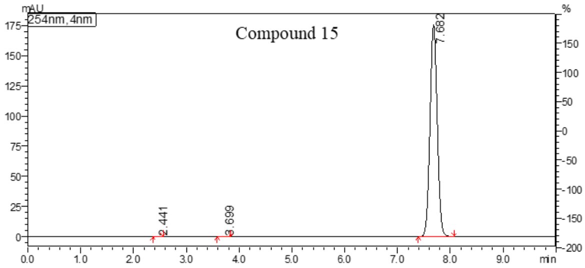
mAU
254nm,4nm
Compound 15
2.441
3.699
7.682
min
%

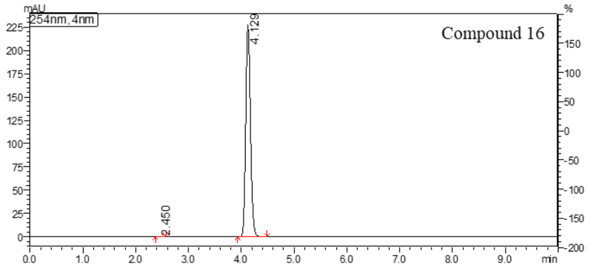
mAU
254nm,4nm
2.450
4.129
Compound 16
min
%

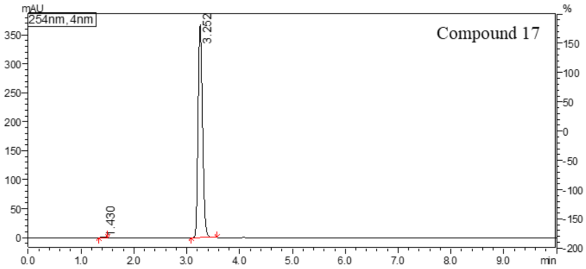
mAU
254nm,4nm
1.430
3.252
Compound 17
min
%

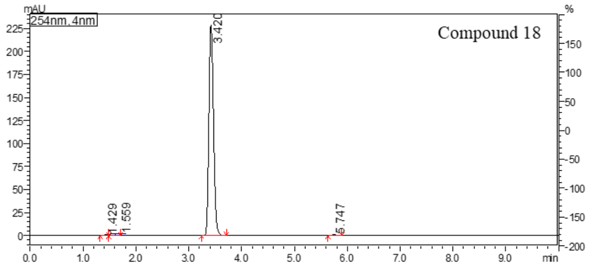
mAU
254nm,4nm
1.429
1.559
3.420
5.747
Compound 18
min
%

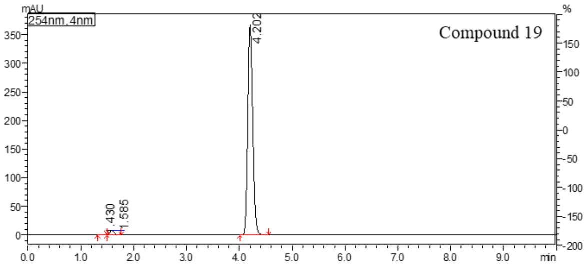
mAU
254nm,4nm
1.430
1.585
4.202
Compound 19
min
%

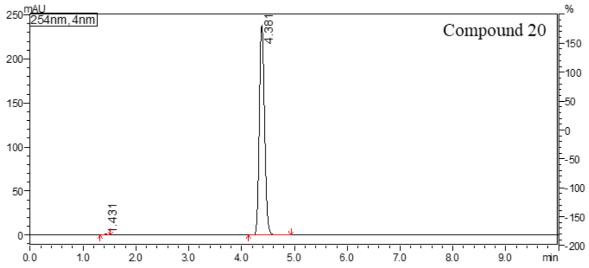
mAU
254nm,4nm
1.431
4.381
Compound 20
min
%

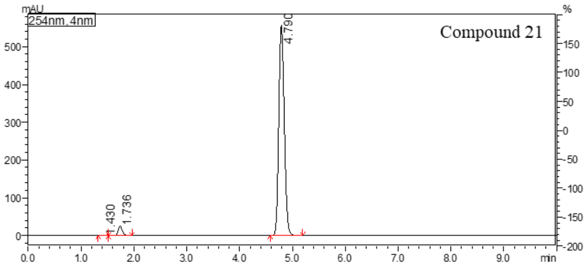
mAU
254nm,4nm
1.430
1.736
4.790
Compound 21
min
%

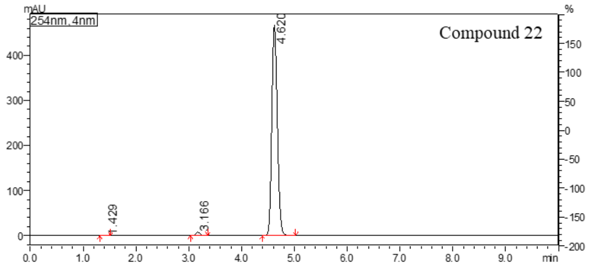
mAU
254nm,4nm
1.429
3.166
4.620
Compound 22
min
%

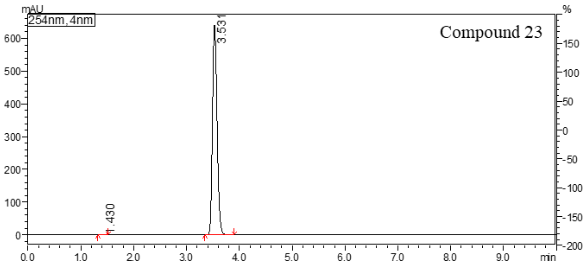
mAU
254nm,4nm
1.430
3.531
Compound 23
min
%

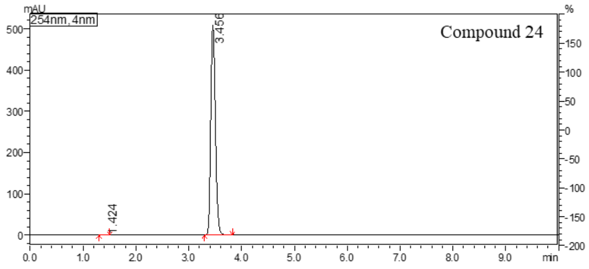
mAU
254nm,4nm
1.424
3.456
Compound 24
min
%

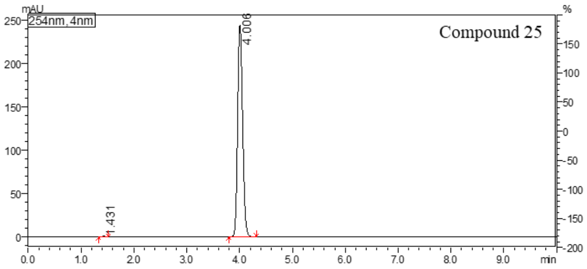
mAU
254nm,4nm
Compound 25
1.431
4.006
min
%

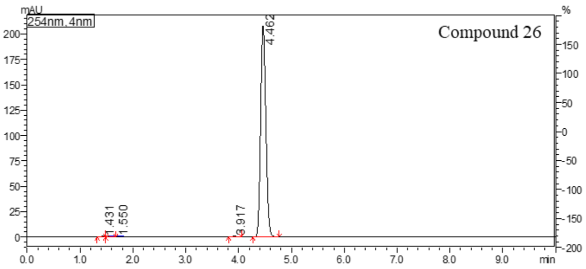
mAU
254nm,4nm
Compound 26
1.431
1.550
3.917
4.462
min
%

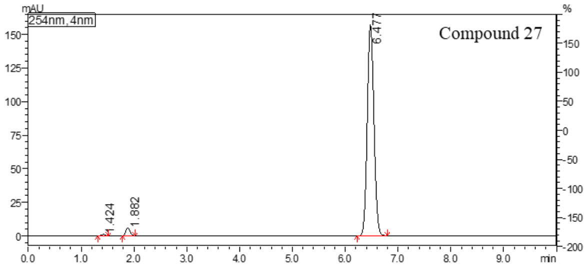
mAU
254nm,4nm
Compound 27
1.424
1.882
6.477
min
%

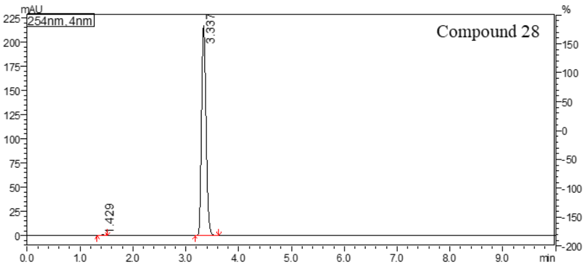
mAU
254nm,4nm
Compound 28
1.429
3.337
min
%

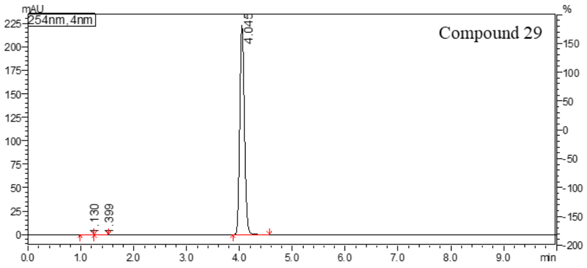
mAU
254nm,4nm
Compound 29
1.130
1.399
4.045
min
%

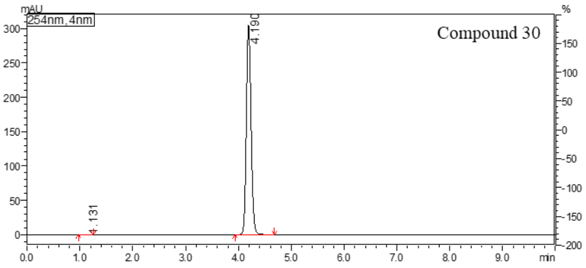
mAU
254nm,4nm
Compound 30
1.131
4.190
min
%

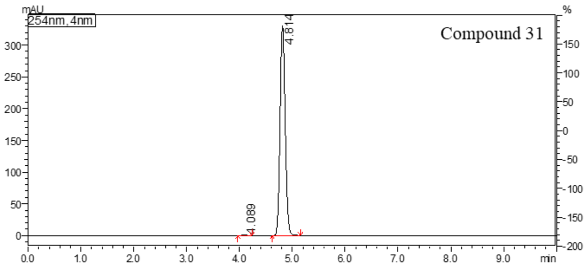
mAU
254nm,4nm
Compound 31
4.089
4.814
min
%

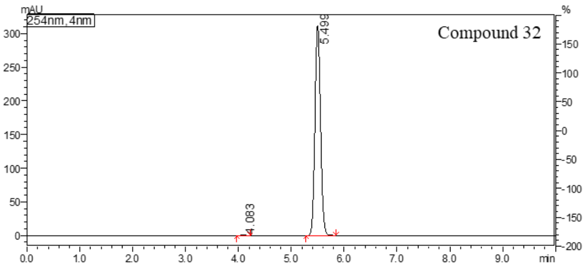
mAU
254nm,4nm
Compound 32
4.083
5.499
min
%

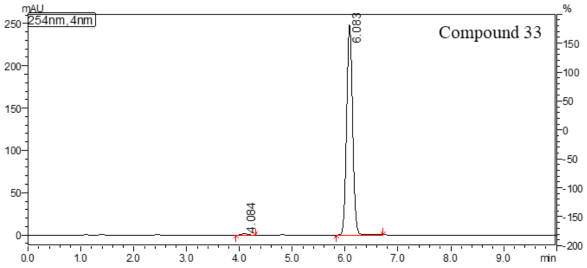
mAU
254nm,4nm
Compound 33
4.084
6.083
min
%

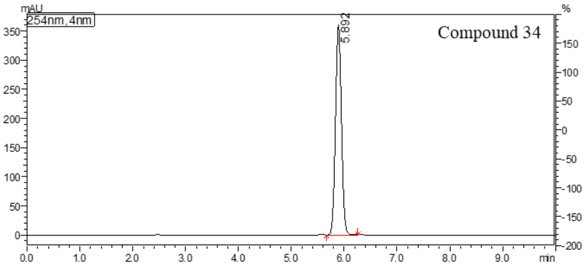
mAU
254nm,4nm
Compound 34
5.892
min
%

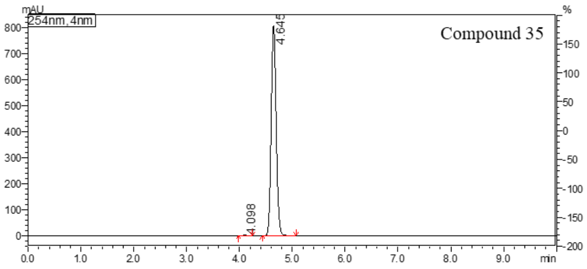
mAU
254nm,4nm
Compound 35
4.098
4.645
min
%

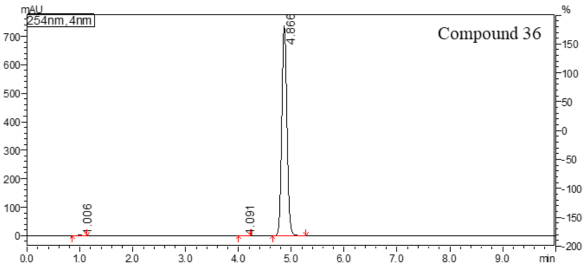
mAU
254nm,4nm
Compound 36
1.006
4.091
4.866
min
%

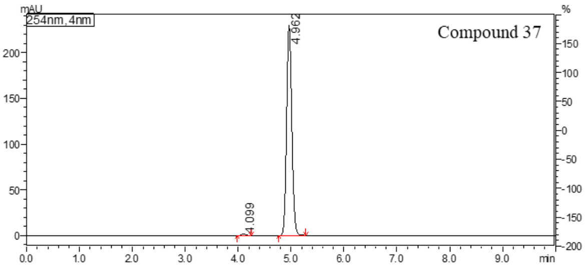
mAU
254nm,4nm
Compound 37
4.099
4.962
%
min

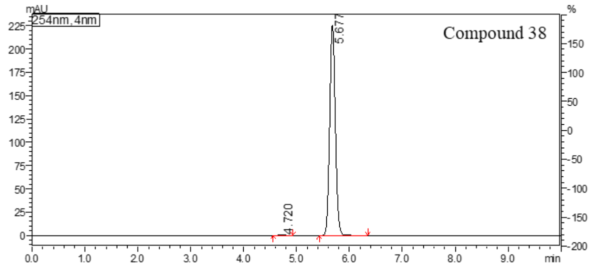
mAU
254nm,4nm
Compound 38
4.720
5.677
%
min

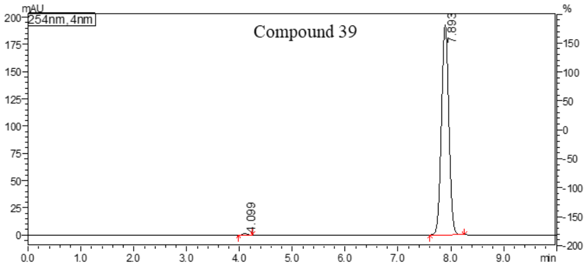
mAU
254nm,4nm
Compound 39
4.099
7.893
%
min

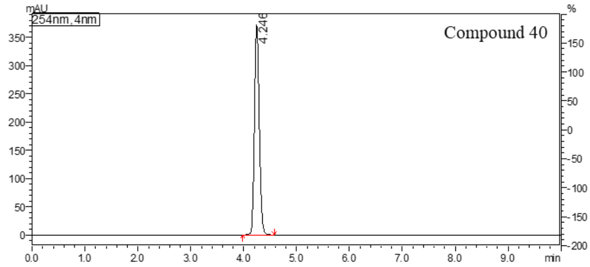
mAU
254nm,4nm
Compound 40
4.246
%
min

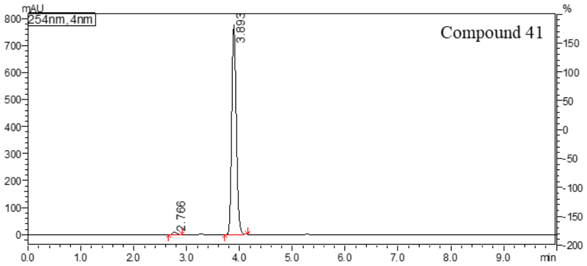
mAU
254nm,4nm
Compound 41
2.766
3.893
%
min

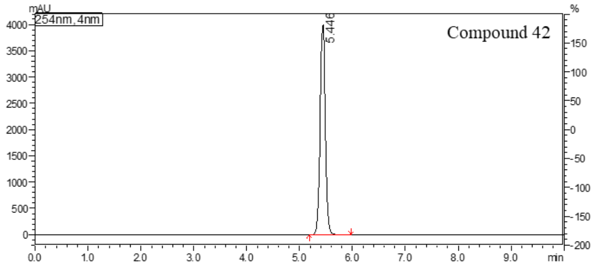
mAU
254nm,4nm
Compound 42
5.446
%
min

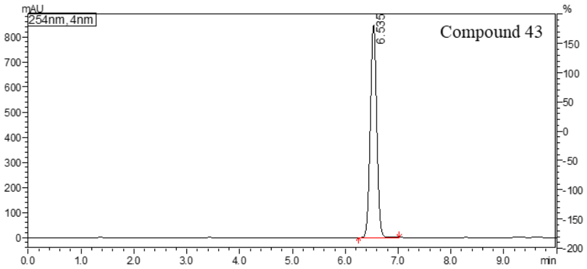
mAU
254nm,4nm
Compound 43
6.535
%
min

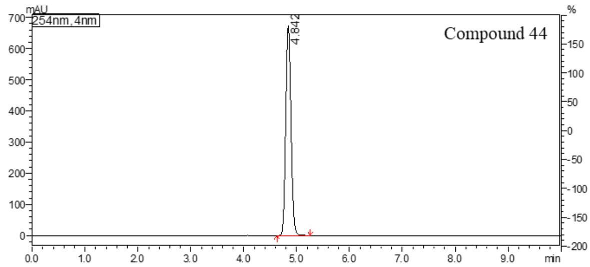
mAU
254nm,4nm
Compound 44
4.842
%
min

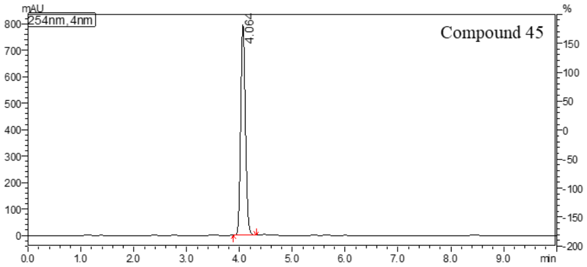
mAU
254nm,4nm
Compound 45
4.064
%
min

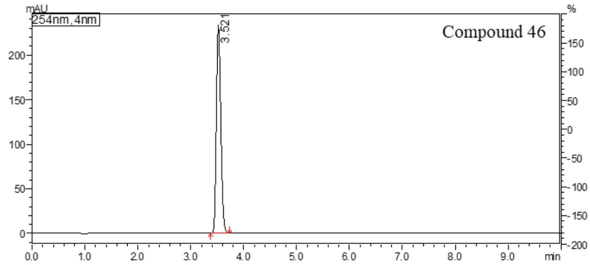
mAU
254nm,4nm
Compound 46
3.521
%
min

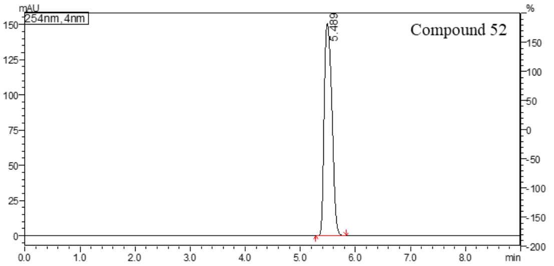
254nm,4nm
Compound 52
5.489
mAU
%
min

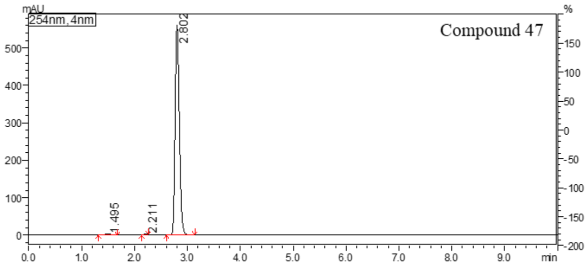
254nm,4nm
Compound 47
1.495
2.211
2.802
mAU
%
min

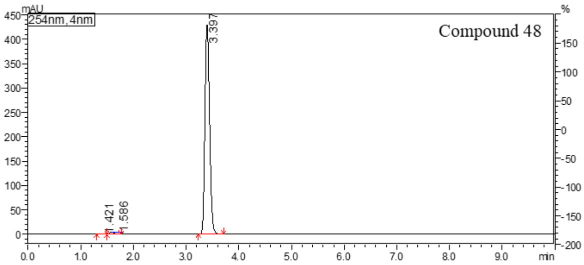
254nm,4nm
Compound 48
1.421
1.586
3.397
mAU
%
min

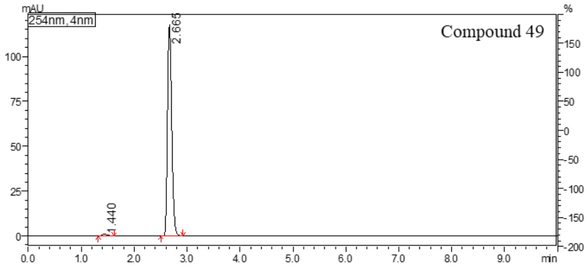
254nm,4nm
Compound 49
1.440
2.665
mAU
%
min

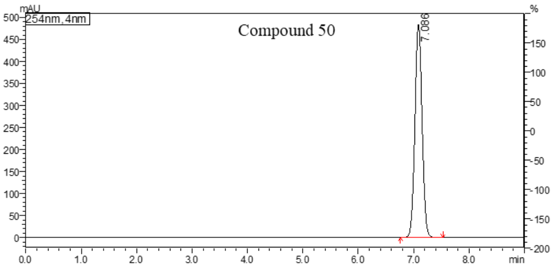
254nm,4nm
Compound 50
7.086
mAU
%
min

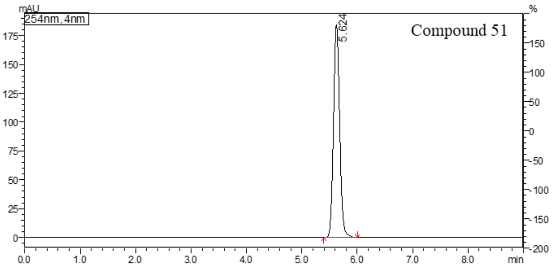
254nm,4nm
Compound 51
5.624
mAU
%
min

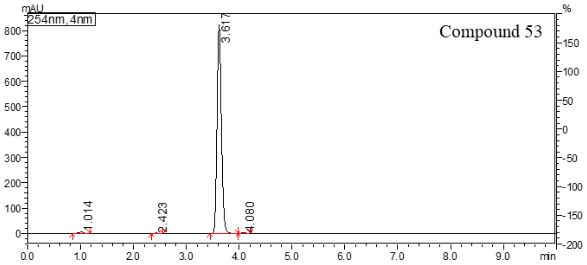
254nm,4nm
Compound 53
1.014
2.423
3.617
4.080
mAU
%
min

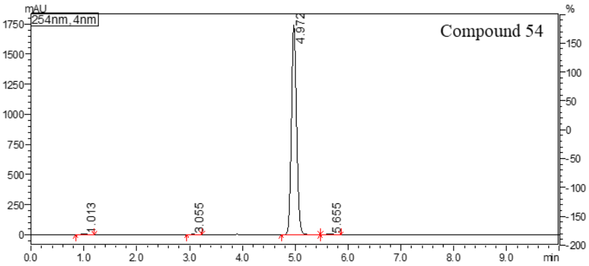
254nm,4nm
Compound 54
1.013
3.055
4.972
5.655
mAU
%
min

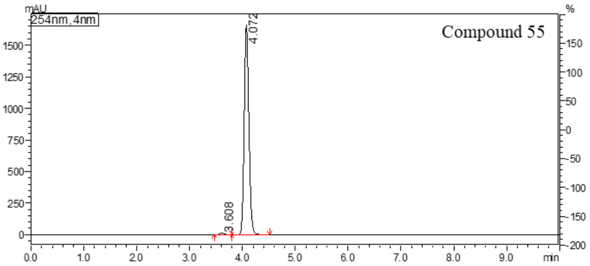
254nm,4nm
Compound 55
3.608
4.072
mAU
%
min

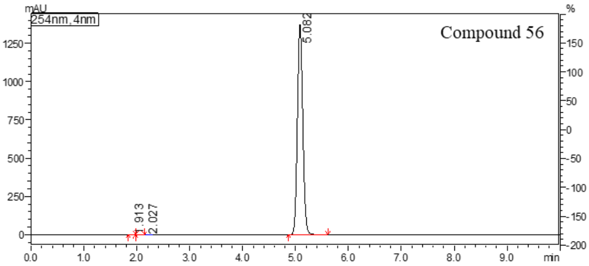
254nm,4nm
Compound 56
1.913
2.027
5.082
mAU
%
min

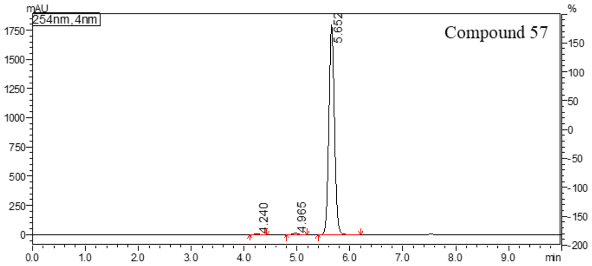
Compound 57
mAU
254nm,4nm
%
4.240
4.965
5.652
min

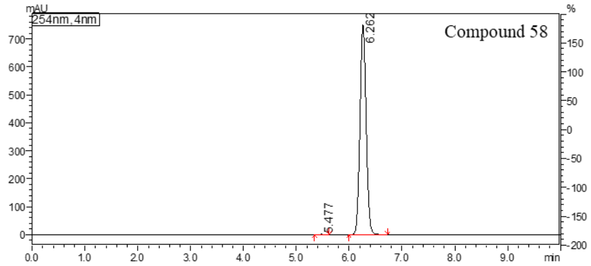
Compound 58
mAU
254nm,4nm
%
5.477
6.262
min

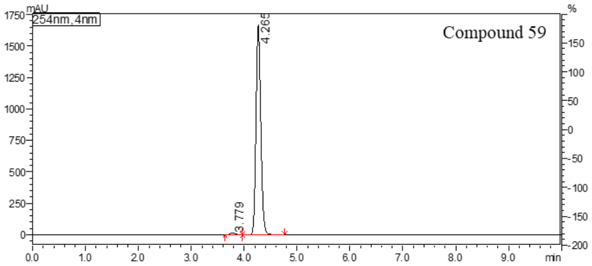
Compound 59
mAU
254nm,4nm
%
3.779
4.265
min

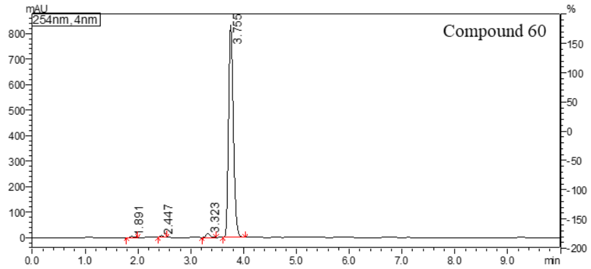
Compound 60
mAU
254nm,4nm
%
1.891
2.447
3.323
3.755
min

References

[1] M. D'Ascenzio, S. Carradori, C. De Monte, D. Secci, M. Ceruso, C.T. Supuran, Design, synthesis and evaluation of *N*-substituted saccharin derivatives as selective inhibitors of tumor-associated carbonic anhydrase XII., Bioorg. Med. Chem. 22 (2014) 1821–31. doi:10.1016/j.bmc.2014.01.056.

[2] C. De Monte, S. Carradori, D. Secci, M. D'Ascenzio, D. Vullo, M. Ceruso, C.T. Supuran, Cyclic tertiary sulfamates: Selective inhibition of the tumor-associated carbonic anhydrases IX and XII by *N*- and *O*-substituted acesulfame derivatives, Eur. J. Med. Chem. 84 (2014) 240–246. doi:10.1016/j.ejmech.2014.07.014.

[3] A. Nocentini, M. Ferraroni, F. Carta, M. Ceruso, P. Gratteri, C. Lanzi, E. Masini, C.T. Supuran, Benzenesulfonamides Incorporating Flexible Triazole Moieties Are Highly Effective Carbonic Anhydrase Inhibitors: Synthesis and Kinetic, Crystallographic, Computational, and Intraocular Pressure Lowering Investigations, J. Med. Chem. 59 (2016) 10692–10704. doi:10.1021/acs.jmedchem.6b01389.

[4] C. Shao, X. Wang, Q. Zhang, S. Luo, J. Zhao, Y. Hu, Acid–Base Jointly Promoted Copper(I)-Catalyzed Azide–Alkyne Cycloaddition, J. Org. Chem. 76 (2011) 6832–6836. doi:10.1021/jo200869a.

[5] K. V. Kutonova, M.E. Trusova, P. Postnikov, V.D. Filimonov, J. Parello, A simple and effective synthesis of aryl azides via arenediazonium tosylates, Synth. 45 (2013) 2706–2710. doi:10.1055/s-0033-1339648.
